# TGFβ promotes fibrosis by MYST1-dependent epigenetic regulation of autophagy

**DOI:** 10.1038/s41467-021-24601-y

**Published:** 2021-07-20

**Authors:** Ariella Zehender, Yi-Nan Li, Neng-Yu Lin, Adrian Stefanica, Julian Nüchel, Chih-Wei Chen, Hsiao-Han Hsu, Honglin Zhu, Xiao Ding, Jingang Huang, Lichong Shen, Andrea-Hermina Györfi, Alina Soare, Simon Rauber, Christina Bergmann, Andreas Ramming, Markus Plomann, Beate Eckes, Georg Schett, Jörg H. W. Distler

**Affiliations:** 1 Department of Internal Medicine 3—Rheumatology and Immunology, Friedrich-Alexander-University Erlangen-Nürnberg (FAU) and University Hospital Erlangen, Erlangen, Germany; 2 Deutsches Zentrum für Immuntherapie, Friedrich Alexander University Erlangen-Nuremberg and Universitaetsklinikum Erlangen, Erlangen, Germany; 3 Graduate Institute of Anatomy and Cell Biology, College of Medicine, National Taiwan University, Taipei, Taiwan; 4 Center for Biochemistry, University of Cologne, Faculty of Medicine, Cologne, Germany; 5 Department of Rheumatology, Xiangya Hospital, Central South University, Changsha, Hunan China; 6 Translational Matrix Biology, University of Cologne, Faculty of Medicine, Cologne, Germany; 7 Cologne Excellence Cluster on Cellular Stress Responses in Aging-Associated Diseases (CECAD), Cologne, Germany

**Keywords:** Autophagy, Systemic sclerosis, Rheumatology

## Abstract

Activation of fibroblasts is essential for physiological tissue repair. Uncontrolled activation of fibroblasts, however, may lead to tissue fibrosis with organ dysfunction. Although several pathways capable of promoting fibroblast activation and tissue repair have been identified, their interplay in the context of chronic fibrotic diseases remains incompletely understood. Here, we provide evidence that transforming growth factor-β (TGFβ) activates autophagy by an epigenetic mechanism to amplify its profibrotic effects. TGFβ induces autophagy in fibrotic diseases by SMAD3-dependent downregulation of the H4K16 histone acetyltransferase MYST1, which regulates the expression of core components of the autophagy machinery such as ATG7 and BECLIN1. Activation of autophagy in fibroblasts promotes collagen release and is both, sufficient and required, to induce tissue fibrosis. Forced expression of MYST1 abrogates the stimulatory effects of TGFβ on autophagy and re-establishes the epigenetic control of autophagy in fibrotic conditions. Interference with the aberrant activation of autophagy inhibits TGFβ-induced fibroblast activation and ameliorates experimental dermal and pulmonary fibrosis. These findings link uncontrolled TGFβ signaling to aberrant autophagy and deregulated epigenetics in fibrotic diseases and may contribute to the development of therapeutic interventions in fibrotic diseases.

## Abstract (web summary)

Uncontrolled activation of fibroblasts contributes to tissue fibrosis and organ dysfunction. Here the authors demonstrate that the epigenetic control of autophagy is disturbed by a TGFβ-dependent downregulation of MYST1 in systemic sclerosis patients. Restoration of the epigenetic control of autophagy reduces fibroblast activation and ameliorates fibrotic tissue remodeling.

## Introduction

Fibrotic diseases are characterized by excessive deposition of extracellular matrix with perturbation of the physiological tissue architecture. Fibrotic tissue remodeling imposes a major burden on modern societies and has been estimated to contribute to up to 45% of deaths in the developed world^1^. In addition to high mortality, many fibrotic diseases are also associated with severe morbidity and often cause lifelong disability, which results in socioeconomic costs in the order of tens of billions of dollars per year^2^. Fibrotic tissue remodeling can occur in response to defined stimuli such as trauma, infection or tumors, whereas the initiating trigger remains unknown in other cases^3^. Those idiopathic fibrotic diseases can affect virtually every organ system of the human body. Systemic sclerosis (SSc) is a prototypical example of an idiopathic fibrotic disease with high mortality and morbidity^4^. SSc most commonly affects the skin and lungs, but may also involve other internal organs such as the heart and the intestinal tract. SSc and other fibrotic diseases are characterized by aberrant activation of resident fibroblasts that display a persistently activated myofibroblast phenotype. While myofibroblasts are only transiently observed during normal wound healing, they persist in fibrotic diseases, thereby leading to excessive repair responses that culminate in tissue fibrosis^5^.

Although the molecular mechanisms underlying the aberrant fibroblast activation in fibrotic tissues remain incompletely understood, overwhelming evidence highlights that transforming growth factor-β (TGFβ) can promote fibroblast activation and myofibroblast differentiation^6^. TGFβ signaling is upregulated in fibrotic diseases with persistent activation of intracellular downstream mediators and a TGFβ biased gene expression signature in affected tissues^7^. Moreover, forced activation of TGFβ signaling induces a myofibroblast phenotype in resting fibroblasts and systemic fibrosis in mice^8^. Increased TGFβ signaling is not only sufficient, but also required for fibrosis as inhibition of TGFβ or its receptors ameliorates fibrosis in multiple experimental models^6^.

Epigenetic alterations are increasingly recognized as drivers of progression of fibrotic diseases^9,10^. The expression of several epigenetic regulators including DNA methyltransferases, non-coding RNAs, histone demethylases, and histone acetyltransferases is deregulated in fibrotic diseases^10–12^. The resulting changes in the epigenetic code skew the transcriptome towards a profibrotic gene expression profile, thereby enabling effector cells such as fibroblasts to maintain their activated phenotype even in the absence of exogenous stimuli. Accumulating evidence from preclinical studies and first proof-of-concept studies in patients with fibrotic disease demonstrate that targeting the aberrant epigenetic modifications may offer potential for the treatment of fibrotic diseases^10,13–21^.

Macroautophagy (often simply referred to as autophagy) is an evolutionarily conserved catabolic process allowing cells to degrade unnecessary or dysfunctional cellular organelles, which is an important source of nutrients during starvation or in response to cellular stress^22^. Moreover, components of the autophagy machinery (ATGs) are involved in secretion (secretory autophagy), including unconventional secretion of proteins^23–26^. Recently, LC3 has been shown to mediate the loading of protein and RNA cargoes into extracellular vesicles for secretion into the extracellular space^27^. Autophagy also supports other secretory pathways, such as constitutive and regulated secretion^28^ and may thus offers an alternative trafficking route for integral membrane proteins to the plasma membrane^29^.

Autophagy is initiated by formation of an isolation membrane, which elongates to sequester damaged organelles in autophagosomes. These autophagosomes subsequently fuse with lysosomes to initiate the degradation of the engulfed material^30^. Autophagy is tightly regulated by a complex cascade of signaling events that involve a panel of autophagy-related proteins (ATG). Key regulators that are also used as markers for autophagy include BECLIN1, the human orthologue of yeast ATG6, which promotes the formation of the isolation membrane and ATG7, which is required for elongation of the isolation membrane^31–34^. In parallel to the identification of key effector molecules of the autophagocytic machinery, dysregulation of autophagy has been linked to various pathophysiological conditions such as aging, cancer, inflammation and autoimmunity^35,36^. Aberrant activation of autophagy has also been implicated in the pathogenesis of fibrotic diseases. Depending on the primary effector cells in the affected organs and the kinetics of the triggering insult, pro- as well as antifibrotic effects of autophagy have been reported, suggesting a context-dependent outcome^37–45^. Several stimuli known to be present in SSc and other fibrotic diseases such as profibrotic and pro-inflammatory cytokines or hypoperfusion with subsequent tissue hypoxia and impaired nutrient supply are known to be capable of activating autophagy^46,47^. Recent evidence demonstrates that regulation of autophagy can also occur at an epigenetic level. The expression of several ATGs is regulated by acetylation of histone H4 lysine 16^48–50^, thus allowing to modulate the threshold for autophagy in individual cells via histone acetyltransferases. However, whether epigenetic alterations contribute to deregulation of autophagy in pathologic contexts has not yet been determined.

In the present study, we aimed to characterize the regulation of autophagy in fibrosis, to investigate, whether the epigenetic alterations in fibroblasts promote autophagy and to analyze whether targeting of autophagy in fibroblasts may prevent their aberrant activation in fibrotic diseases. We demonstrate that aberrant TGFβ signaling in fibrotic diseases perturbs the epigenetic regulation of autophagy. Re-establishment of adequate H4K16 acetylation inhibits autophagy, limits the profibrotic effects of TGFβ and ameliorates dermal and pulmonary fibrosis. Restoration of adequate epigenetic control of autophagy might thus restore tissue homeostasis and to limit fibrotic tissue remodeling.

## Results

### Autophagy is activated in patients with SSc and in experimental models of fibrosis

**Fig. 1:**
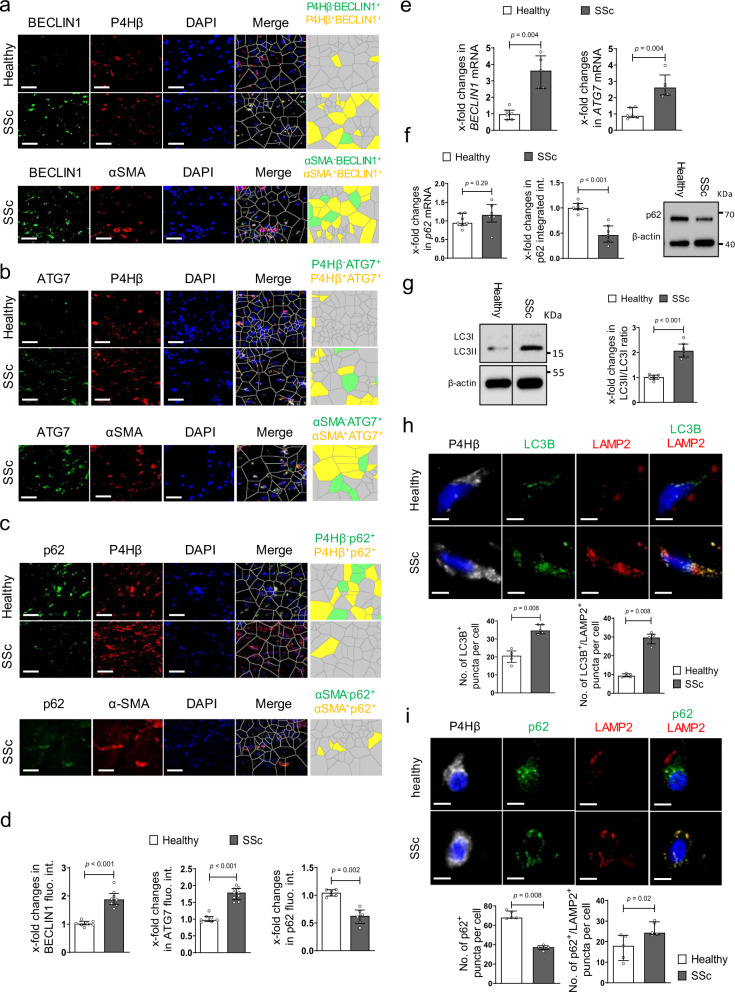
Autophagy is activated in fibrotic conditions. **a**–**d** SSc skin. **a**–**d** Representative immunofluorescence staining of BECLIN1 (**a**; *n* = 7 healthy individuals and *n* = 8 SSc patients), ATG7 (**b**; *n* = 7 healthy group and *n* = 9 for SSc group) or p62 (**c**; *n* = 6 patients per group) as markers of autophagy (all green) in combination with DAPI (blue) and the fibroblast marker P4Hβ or the myofibroblast marker αSMA (all red) with respective quantifications (**d**) and Voronoi mesh-based tessellated images. Horizontal scale bars, 50 µm. **e** mRNA levels of *BECLIN1* and *ATG7* (both with *n* = 6 patients for healthy and *n* = 5 for SSc). **f** mRNA (*n* = 7 biological replicates for healthy and *n* = 6 for SSc) and protein (*n* = 7 biological replicates per group) levels of p62 in cultured human skin fibroblasts. **g** Ratio of LC3 II to LC3 I in SSc fibroblasts and controls with representative western blots and quantification (*n* = 7 biological replicates per group). **h**–**i** SSc skin. **h** Co-staining of LC3B (green) and LAMP2 (red) in combination with DAPI (blue) and the fibroblast marker P4Hβ (gray) with representative confocal images and quantifications (*n* = 5 biological replicates per group). **i** Co-staining of p62 (green) and LAMP2 (red) in Combination with DAPI (blue) and the fibroblast marker P4Hβ (gray) (*n* = 5 biological replicates per group): Representative confocal images and quantifications. Horizontal scale bars, 5 µm. All data are presented as median ± IQR. *p*-values were determined by two-sided Mann–Whitney test and are indicated in the figure. See source data for more detailed information. Int.: intensity, fluo.: fluorescence., SSc: systemic sclerosis, P4Hβ: prolyl 4-hydroxylase. Western blot samples in panel **g** were run on the same gel. Images were cropped at the lines only for the purpose of this figure.

We first analyzed the activation levels of autophagy in SSc. We found that autophagy is upregulated in fibrotic skin of SSc patients with increased staining for BECLIN1 and ATG7 (Fig. 1a, b, d), two core regulators of autophagy, as compared to non-fibrotic skin. Co-staining with the fibroblast marker prolyl-4-hydroxylase-β (P4Hβ) and the myofibroblast marker α smooth muscle actin (αSMA) demonstrated that autophagy is particularly upregulated in SSc fibroblasts (Fig. 1a, b, d). Consistently, the protein levels of p62, which correlate inversely with the activation levels of autophagy^51^, were downregulated in the skin of SSc patients (Fig. 1c, d). Moreover, the mRNA levels of *BECLIN1* and *ATG7* were increased in fibrotic SSc skin as compared to control skin (Fig. 1e). Activation of autophagy with induction of BECLIN1 and ATG7 and downregulation of p62 in fibroblasts was also observed in murine models of pulmonary (Supplementary Fig. 1a–d) or dermal fibrosis (Supplementary Fig. 2a–d). The mRNA levels of *p62* were not significantly altered, demonstrating that the decrease in p62 protein is not due to impaired transcription, but rather due to degradation by increased autophagic flux (Fig. 1f). Similar findings were obtained in the mouse models of bleomycin-induced fibrosis and in TBRIact-induced fibrosis (Supplementary Figs. 1e and 3a). In addition, quantification of the levels of lipidated and unconjugated LC3B confirmed an increased autophagic flux in SSc fibroblasts (Fig. 1g). To further confirm the enhanced autophagic flux in SSc, we performed co-staining for LC3B with LAMP2 and p62 with LAMP2 and the fibroblast marker P4Hβ in skin sections with subsequent confocal microscopy and semi-automated quantification. LC3B-positive puncta were increased in fibroblasts in SSc skin compared to healthy individuals (Fig. 1h). We observed comparable increases of LC3B puncta in murine models of SSc (Supplementary Figs. 2e, f and 3b, c). Most of the LC3B-positive puncta co-localized with the lysosomal marker LAMP2. Moreover, p62 was decreased in fibrotic tissues and most p62-positive puncta co-localized with LAMP2 (Fig. 1i).

### TGFβ promotes autophagy in fibroblasts

**Fig. 2:**
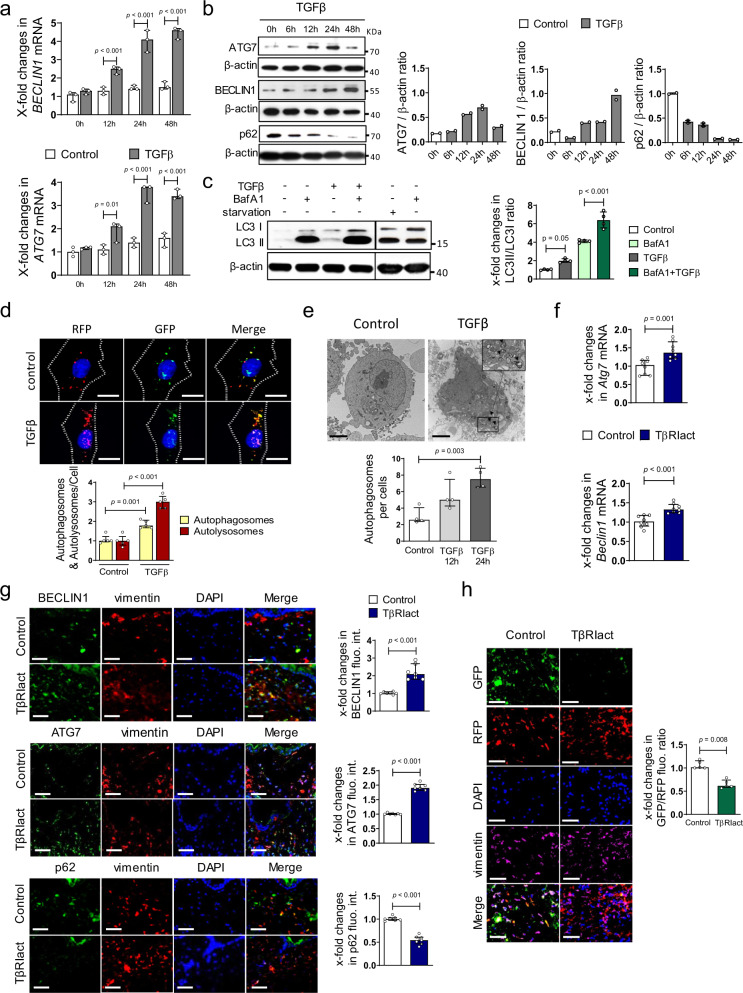
Autophagy is activated in a TGFβ-dependent manner in fibrosis. **a**–**e** Activation of autophagy in cultured fibroblasts by TGFβ. **a** mRNA levels of *BECLIN1* and *ATG7* (*n* = 3 samples per group). **b** Protein levels of ATG7, BECLIN1, and p62. Representative western blots as well as the quantifications (*n* = 2 biological replicates per group over two independent experiments). **c** Ratio of LC3 II to LC3 I: Representative western blots and quantification. Bafilomycin and starvation served as control (*n* = 4 samples per group). **d** EGFP/RFP-LC3 reporter activity; representative images and quantification (*n* = 5 biological replicates per group). Horizontal scale bar, 5 µm. **e** Representative electron microscopy images at 12,000-fold magnification (horizontal scale bars, 2 µm; *n* = 4 biological replicates) and quantification. Autophagosomes are indicated by arrows. **f**–**h** Overexpression of TβRIact in murine skin. **f** mRNA levels of *Beclin1* and *Atg7* (*n* = 8 biological replicates per group). **g** Representative immunofluorescence staining of BECLIN1, ATG7 or p62 (all green) in combination with DAPI (blue) and vimentin (red; *n* = 7 biological replicates per group for all experiments). **h** Representative images and quantification of EGFP/RFP-LC3 reporter activity in the skin (*n* = 5 biological replicates for both groups). Horizontal scale bars, 50 µm. All data are presented as median ± IQR. *p*-values were determined by ANOVA one-way with Sidak’s multiple comparisons test (**a**) or with Tukey’s multiple comparison post hoc test (**c**–**e**) and by two-sided Mann–Whitney test (**f**–**h**). *p*-values are indicated in the figure. See source data for more detailed information. Western blot samples in panel **c** cropped at the black horizontal line were not run on the same gel. Images were cropped at the line only for the purpose of this figure. int.: intensity, fluo.: fluorescence, TβRIact: constitutively active TGFβ receptor type I. BafA1: bafilomycin A1, fluo.: fluorescence.

The induction of autophagy in SSc as well as in experimental fibrosis models suggested that a common mediator of fibrosis might drive the activation of autophagy. Incubation with recombinant TGFβ increased the mRNA (Fig. 2a) and protein (Fig. 2b) levels of BECLIN1 and ATG7 and reduced the expression of p62 in cultured human fibroblasts. Stimulation of fibroblasts with TGFβ also promoted the conversion of LC3 I to LC3 II with increased ratios of LC3 II to LC3 I (Fig. 2c) and enhanced the intensity of acridine staining (Supplementary Fig. 4a). Starvation and incubation with bafilomycin A1 (BafA1) served as controls. Moreover, TGFβ reduced EGFP/RFP-LC3 reporter activity^52^ as additional readouts for activated autophagy (Fig. 2d). Furthermore, stimulation of fibroblasts with TGFβ promoted the formation of autophagosomes as demonstrated by electron microscopy (Fig. 2e). Immunostaining for LC3B in the presence and absence of BafA1 and TGFβ revealed that human fibroblasts exposed to TGFβ have increased levels of LC3B, which further increases in the presence of BafA1, suggesting increased autophagic flux after TGFβ stimulation compared to controls (Supplementary Fig. 4b).

The induction of autophagy by TGFβ was mediated by canonical TGFβ/SMAD3 signaling. siRNA-mediated knockdown of *SMAD3* prevented the TGFβ-induced upregulation of *ATG7* and *BECLIN1* mRNA and protein in fibroblasts (Supplementary Fig. 4c).

Moreover, activation of TGFβ signaling in vivo by overexpression of a constitutively active TGFβ receptor type I (TβRIact) in murine skin (Fig. 2f, g) or lungs (Supplementary Fig. 4d, e) activated autophagy with increased mRNA and protein levels of BECLIN1 and ATG7 and downregulated p62 expression. Overexpression of TβRIact also modulated EGFP/RFP-LC3 reporter activity in mice (Fig. 2h). In contrast, when TGFβ signaling was selectively inhibited in experimental fibrosis using the TβRI inhibitor SD-208, the induction of autophagy in bleomycin-induced fibrosis was abrogated and the levels of BECLIN1, ATG7 and p62 were comparable to those of non-fibrotic controls (Supplementary Fig. 5).

### TGFβ activates autophagy by repression of MYST1

Given the accumulating evidence for epigenetic alterations in the pathogenesis of fibrotic disease, we hypothesized that TGFβ might activate autophagy by an epigenetic mechanism. To evaluate the role of different epigenetic modifications, we selectively inhibited individual epigenetic modifications and analyzed the effects on the TGFβ-induced EGFP/RFP-LC3 reporter activity. Inhibition of histone deacetylases (HDACs) by trichostatin A (TSA) or by vorinostat (SAHA) abrogated TGFβ-induced changes in EGFP/RFP-LC3 reporter activity, whereas inhibition of DNA methyltransferases or of H3K27 histone methyltransferases had no significant effects on TGFβ-induced reporter activity (Supplementary Fig. 6a). The results indicate that histone acetylation plays a crucial role for the stimulatory effects of TGFβ on autophagy. To further investigate, how TGFβ alters histone acetylation to regulate autophagy, we first analyzed whether TGFβ induces the expression of HDACs in fibroblasts and whether the expression levels of HDACs differ between fibroblasts from SSc patients and healthy donors. Next, we examined potential effects of TGFβ on the expression of histone acetyltransferases. Given the plethora of enzymes with histone acetyltransferase activity, we first focused on the H4K16 histone acetyltransferase MYST1, which has been shown to be capable of modulating the transcription of autophagy-related genes^50,53^. We first confirmed that overexpression of MYST1 by adenoviral vectors induces H4K16 acetylation in cultured fibroblasts (Supplementary Fig. 6b).

Overexpression of MYST1 in fibroblasts abrogated the stimulatory effects of TGFβ on EGFP/RFP-LC3 reporter activity, whereas siRNA-mediated knockdown of *MYST1* enhanced the activity (Supplementary Fig. 6c).

**Fig. 3:**
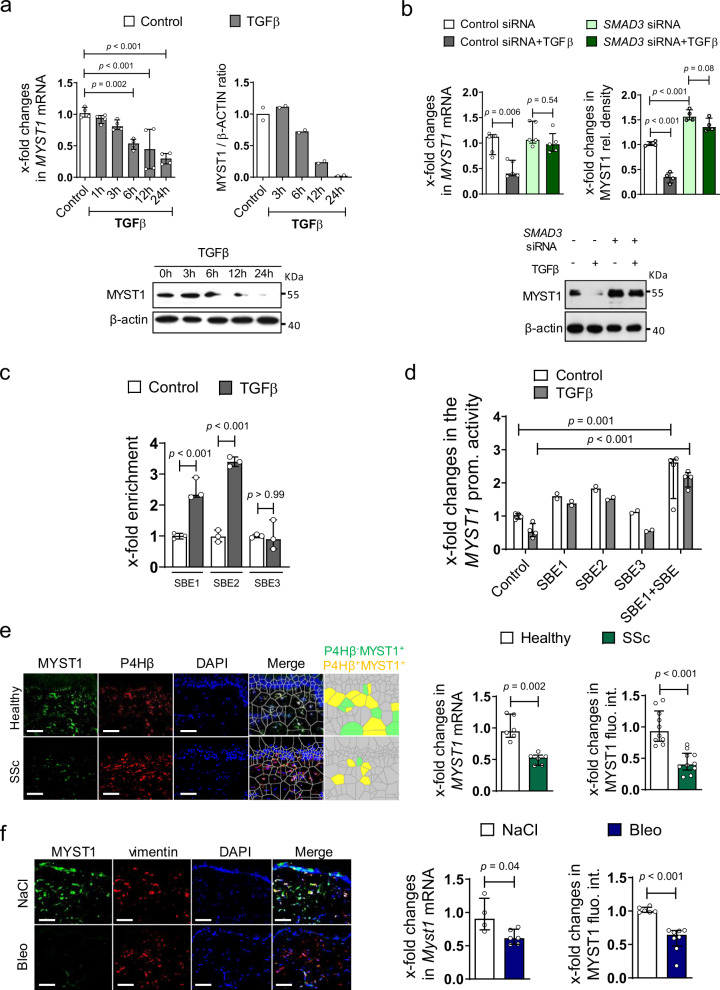
TGFβ downregulates MYST1 in fibroblasts. **a** mRNA (*n* = 4 biological replicates per group) and protein (*n* = 2 biological replicates per group over two independent experiments) levels of MYST1 in cultured fibroblasts stimulated with TGFβ. **b** Effects of siRNA-mediated knockdown of *SMAD3* on mRNA (*n* = 5 biological replicates per group) and protein (*n* = 4 biological replicates per group) levels of MYST1 in TGFβ-stimulated fibroblasts. **c** Chromatin immunoprecipitation assay for binding of SMAD3 at three putative SMAD binding Elements (SBE) in the *MYST1* promoter (*n* = 3 biological replicates). **d** Promoter assays using *MYST1* promoter constructs with selective mutations at three putative SBEs, (*n* = 2 biological replicates for SBE1, SBE2, and SBE3 groups and *n* = 4 for the other groups). **e** Expression of MYST1 in SSc patients and healthy individuals analyzed by qRT-PCR (*n* = 6 patients per group) and immunofluorescence staining with Voronoi mesh-based tessellated images (*n* = 10 patients per group). **f** Expression of MYST1 in fibrotic skin of mice challenged with bleomycin or non-fibrotic control mice as analyzed by qRT-PCR (*n* = 4 biological replicates for control group and *n* = 6 for bleomycin group) and immunofluorescence staining (*n* = 6 biological replicates for control group and *n* = 8 for bleomycin group). All horizontal scale bars, 50 µm. All data are presented as median ± IQR. *p*-values were determined by ANOVA one-way with Sidak’s multiple comparisons test (**a**, **c**) or with Tukey’s multiple comparison post hoc test (**b**, **d**) and by two-sided Mann–Whitney test (**e**, **f**). *p*-values are indicated in the figure. See source data for more detailed information. rel.: relative, P4Hβ: prolyl 4-hydroxylase, SSc: systemic sclerosis, Bleo: bleomycin, fluo.: fluorescence, int,: intensity, prom.: promoter, IP: immunoprecipitation.

We next analyzed whether TGFβ regulates MYST1 by modulating its expression. TGFβ downregulated the mRNA and protein levels of MYST1 in cultured human fibroblasts (Fig. 3a and Supplementary Fig. 7a). TGFβ signaling also regulated the expression of MYST1 in vivo with decreased levels of MYST1 in murine lungs (Supplementary Fig. 7b) and skin (Supplementary Fig. 7c) upon forced expression of TβRIact.

The repressive effects of TGFβ were dependent on canonical SMAD signaling. In silico analysis of the human *MYST1* promoter revealed three SMAD binding elements (SBE). siRNA-mediated knockdown of *SMAD3* (Fig. 3b) or incubation with the SMAD3 inhibitor SIS3 (Supplementary Fig. 7d, e) abrogated the repressive effects of TGFβ on MYST1. Individual analyses of the three predicted SBEs by ChIP assays (Fig. 3c) revealed that TGFβ strongly induced binding of SMAD3 to two SBE at residues 279-282 and at residues 384-387 (referred to SBE1 and SBE2, respectively) of *MYST1* promoter sequence, but had only minor effects on the predicted binding site at residues 574-577 (referred to as SBE3) (Fig. 3c). Consistently, mutations of the SBE1 and the SBE2, but not at the SBE3, prevented the inhibitory effects of TGFβ in *MYST1* reporter assays (Fig. 3d).

To provide evidence that this epigenetic regulation is operative in fibrotic conditions, we assessed the expression of MYST1 in skin sections of SSc patients. The mRNA and protein levels of MYST1 were decreased in SSc skin compared to non-fibrotic skin from healthy donors (Fig. 3e). Reduced MYST1 expression was also observed in bleomycin-induced dermal (Fig. 3f) and pulmonary (Supplementary Fig. 7f, g) fibrosis.

**Fig. 4:**
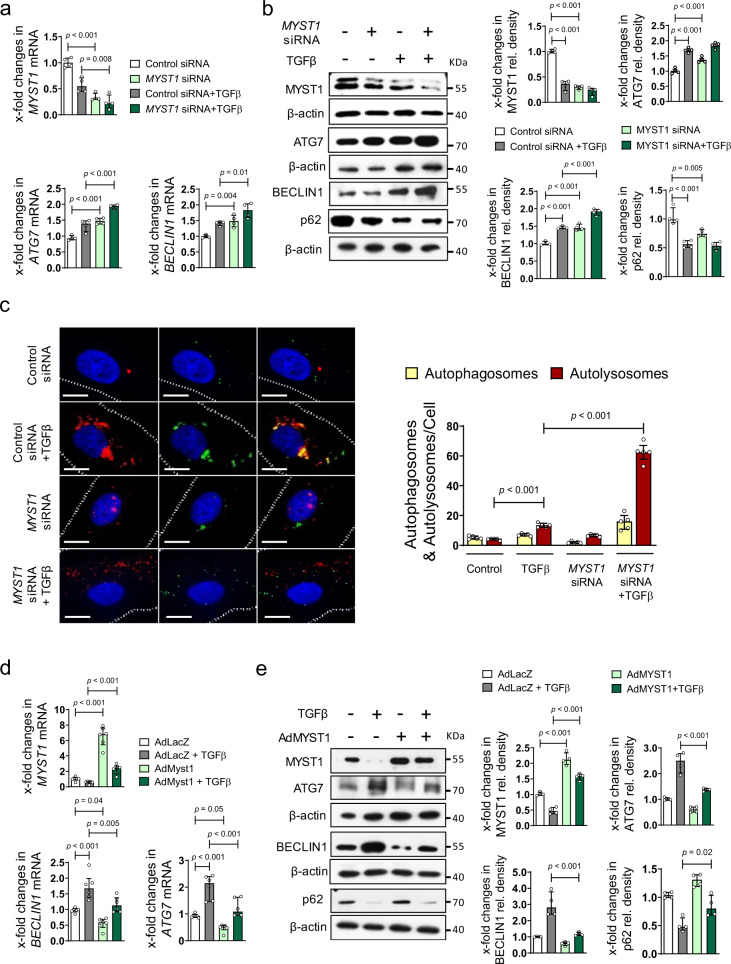
Downregulation of MYST1 mediates the stimulatory effects of TGFβ on autophagy. **a**, **b** Effects of siRNA-mediated knockdown of MYST1 on autophagy in healthy human dermal fibroblasts as assessed by quantification of the mRNA levels of *ATG7* (*n* = 4 biological replicates per group and *n* = 3 for MYST1 siRNA + TGFβ), *BECLIN1* (*n* = 4 biological replicates per group) and respective protein quantifications (*n* = 4 biological replicates per group). Confirmation of reduced mRNA (*n* = 4 biological replicates per group) (**a**) and protein (**b**; *n* = 4 biological replicates per group) levels of MYST1 by qRT-PCR and western blot. **c** EGFP/RFP-LC3 reporter activity upon knockdown of MYST1; representative images and quantification (*n* = 5 biological replicates per group). Horizontal scale bar, 5 µm. **d**, **e** mRNA expression of *ATG7*, *BECLIN1*, and *MYST1* (*n* = 6 biological replicates per group) (**d**) and respective protein quantification (**e**) (*n* = 4 biological replicates per group) levels after adenoviral overexpression of MYST1 in healthy human dermal fibroblasts (**e**). All data are presented as mean ± IQR. *p*-values were determined by ANOVA one-way with Tukey’s multiple comparison post hoc test. See source data for more detailed information. rel.: relative, Ad: adenovirus. The results in panel c are part of a large set of experiments and share controls with Supplementary Fig. 8a. Data are presented separately just for the purpose of this figure.

To demonstrate that downregulation of *MYST1* is sufficient to activate autophagy in human fibroblasts, *MYST1* expression was targeted by siRNA. Downregulation of *MYST1* expression, to levels comparable to those in TGFβ-stimulated human fibroblasts, activated autophagy with increased levels of *ATG7* and *BECLIN1* mRNA and protein, downregulation of p62 (Fig. 4a, b) and reduced EGFP/RFP-LC3 reporter activity (Fig. 4c). In contrast, overexpression of MYST1 in human fibroblasts abrogated the stimulatory effects of TGFβ on autophagy (Fig. 4d, e). To confirm that the antifibrotic effects of MYST1 are mediated via autophagy, we overexpressed BECLIN1 and simultaneously knocked down *MYST1* in fibroblasts. Knockdown of *MYST1* further amplified the stimulatory effects of BECLIN1 overexpression on fibroblasts with higher levels of *COL1A1* mRNA and type I collagen protein (Supplementary Fig. 7h).

### Activation of autophagy stimulates fibroblast activation and induces fibrosis

**Fig. 5:**
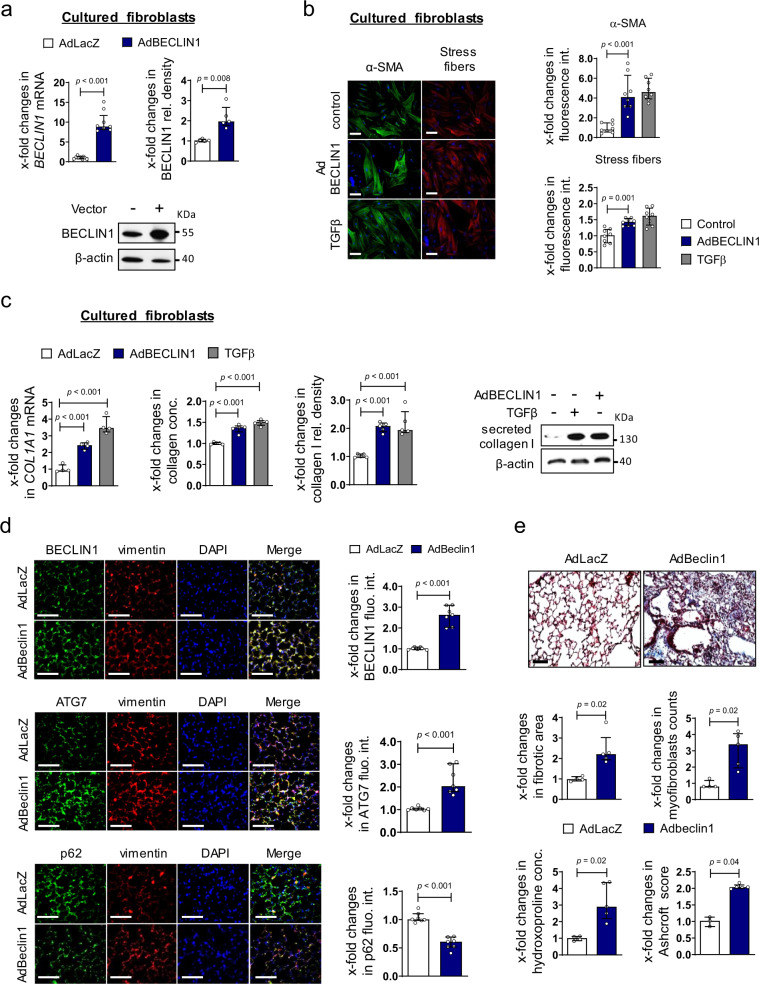
Autophagy induces fibroblast-to-myofibroblast transition, collagen release, and tissue fibrosis. **a**–**c** Cultured human dermal fibroblasts overexpressing BECLIN1**. a** Expression of BECLIN1 mRNA (*n* = 9 biological replicates per group) and protein (*n* = 5 biological replicates per group) in fibroblasts infected with adenoviral vectors encoding *BECLIN1* or *LacZ*. **b** Effects of the adenoviral overexpression of BECLIN1 on the expression of αSMA and the formation of stress fibers (*n* = 8 biological per group) with representative images and quantification of fluorescence intensity. Fibroblasts stimulated with recombinant TGFβ served as positive control. Horizontal scale bar, 100 µm. **c** Changes in the mRNA levels of *COL1A1* as analyzed by qRT-PCR (*n* = 4 biological replicates per group), on type I collagen protein as analyzed by western blot (*n* = 5 biological replicates per group) and on total collagen analyzed by hydroxyproline assays in fibroblasts upon adenoviral overexpression of BECLIN1 (*n* = 5 biological replicates per group). **d**, **e** Adenoviral overexpression of BECLIN1 in murine lungs. **d** Representative immunofluorescence staining of the markers of autophagy BECLIN1, ATG7, or p62 (all green) in combination with DAPI (blue) and vimentin (red) (*n* = 7 biological replicates per group). **e** Trichrome stainings, fibrotic area counts, myofibroblast counts, hydroxyproline content (*n* = 4 biological replicates for control group and *n* = 5 for AdBeclin1 group) and Ashcroft scores (*n* = 3 biological replicates for control group and *n* = 5 for AdBeclin1 group). Horizontal scale bars, 100 µm. All data are presented as median ± IQR. *p*-values were determined by two-sided Mann–Whitney test (**a**, **d**, **e**) or ANOVA one-way with Tukey’s multiple comparison post hoc test (**b**, **c**) and are indicated in the figure. See source data for more detailed information. rel.: relative, Ad: adenovirus, fluo.: fluorescence, int.: intensity, conc.: concentration.

We next aimed to determine whether upregulation of autophagy may contribute to the activated phenotype of SSc fibroblasts and promote collagen release. Overexpression of BECLIN1 induced autophagy (Fig. 5a and Supplementary Fig. 8a) and promoted the differentiation of resting fibroblasts into myofibroblasts with increased expression of α-smooth muscle actin (αSMA) and formation of stress fibers (Fig. 5b). Overexpression of BECLIN1 also increased the mRNA levels of *COL1A1* and stimulated the release of type I collagen protein and total collagen protein into the supernatant (Fig. 5c). In contrast, co-incubation of TGFβ stimulated human dermal fibroblasts with chloroquine (CQ), a pharmacological inhibitor of autophagy, decreased the mRNA levels of *COL1A1* and reduced the secretion of collagen protein (Supplementary Fig. 8b).

Overexpression of BECLIN1 also activated autophagy in vivo and induced fibrosis in the skin and lungs of mice in the absence of additional profibrotic stimuli. Overexpression of BECLIN1 in the lungs of mice activated autophagy (Fig. 5d) and induced myofibroblast differentiation with accumulation of collagen and pulmonary fibrosis (Fig. 5e). Overexpression of BECLIN1 was also sufficient to induce autophagy, myofibroblast differentiation and fibrosis in the skin of mice (Supplementary Fig. 8c, d). To further validate that autophagy regulates the deposition of extracellular matrix, we quantified extracellular matrix (ECM) deposition in fibroblasts overexpressing BECLIN1 and in fibroblasts treated with the autophagy inhibitor 3-methyladenine (3-MA) (Supplementary Fig. 9a, b). Overexpression of BECLIN1 promoted deposition of collagen I, collagen III and fibronectin, with effects comparable to that of TGFβ (Supplementary Fig. 9a).

Recent evidence demonstrated that the autophagic machinery is required for secretion of TGFβ1 and that inhibition of autophagy blocks the release of TGFβ1^26^. We therefore investigated whether MYST1 may ameliorate fibroblast activation and tissue fibrosis by inhibition of TGFβ signaling. Overexpression of BECLIN1 induced the mRNA levels of the prototypical TGFβ/SMAD target genes *PAI-1* and *CTGF* in human dermal fibroblasts (Supplementary Fig. 10a). In contrast, inhibition of autophagy by overexpression of MYST1 reduced the stimulatory effects of TGFβ on *PAI-1* and *CTGF* mRNA (Supplementary Fig. 10b). Consistently, overexpression of BECLIN1 increased the levels of active TGFβ released from dermal fibroblasts, whereas overexpression of MYST1 decreased them (Supplementary Fig. 10c). Overexpression of BECLIN1 also promoted TGFβ signaling in murine skin with increased levels of pSMAD3 and of active TGFβ in western blot and in MLC assays (Supplementary Fig. 10d, e). Overexpression of MYST1 ameliorated TGFβ signaling with reduced levels of pSMAD3 and of active TGFβ in bleomycin-challenged mice (Supplementary Fig. 10f, g).

### Inhibition of autophagy by fibroblast-specific knockout of *Atg7* reduces fibroblast activation and ameliorates experimental fibrosis

**Fig. 6:**
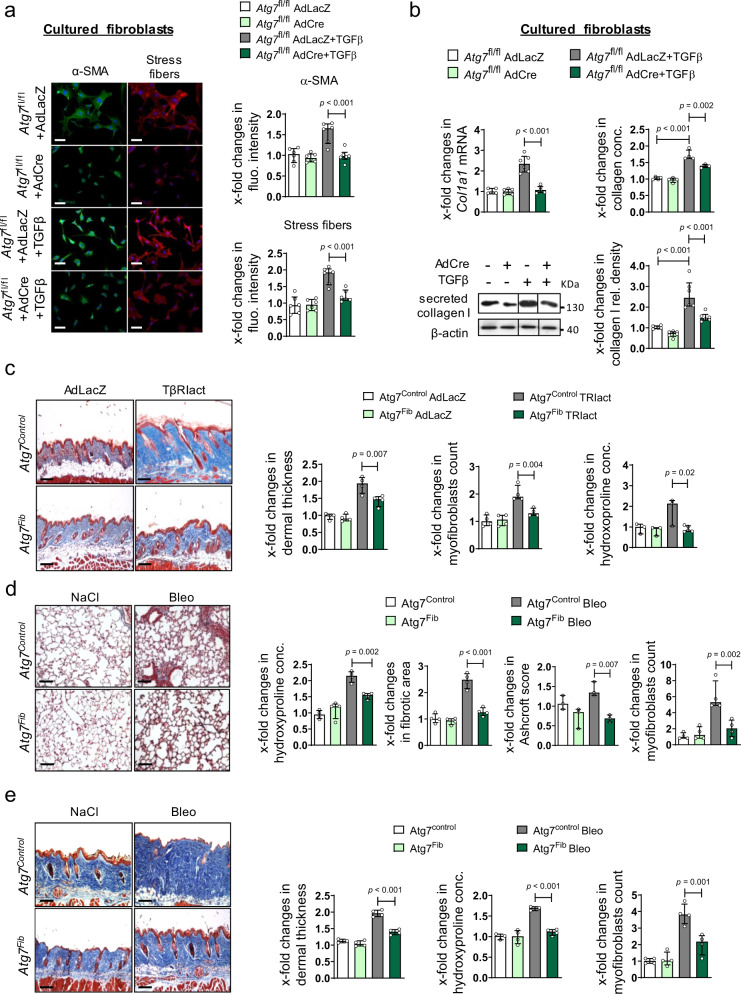
Knockout of Atg7 ameliorates fibroblast activation and experimental dermal and pulmonary fibrosis. **a**, **b** Cultured murine *Atg7* knockout fibroblasts. **a** Expression of αSMA and formation of stress fibers. Representative images and quantification (*n* = 6 biological replicates per group). **b** mRNA levels of *Co1la1* analyzed by qRT-PCR (*n* = 6 biological replicates per group), type I collagen protein as analyzed by western blot (*n* = 6 biological replicates per group) and total collagen analyzed by hydroxyproline assays (*n* = 4 biological replicates per group). Horizontal scale bar, 100 µm. **c**–**e** Mice with fibroblast-specific knockout of *Atg7*. **c** TβRIact-induced skin fibrosis: Representative trichrome stainings, dermal thickening, myofibroblast counts (*n* = 4 biological replicates per group) and hydroxyproline content (*n* = 5 biological replicates for Atg7^fib^ TβRIact group, *n* = 3 other groups). **d** Experimental pulmonary fibrosis induced by intratracheal instillation of bleomycin: Representative trichrome stainings, hydroxyproline content, fibrotic area (*n* = 4 biological replicates per group), Ashcroft scores (*n* = 3 biological replicates per groups) and myofibroblast counts (*n* = 4 biological replicates per group). **e** Experimental dermal fibrosis induced by subcutaneous injections of bleomycin: Representative trichrome stainings, dermal thickening (*n* = 3 biological replicates for Atg7^control^ and *n* = 4 for other groups) myofibroblast counts (*n* = 4 biological replicates per group) and hydroxyproline content (*n* = 3 biological replicates for Atg7^control^ and Atg7^control^ Bleo groups and *n* = 4 for other groups). Horizontal scale bars, 100 µm. Western blot samples in panel **b** were run on the same gel. Images were cropped at the lines only for the purpose of this figure. All data are presented as median ± IQR. *p*-values were determined by ANOVA one-way with Tukey’s multiple comparison post hoc test and are indicated in the figure. See source data for more detailed information. rel.: relative, Ad: adenovirus, fluo.: fluorescence, conc.: concentration. TβRIact: constitutively active TGFβ receptor type I, Bleo: bleomycin, *Atg7*^control^: littermate control mice *Atg7*^fib^: fibroblast-specific *Atg7* knockout mice.

We next investigated whether inactivation of autophagy can inhibit TGFβ-induced fibroblast-to-myofibroblast differentiation. Indeed, fibroblasts lacking *Atg7* were less sensitive to the profibrotic effects of TGFβ with decreased expression of αSMA and stress fibers (Fig. 6a) and reduced release of collagen protein as compared to control fibroblasts (Fig. 6b). Moreover, we analyzed the levels of type I collagen released by human fibroblasts with stable knockout of *ATG7* using SILAC-based quantitative proteomics (Supplementary Fig. 11a). Inhibition of autophagy by knockout of *ATG7* ameliorates the release of both type I collagen chains in the supernatant. The differences in type I collagen release were confirmed by western blot (Supplementary Fig. 11b). We also observed impaired TGFβ release and activation upon *ATG7* knockout by SILAC-based proteomics (Supplementary Fig. 11a) and in cells expressing FLAG-tagged TGFβ (Supplementary Fig. 11c).

In addition, inhibition of autophagy by 3-MA inhibited TGFβ-induced myofibroblast differentiation as well as TGFβ-induced deposition of type I and type III collagen and of fibronectin (Supplementary Fig. 9b and 12a).

We next analyzed whether inactivation of autophagy can ameliorate experimental fibrosis. We first demonstrated that treatment of mice with the autophagy inhibitor 3-MA can ameliorate bleomycin-induced skin fibrosis (Supplementary Fig. 12b).

We next generated *Atg7*^fl/fl^ × *Col1a2*;*Cre*ER mice and *Atg7*^fl/fl^ × *Col6*;*Cre* for selective inactivation of *Atg7* in fibroblasts^8,54^. Inactivation of autophagy in fibroblasts ameliorated TGFβ-dependent experimental pulmonary and dermal fibrosis. Fibroblast-specific knockout of *Atg7* reduced the TβRIact-induced increase in Ashcroft scores and in fibrotic area as well as myofibroblast counts and hydroxyproline content compared to mice with normal expression of *Atg7* in experimental pulmonary fibrosis (Supplementary Fig. 13). Inactivation of *Atg7* also prevented TβRIact-induced skin fibrosis, demonstrating that the activation of autophagy directly contributes to the profibrotic effects of TGFβ (Fig. 6c). In addition to TβRIact-induced fibrosis, mice with fibroblast-specific knockout of *Atg7* were also less sensitive to the profibrotic effects of intratracheal or subcutaneous  application of bleomycin with less fibrotic changes on histology, reduced myofibroblast counts and lower hydroxyproline content (Fig. 6d, e).

**Fig. 7:**
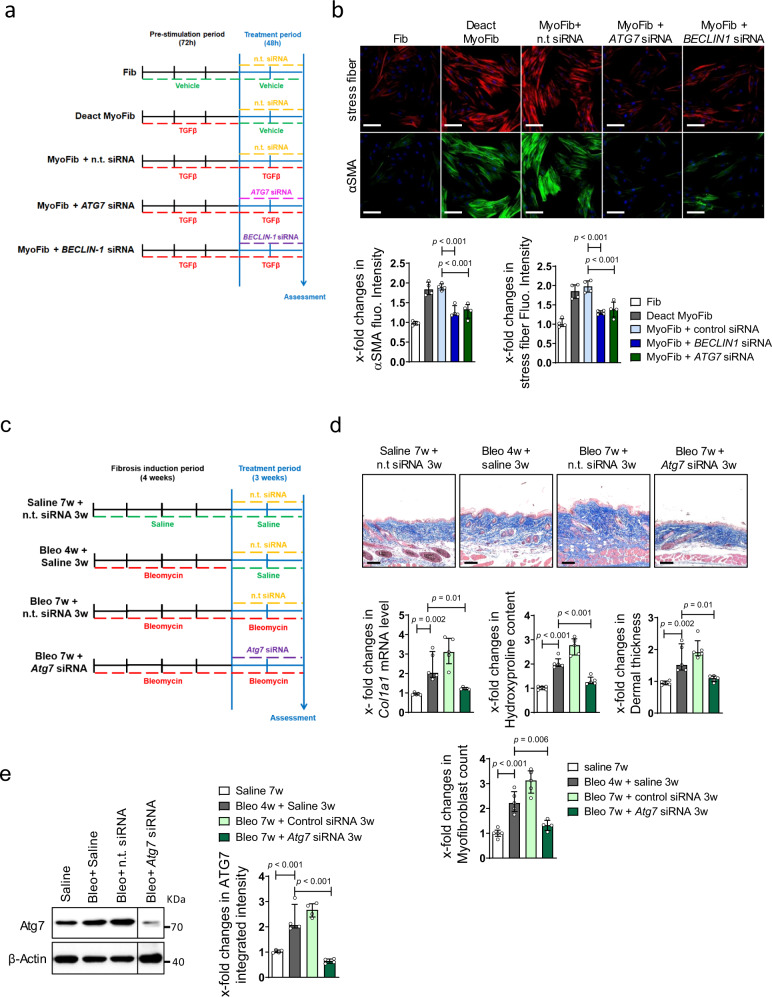
Inhibition of autophagy deactivates myofibroblasts and induces regression of fibrosis. **a**, **b** Knockdown of *ATG7* and *BECLIN-1* in human fibroblasts. **a** Schematic illustration of the experimental procedure. **b** Expression of αSMA (green) and formation of stress fibers (red), costained with DAPI (blue). Representative images and quantification of αSMA and Stress fibers fluorescence intensity (*n* = 4 biological replicates per group). Horizontal scale bar, 50 µm. **c**, **d** Effects of *Atg7* knockdown in a model of established bleomycin-induced skin fibrosis. **c** Schematic illustration of the experimental procedure. **d** Representative trichrome stainings, *Col1a1* mRNA, hydroxyproline content, dermal thickness (*n* = 5 biological replicates per group) and myofibroblast counts (*n* = 4 biological replicates for Bleo 7w + *Atg7* siRNA 3w group and n = 5 for other groups). **e** Representative western blot and quantification of ATG7 protein levels (*n* = 4 biological replicates per group). Horizontal scale bar, 100 µm. All data are presented as median ± IQR. *p*-values were determined by ANOVA one-way with Tukey’s multiple comparison post hoc test and are indicated in the figure. See source data for more detailed information. Fluo.: fluorescence, Bleo: bleomycin. Fib: fibroblasts, Deact MyoFib: deactivated myofibroblasts, w: weeks. Western blot samples in panel **d** were run on the same gel. Images were cropped at the lines only for the purpose of this figure.

We next investigated whether inhibition of autophagy can deactivate myofibroblasts and induce regression of tissue fibrosis. Knockdown of *ATG7* or *BECLIN-1* in human fibroblasts reduced the expression of αSMA and the number of stress fibers in myofibroblasts, indicating re-differentiation of myofibroblasts into resting fibroblasts upon inhibition of autophagy (Fig. 7a, b,). Similar results were obtained with the autophagy inhibitors CQ and 3-MA (Supplementary Fig. 14a, b).

Moreover, we evaluated the effects of autophagy inhibition in a model of established bleomycin-induced skin fibrosis (Fig. 7c–e). In this model, fibrosis was induced by injections of bleomycin for four weeks. After these four weeks, we inhibited autophagy by siRNA-mediated knockdown of *Atg7*, while continuing to challenge the mice with bleomycin for additional three weeks. Challenge of mice with bleomycin for four weeks induced robust fibrosis, with further progression of skin fibrosis in mice challenged for additional three weeks (Fig. 7c). siRNA-mediated knockdown of *Atg7* effectively prevented progression of fibrosis with reduced dermal thickness, myofibroblast counts and hydroxyproline content (Fig. 7d). Of note, inactivation of autophagy also decreased dermal thickness, myofibroblast counts and hydroxyproline content to below pretreatment levels, indicating regression of bleomycin-induced skin fibrosis.

### Overexpression of MYST1 ameliorates experimental fibrosis

**Fig. 8:**
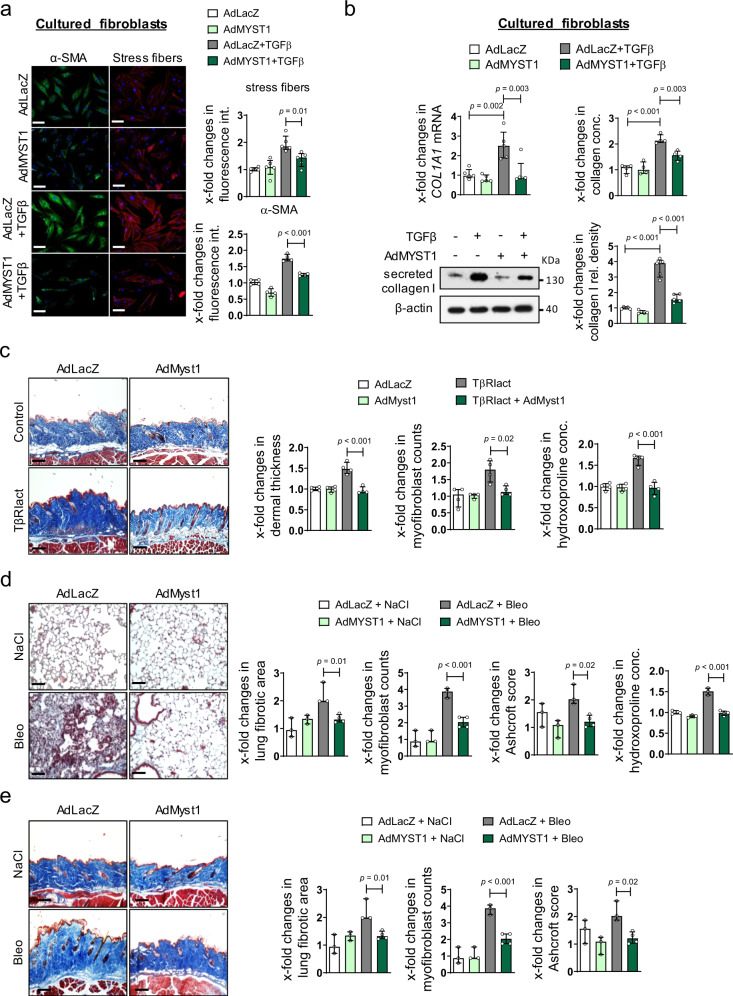
Re-expression of MYST1 inhibits TGFβ-dependent fibroblast activation and prevents experimental fibrosis. **a**, **b** Overexpression of MYST1 in cultured human dermal fibroblasts: **a** Expression of αSMA (*n* = 4 biological replicates per group) and formation of stress fibers (*n* = 5 biological replicates per group). Representative images and quantification. Horizontal scale bar, 100 µm. **b** mRNA levels of *COL1A1* analyzed by qRT-PCR (*n* = 5 biological replicates per group), total collagen as analyzed by hydroxyproline assays (*n* = 4 biological replicates per group) and type I collagen protein as analyzed by western blot (*n* = 5 biological replicates per group). **c**–**e** Adenoviral overexpression of MYST1 in murine models. **c** TβRIact-induced skin fibrosis: Representative trichrome stainings for, dermal thickening, myofibroblast counts and hydroxyproline content (*n* = 4 biological replicates per group). **d** Bleomycin-induced pulmonary fibrosis: Representative Trichrome stainings, fibrotic area, myofibroblast counts, Ashcroft scores and hydroxyproline content (*n* = 4 biological replicates for the group AdMyst1 treated with TGFβ and *n* = 3 for other groups). **e** Bleomycin-induced dermal fibrosis: Representative trichrome stainings shown, dermal thickening, myofibroblast counts and hydroxyproline content (*n* = 4 biological replicates for bleomycin treated groups and *n* = 3 for saline treated groups). Horizontal scale bars, 100 µm. All data are presented as median ± IQR. *p*-values were determined by ANOVA one-way with Tukey’s multiple comparison post hoc test and are indicated in the figure. See source data for more detailed information. rel.: relative, Ad: adenovirus, conc.: concentration, int.: intensity, TβRIact: constitutively active TGFβ receptor type I, Bleo: bleomycin.

Based on the potent inhibitory effects of MYST1 on autophagy and its strongly decreased expression in SSc, we hypothesized that forced expression of MYST1 may exert antifibrotic effects by preventing TGFβ-induced activation of autophagy. To test this hypothesis, we first assessed the effects of MYST1 overexpression on the TGFβ-dependent activation of human dermal fibroblasts. Overexpression of MYST1 reduced the TGFβ-induced fibroblast-to-myofibroblast transition with decreased expression of αSMA and impaired formation of stress fibers (Fig. 8a). Consistently, the levels of *COL1A1* mRNA and collagen protein were reduced in fibroblasts overexpressing MYST1 compared to control cells (Fig. 8b).

Overexpression of MYST1 also ablated the activation of autophagy in TβRIact-induced skin fibrosis. TβRIact-induced changes in expression levels of ATG7, BECLIN1 and p62 were prevented in MYST1-overexpressing mice and the levels of these markers of autophagy were comparable to those of non-fibrotic control mice (Supplementary Figs. 15a, b and 16a). The inhibitory effects of MYST1 on autophagy translated into potent antifibrotic effects. Adenoviral overexpression of MYST1 ameliorated TβRIact-induced skin fibrosis with reduced dermal thickening, lower myofibroblast counts and decreased hydroxyproline content as compared to TβRIact-challenged mice infected with control adenoviruses encoding *LacZ* (Fig. 8c). Overexpression of MYST1 also prevented activation of autophagy in the lungs and skin of bleomycin-challenged mice (Supplementary Figs. 16b, c, 17a, b, and 18a, b). Bleomycin-induced fibrosis was significantly less severe in both organs of mice infected with adenoviruses encoding *Myst1* as compared to controls (Fig. 8d, e).

To exclude that the inhibitory effects of MYST1 on fibroblast activation and tissue fibrosis are mediated by apoptosis, we first measured the activity of Caspase 3 and 7 in fibroblasts with siRNA-mediated knockdown of *MYST1* or with adenoviral overexpression of MYST1. Neither knockdown nor overexpression of MYST1 altered Caspase activity in human dermal fibroblasts as compared to respective control cells (Supplementary Fig. 19a, b). We also assessed whether overexpression of MYST1 alters the levels of apoptosis in fibrotic murine tissues. The number of apoptotic cells, in particular of fibroblasts, was not altered in bleomycin- or TβRIact-challenged mice as analyzed by TUNEL staining (Supplementary Fig. 19c–e).

## Discussion

In the present study, we demonstrate that autophagy is strongly activated in fibroblasts from SSc skin and also in experimental dermal and pulmonary fibrosis as compared to respective non-fibrotic control tissue with increased expression levels of ATG7 and BECLIN1, decreased expression of p62 and enhanced activity in in vivo autophagy reporter studies. The aberrant activation of autophagy had profound stimulatory effects on fibroblasts. Activation of autophagy by forced expression of BECLIN1 promoted fibroblast-to-myofibroblast transition, induced activation of latent TGFβ and stimulated the collagen release in vitro and induced dermal and pulmonary fibrosis in vivo. Moreover, inactivation of autophagy by fibroblast-specific knockout of *Atg7* prevented myofibroblast differentiation and ameliorated pulmonary and dermal fibrosis, demonstrating that activation of autophagy is both, sufficient and required, for fibroblast activation in fibrosis.

Recent studies demonstrate that autophagy is activated in several fibrotic diseases such as liver fibrosis^37,55,56^, renal interstitial fibrosis^39^, cardiac fibrosis^42^ and fibrosis of the peritoneum^45^. However, other reports point to a downregulation in idiopathic pulmonary fibrosis (IPF) at tissue level and murine models of pulmonary fibrosis^57–64^ In contrast to those findings, autophagy was reported to be activated in cultured human fibroblasts derived from patients with IPF^37^. Moreover, autophagy was reported to be activated in a murine model of amiodarone-induced pulmonary fibrosis^65^. Together these findings suggest a cell- and context-specific regulation of autophagy.

We present several lines of evidence that impaired epigenetic control is a central mechanism for the aberrant activation of autophagy in fibrotic tissue remodeling. Screening of different epigenetic modifiers identified the H4K16 histone acetyltransferase MYST1 as the central epigenetic regulator of TGFβ-induced autophagy, whereas selective inhibition of several other epigenetic modifications relevant to the pathogenesis of fibrotic diseases such DNA methyltransferases or of H3K27 histone methyltransferases^10,14,16,66–68^ had no effect. Our study is the first to link deregulation of MYST1 to the pathogenesis of fibrotic diseases. This finding supports the concept that epigenetic alterations play a critical role in the persistent activation of fibroblasts in fibrotic diseases.

MYST1 regulates autophagy by H4K16 acetylation at the promoters of autophagy-related genes such as p62/SQSTM1^49^, which has previously been demonstrated to be associated with reduced transcription of target genes^48^. Under physiologic conditions, appropriate expression of MYST1 prevents uncontrolled activation of autophagy by inhibiting the expression of core components of autophagy such as ATG7 and BECLIN1. In patients with SSc as well as in experimental fibrosis, however, the expression of MYST1 is strongly downregulated, thus impairing the epigenetic control of autophagy. We demonstrate that the downregulation of MYST1 alone is sufficient to stimulate the expression of ATG7 and BECLIN1 and to promote activation of autophagy in cultured fibroblasts and in experimental fibrosis. Our findings are in agreement with a study by Hale et al.^50^, which identified MYST1 as a modulator of autophagy in an siRNA-based screening approach.

In contrast, overexpression of MYST1 prevented the aberrant activation of autophagy, inhibited TGFβ-induced fibroblast activation and ameliorated experimental dermal and pulmonary fibrosis in complementary murine models. Impaired control of autophagy with downregulation of MYST1 may thus play a central role in the aberrant fibroblast activation and might be a potential target for therapeutic intervention in fibrotic diseases.

We demonstrate in multiple experiments that canonical TGFβ signaling is a central regulator of MYST1 expression and thus an important upstream regulator of autophagy in SSc: (I) Incubation with recombinant TGFβ decreased the mRNA and protein levels of MYST1 in cultured fibroblasts and induced autophagy. (II) Overexpression of TβRIact downregulated the expression of MYST1 in murine skin and lungs. (III) The inhibitory effects of TGFβ were mediated by canonical TGFβ/SMAD signaling and knockdown of *SMAD3* abrogated the downregulation of MYST1. (IV) Treatment with SD-208, a selective inhibitor of TGFβ receptor type 1^69^, prevented the downregulation of MYST1 and the subsequent activation of autophagy in experimental fibrosis. Although factors such as reduced supply of nutrients and hypoxia^70^ due to capillary rarefication likely contribute to the enhanced activation of autophagy in fibrotic diseases^71^, these findings characterize TGFβ as a central upstream regulator of autophagy in the context of fibrosis. Given the potent profibrotic effects of enhanced autophagy in fibroblasts, our study unravels the activation of autophagy as a mechanism by which TGFβ orchestrates fibroblast activation and tissue fibrosis.

Our study demonstrates that activation of autophagy promotes fibroblast-to-myofibroblast transition and that selective inactivation of autophagy ameliorates TβRIact- and bleomycin-induced dermal and pulmonary fibrosis. However, general targeting of autophagy for the treatment of fibrosis by systemic application of inhibitors might be associated with adverse events. The major physiological role of autophagy is to provide nutrients under conditions of cellular stress and prevent cells from undergoing apoptosis. The homeostatic effects of autophagy may be of particular relevance in SSc. Apoptosis of microvascular endothelial cells with consecutive capillary rarefication, reduced perfusion and tissue hypoxia are key features of SSc. Under those conditions, direct inhibition of autophagy might further increase apoptosis of microvascular endothelial cells, aggravate microvascular disease and induce vascular complications. In contrast to approaches that interfere with autophagy directly, modulation of H4K16 acetylation via MYST1 would rather modulate the threshold for autophagy. This approach might allow maintaining the levels of autophagy that are required for homeostatic functions. Although forced re-expression of MYST1 in skin and lung was not accompanied by adverse events in our models, further studies in murine models with more extensive vascular involvement and in other organs are required to exclude vascular complications and to confirm the tolerability of targeting autophagy via MYST1.

In summary, we demonstrate that the epigenetic control of autophagy is disturbed by a TGFβ-dependent downregulation of MYST1 in SSc as a typical idiopathic fibrotic disease. The increased activation of autophagy induces fibroblast-to-myofibroblast transition and promotes fibrotic tissue remodeling. Re-expression of MYST1 prevents aberrant autophagy, limits the profibrotic effects of TGFβ and ameliorates experimental fibrosis. Restoration of the epigenetic control of autophagy might thus ameliorate fibrotic tissue remodeling.

## Methods

### Patients

Dermal fibroblasts were isolated from skin biopsies of 23 SSc patients and 12 age- and sex-matched healthy volunteers. All patients fulfilled the ACR/EULAR criteria for SSc^72^. Seventeen patients were female, six were male. The median age of SSc patients was 44 years (range: 19–61 years), and their median disease duration was 4 years (range: 0.5–8 years). The median age was 51 years (range 40–68 years).

### Cell culture

Human dermal fibroblasts were isolated from twenty-three SSc patients and twelve age- and sex-matched healthy volunteers. Murine fibroblasts were isolated from skin biopsies of mice expressing two conditional alleles of *Atg7* (*Atg7*^fl/fl^) mice (kindly provided by Dr. Masaaki Komatsu, Tokyo Metropolitan Institute of Medical Science, Tokyo, Japan^54^). To delete *Atg7*, fibroblasts were infected with replication-incompetent adenovirus type 5 (Ad5) encoding for *Cre* (AdCre) recombinase. Human and murine fibroblasts were used in passages 4–8 for all experiments. Two clones of Wi-26/SV-40 human lung fibroblasts with stable knockout of ATG7 (clones three and seven) were used in experiments to detect intracellular and secreted levels of type I collagen and TGFβ1. In selected experiments, fibroblasts were stimulated with recombinant TGFβ (10 ng/ml) (PeproTech, Hamburg, Germany), incubated with the Smad3 inhibitor SIS3 (1 μM) (Sigma-Aldrich, Munich, Germany) and the autophagy inhibitor Chloroquine (10 μM) (Biotrend, Cologne, Germany), 3MA (5 mM) (Sigma-Aldrich) and Bafilomycin A1 (20 nM) (Sigma-Aldrich) for further analyses.

For viral infection experiments 80 ifu/cell of Ad5 were used. Ad5 encoding *LacZ* served as controls. Plasmid constructs were transfected into primary fibroblasts using the 4D-Nucleofector (Lonza, Cologne, Germany)^73^. The transfection efficiency was determined by co-transfection with pSv-β-galactosidase vectors (Promega, Mannheim, Germany). Gene silencing was achieved by transfection of 3 μg pre-designed siRNA duplexes against *SMAD3* and *MYST1* (Abgent, San Diego, CA, USA) using the 4D-Nucleofector. Non-targeting siRNAs (nt siRNA) (Life Technologies, Darmstadt, Germany) served as controls.

### Generation of *ATG7* knockout cells using CRISPR/Cas9

 Human embryonic lung fibroblasts WI26/SV-40 were used to generate stable ATG7 knock-out clones. Two independent clones (three and seven) were generated using the CRISPR/Cas9 system^74^. In brief, double-stranded DNA oligonucleotides that encode guide RNAs (gRNAs) against *ATG7* were cloned into the BbsI restriction sites of the pX459 vector. The gRNA oligonucleotides sequences used for the generation of the ATG7 KO lines were:

hATG7-gRNA-ex3-s 5’ caccgAACTCCAATGTTAAGCGAGC 3’

hATG7-gRNA-ex3-as 5’ aaacGCTCGCTTAACATTGGAGTTc 3’

Cells were transfected with the resulting vectors using XtremeGENE HP (Sigma Aldrich) according to the manufacturer’s protocol and selected with puromycin (2 µg/ml) for 5 days. Single cell clones were picked using cloning cylinders (Sigma-Aldrich), clones deficient for the targeted protein were identified by immunoblot and knock-out clones were validated by genomic DNA sequencing using the following primers: hATG7-in2-s: 5’ CCTGGCTGAGTCCCAGCTGTG 3’ hATG7-in3-as: 5’ GAAGACACTGCAGAGACTAC 3’. The respective control cell line was generated by using the empty vector and following the same procedure described above for the knockout cell lines.

### Mouse models of fibrosis

The functional role of the autophagy was investigated in the following mouse models of fibrosis: (i) In the model of bleomycin-induced dermal fibrosis, fibrosis was induced by local injections of bleomycin in C57BL/6NRj mice (Janvier Labs, Le Genest Saint Isle, France) for four weeks^75,76^. Subcutaneous injections of 0.9% NaCl, the solvent of bleomycin, served as control. (ii) In the TβRIact model, injections of Ad5 encoding TβRIact-induced localized skin fibrosis. Mice (C57BL/6NRj, Janvier Labs) injected with Ad5 encoding *LacZ* served as controls. 6.67 × 10^7^ ifu of Ad5 were injected intracutaneously every 2 weeks and mice were sacrificed after 8 weeks^77,78^. (iii) In the model of bleomycin-induced pulmonary fibrosis, bleomycin was applied intratracheally in C57BL/6NRj mice (Janvier Labs) using a high pressure syringe (Penn-Century, Wyndmoor, PA, USA)^73,79^. Instillation of equal volumes of 0.9% NaCl served as controls. The outcome was evaluated after four weeks. (iv) In the model of a TβRIact-induced pulmonary fibrosis, viral vectors (TβRIact or LacZ) were instilled intratracheally every 2 weeks and mice (C57BL/6NRj, Janvier Labs) were sacrificed after eight weeks. To follow autophagic flux in vivo, 6.67 × 10^7^ ifu of Ad5 encoding the autophagy reporter EGFP/RFP-LC3 was injected intracutaneously (C57BL/6NRj, Janvier Labs) once per week for 4 weeks.

To inhibit TGFβ signaling in vivo, the ATP-competitive transforming growth factor β receptor 1 (TGFβRI) inhibitor 2-(5-Chloro-2-fluorophenyl)-4-((4-pyridyl)amino)pteridine SD208 (Tocris, Bristol, UK) was administered orally at a doses of 20 mg/kg twice a day for four weeks. Mice used in this experiment had a C57BL/6NRj background (Janvier Labs). All animals used in this study were maintained in laboratory animal housing facilities under follow conditions: Temperature: 20–24 °C; Humidity: 45–65%, 12/12 h light/dark cycle. Animals were 6–8 weeks old at the beginning of the experiments.

### Plasmid and reporter constructs

The following expression and reporter plasmids were used: adenoviral expression constructs encodings murine *Beclin1* (Vector Biolabs, Malvern, PA, USA), murine *Myst1* (Applied Biological Materials, Richmond, Canada), In addition, we cloned the *MYST1* promoter (−684 bp to +7 bp) into pGL3-basic luciferase reporter backbone (Catalog #11979; Addgene, Watertown, MA, USA) (pGL3-*MYST1*-promoter) and mutated three predicted SBEs in the promoter sequence at residues -279 to -282 (SBE1), at residues -384 to -387 (SBE2) and at residues -574 to -577 (SBE3) either individually or in combination by exchanging GTCT to ATGT (pGL3-*MYST1*-ATGT) using the QuickChange Multi site-directed mutagenesis kit (Catalog# 200514; Agilent Technologies, Santa Clara, CA, USA). Primers used for mutagenesis are listed in Supplementary Table 1. Overexpression of *Myst1* in murine skin and lungs was achieved by intracutaneous injection or intratracheal instillation of Ad5 encoding for *Myst1* (Applied Biological Materials). Ad5 encoding *LacZ* served as controls. Autophagy was monitored as described using a reporter construct that encoded the rat LC3 cDNA fused to EGFP at the N-terminus and mRFP1 at the C-terminus of the insert (EGFP-LC3-RFP)^80^. The insert sequence was amplified from pMRX-IP-GFP-LC3-RFP^80^ (plasmid #84573; Addgene) and cloned in Ad5 using Adeno-X^TM^ Adenoviral System 3 kit (Clontech, California, CA, USA). The FLAG-(5 A) construct was generated by exchanging the non-tagged sequence of the pcDNA4TO-hproTGFβ1 construct^26^ with a synthethic DNA (GeneART Strings, Thermo Fisher Scientific, Waltham, MA) sequence containing the FLAG-tagged growth factor sequence flanked by the sequence encoding the first 5 amino acids of the growth factor (aa 279-283)^81^ using the internal KpnI and the XhoI cloning site of the pcDNA4TO-hproTGFβ1 construct.

### Modulation of autophagy in experimental fibrosis

To selectively disable autophagy in fibroblasts, we crossbred mice carrying two conditional alleles of *Atg7* (*Atg7*^fl/fl^, provided by Dr. Masaaki Komatsu^54^) with *Col1a2*;*Cre*ER mice^8,82^ to generate *Atg7*^fl/fl^x *Col1a2*;*Cre*ER mice. *Cre*-mediated recombination was induced by repeated intraperitoneal injections of 2 mg/mL tamoxifen over 5 days (*Atg7*^Fib^). *Atg7*^fl/fl^x *Col1a2*;*Cre*ER mice injected with corn oil served as controls (*Atg7*^control^). All mice were on a C57BL/6 background.

To stimulate autophagy in vivo, 6.0 × 10^7^ ifu of Ad5 encoding *Beclin1* (Vector Biolabs) were injected intracutaneously or intratracheally, respectively. To knockdown *Atg7* in vivo, we injected *Atg7* siRNA or non-targeting siRNA (pre-designed, Dharmacon, CA, USA) using the AteloGene in vivo siRNA/miRNA Transfection Kit (Reprocell, Glasgow, UK). One nmol of siRNA was injected subcutaneous once per week for three weeks. For pharmacological inhibition of autophagy in vivo, 3-methyladenine (3MA) (Sigma-Aldrich) was administered i.p. in a concentration of 15 mg/kg daily. Mice used in this experiment were on a C57BL/6NRj background (Janvier Labs).

### Quantitative real-time-PCR

Gene expression was quantified by SYBR Green real-time PCR using the ABI Prism 7300 Sequence Detection System (Life Technologies). Samples without enzyme in the reverse transcription reaction (Non-RT controls) were used as negative controls. Unspecific signals caused by primer dimers were excluded by non-template controls and by dissociation curve analysis. Beta-actin was used to normalize the amounts of cDNA within each sample^83^. Primer sequences are listed in the Supplementary Table 2. qRT-PCRs were performed using StepOnePlus™ Real-Time PCR System and analyzed with StepOne software version 2.3 (Thermo Fisher Scientific).

### Western blot analysis

Proteins were separated by sodium dodecyl sulfate polyacrylamide gel electrophoresis and transferred to a polyvinylidene difluoride membrane (PVDF). Membranes were incubated with primary antibodies listed in the Supplementary Table 3 and appropriate HRP-conjugated secondary antibodies (Dako, Glostrup, Denmark). Blots were visualized by ECL. β-actin was used as loading control (see Supplementary Table 3 for more information about antibodies). Western blot images were detected using ChemiDoc MP Imaging System (BioRad, Hercules, CA, USA) or X-ray film (GE Healthycare, Chicago, IL, USA). Blots were quantified using the ImageJ Software version 1.41 (National Institutes of Health, Bethesda, MD, USA) or Image Lab version 6.0.0 (BioRad).

### SILAC-based quantitative proteomics

For the quantitative analysis of the ATG7-dependent secretome, wild-type and ATG7 knock-out cells were pulse-labeled at 70% confluency. In brief, cells were pre-incubated with Met/Arg/Lys-free DMEM/F12 (Athena Enzyme Systems, Baltimore, MD, USA), supplemented with 1 mM pyruvate, 200 mg/l l-proline and dialyzed FCS (SILAC medium) for 30 min, to deplete intracellular Arg, Lys, and Met. Cells were then incubated for 24 h with 100 µM of the azide group-containing methionine analog azidohomoalanine (AnaSpec, Seraing, Belgium) in SILAC medium either supplemented with medium (146 µg/ml Lys-4 (^2^H), 84 µg/ml Arg-6 (^13^C_6_)) or heavy isotopes (146 µg/ml Lys-8 (^13^C_6_, ^15^N_2_)/84 µg/ml Arg-10 (^13^C_6_, ^15^N_4_)). All isotope-labeled amino acids were purchased from Silantes (Munich, Germany). Supernatants of wildtype heavy isotope-labeled and knock-out medium isotope-labeled cultures were combined, dead cells were removed by centrifugation (5 min, 1000 x *g*, 4 °C), and proteins were concentrated to approximately 250 µl, using Amicon Ultra-15 Centrifugal Filter Unit 3 kDa cutoff concentrator tubes (Sigma Aldrich). Samples from the inverse combination (wild-type medium isotope-labeled and knock-out heavy isotope-labeled cultures) were also prepared to control for labeling bias. Cells were lysed in 850 µl of urea lysis buffer provided with the Click Chemistry Capture kit (Jena Bioscience, Jena, Germany), according to the manufacturer’s protocol. Supernatants and cell lysates were individually covalently coupled to 200 µl pre-washed alkyne agarose (Jena Bioscience) and rotated overnight at room temperature. Alkyne agarose-bound samples were spun down (5 min, 2000 x *g*, 4 °C), washed once with 1 ml HPLC water, reduced with 1 mM DTT, alkylated with 40 mM chloroacetamide, transferred to columns (supplied with the Click Chemistry Capture kit) and extensively washed sequentially with SDS wash buffer (supplied with Click Chemistry Capture kit), 100 mM Tris-HCl, pH 8.0/8 M urea buffer, 20% isopropanol, and 20% acetonitrile (20 ml each). Proteins were then digested overnight with 1 µg trypsin (Serva, Heidelberg, Germany) and 0.5 µg LysC (FUJIFILM Wako Chemicals, Neuss, Germany) in a heated shaker (37 °C, 800 rpm). Digestion was stopped by adding 0.5% formic acid to the reaction, and the peptides subjected to StageTip purification using styrene-divinylbenzene reverse-phase sulfonate (SDB-RPS) StageTips with two layers of SDB-RPS discs before injection into the mass spectrometer.

### Mass spectrometry analysis

Proteomic analysis was performed using an Easy nLC 1000 UHPLC coupled to a QExactive Plus mass spectrometer (Thermo Fisher Scientific). Peptides were resuspended in Solvent A, picked up with an autosampler and loaded onto in-house made 50 cm fused silica columns (internal diameter 75 µm, packed with C18 Poroshell beads, 2.7 µm, Agilent) at a flow rate of 0.75 µl/min. Eluted peptides were sprayed into the heated transfer capillary of the mass spectrometer using a nano-electrospray ion source (Thermo Fisher Scientific). The mass spectrometer was operated in a data-dependent mode, where the Orbitrap acquired full MS scans (300–1750 *m*/*z*) at a resolution (R) of 70,000 with an automated gain control (AGC) target of 3 × 10^6^ ions collected within 20 ms. The dynamic exclusion time was set to 20 s. From the full MS scan, the 10 most intense peaks (*z* ≥ 2) were fragmented in the high-energy collision-induced dissociation (HCD) cell. Collisional energy, ion target and maximum injection time were adapted for the different input samples. The raw files were processed using MaxQuant software v1.5.3.8 (Max-Planck-Institute of Biochemistry, Martinsried, Germany) and its implemented Andromeda search engine^84^. Parameters were set to default values and SILAC labels were included as described in the proteomics section. Settings and parameters used for the mass spectrometry analysis are described in the Supplementary Table 4.

### Quantification of active TGFβ by mink lung cell (MLC) assay

The level of active TGFβ was evaluated by MLC transfected with a fused sequence of firefly luciferase and PAI-1 promoter (kindly provided by Daniel Rifkin^85^). In all, 4 × 10^4^ MLCs were plated into a 96-well plate and incubated overnight at 37 °C. On the next day, 100 µl of fibroblast-conditioned medium or 20 µl of diluted murine skin lysates (100–300 μg protein diluted in 500 µl DMEM medium with 0.1% bovine serum albumin (BSA)) were added to the medium of pre-plated MLCs. After 20–24 h of stimulation, luciferase reporter activity was determined by using a microplate luminometer Centro LB 960 (Berthold Technologies, Bad Herrenalb, Germany). The value of luminescence was further converted into concentration by using a standard curve with a series dilution of recombinant TGFβ^86^.

### Reporter assays

NIH3T3 mouse embryonic fibroblasts were transfected with different luciferase reporter constructs or pSv-β-galactosidase (β-gal) using FuGENE HD Transfection Reagent (Promega) or infected with Ad5 luciferase reporter constructs. Luciferase activity was normalized for transfection efficiency to internal pSv-β-galactosidase. Luciferase activities were determined using a microplate luminometer Centro LB 960 (Berthold Technologies) and data was collected using Step 7 MicroWin v4.0 Sp9 software (Labsis Laborsysteme, Neunkirchen-Seelscheid, Germany). To monitor the effects of TGFβ on autophagy in vitro, cells were infected with Ad5 encoding *EGFP-LC3-RFP* (described in the section plasmid and reporter constructs), an autophagy activity reporter. After infection, cells were incubated with different inhibitors: Vorinostat (1 µM), Trichostatin A (0.4 µM) (both Selleckchem, Houston, TX, USA), 5-Aza-2’-deoxycytidine (10 µM, Sigma-Aldrich) and 3-deazaneplanocin A (10 µM, Tocris). In addition, *Myst1* was knockdown using siRNA or overexpressed using adenoviral vectors. Fluorescence was detected by high-throughput real-time imaging technology using IncuCyte Live-Cell Analysis System (Essen Bioscience, Royston Hertfordshire, UK). Data was collected by Incucyte® S3 Software V2018A (Essen Bioscience).

### Immunohistochemistry and immunofluorescence staining

Formalin-fixed, paraffin-embedded skin sections or cultured cells fixed with 4 % PFA and permeabilized with 0.25 % Triton X100 were stained with appropriate primary antibodies (see Supplementary Table 3). HRP-conjugated or Alexa Fluor antibodies (Life Technologies) were used as secondary antibodies. Isotype control antibodies were used as controls. Stress fibers were visualized with rhodamine-conjugated phalloidin (Sigma-Aldrich) (see Supplementary Table 3 for more information). In addition, cell nuclei were stained using DAPI (Santa Cruz Biotechnology, Heidelberg, Germany). Images were captured using a Nikon Eclipse 80i fluorescence microscope and data was collected using NIS-Elements BR version 5.20.01 software (Nikon, Badhoevedorp, Netherlands). Samples of mice tissue injected with Ad5 encoding EGFP-LC3-RFP (autophagy reporter) were immunostained for EGFP using polyclonal goat-anti-GFP antibody (Abcam, Cambridge, UK) and RFP using monoclonal rabbit anti-RFP (Abcam) and analyzed by fluorescence microscopy using the same microscope described above (see Supplementary Table 3 for more information about antibodies used). Fluorescence intensity was quantified using ImageJ/Fiji software (National Institutes of Health).

Costainings of LC3B and P62 with LAMP2 (see Supplementary Table 3) in skin tissue were analyzed by confocal microscopy using the Leica SP5 II microscope (Leica Microsystems, Wetzlar, Germany) and data was collected using Leica Application Suite X version 4.12.0.86 (Leica Mycrosystems). Confocal images were deconvoluted by using DeconvolutionLab2 (EPFL, Lausanne, Switzerland) based on corresponding optical parameters^87^.

Quantification was performed with ImageJ software (version 1.41; National Institutes of Health) as follows: a density threshold was set to quantify the positive staining by using the respective negative controls. The threshold was selected to exclude unspecific background staining. The same thresholds and system settings were used for all slides. The number of pixels falling within the threshold, indicating the quantity of positive staining, was recorded for each area.

### Apoptosis detection

To monitor apoptosis in vitro, we used Caspase- 3/7 Green Apoptosis Reagent (Essen Bioscience) according to the recommendations of the manufacturer. Fluorescence was detected by high-throughput real-time imaging technology using IncuCyte Live-Cell Analysis System (Essen Bioscience). Data was collected by Incucyte® S3 Software V2018A (Essen Bioscience). As positive control for apoptosis, cells were treated with 0.1 µM staurosporine (Tocris). In vivo, apoptosis was detected using TACS in situ apoptosis detection kit (Trevigen, Gaithersburg, MD, USA). Images were captured using the fluorescence microscopy Nikon Eclipse 80i microscope and the software NIS-Elements BR version 5.20.01 (Nikon).

### Myofibroblast re-differentiation assays

Human fibroblasts were cultured in 96-well plates. After 24 h, we differentiated fibroblasts into myofibroblasts by incubation with TGFβ for three days, with re-stimulation with TGFβ every twenty-four hours. We then treated the cells with CQ (10 µM, Biotrend) or 3-MA (5 mM, Sigma-Aldrich). Alternatively, we knocked down *ATG7* and *BECLIN1* (50 nM) (pre-designed, Invitrogen, CA, USA), via siRNA transfection using Viromer Blue transfection reagent (OriGene Technologies, Rockville, MD, USA), and continued the stimulation with recombinant TGFβ for two additional days. The control group (Fib) was pre-treated with vehicle for three days, then transfected with non-targeting siRNA and treated with vehicle for additional two days. The deactivated myofibroblast group (deact MyoFib) was pre-treated with TGFβ for three days, then transfected with non-targeting siRNA and treated with vehicle for two days. Afterwards, cells were fixed with 4% PFA and immunostained for stress fibers and αSMA as described above (immunohistochemistry and immunofluorescence staining section). Images were captured using CellInsight CX5 High-Content Screening (HCS) Platform (Thermo Fisher Scientific; software HCS Navigator Version 6.6.1). Data were analyzed using HCS Studio Cell Analysis Software 6.6.1 (Thermo Fisher Scientific).

### Extracellular matrix Immunofluorescence staining

Fibroblasts were platted in a 96-well, black-walled imaging plates (BD Biosciences, NJ, USA) at a density of 4 × 10^3^ cells/well. Fibroblasts were incubated for 5 days with or without treatment. Afterwards, plates were washed with PBS and cells were removed with 0.25 M ammonium hydroxide in 25 mM Tris for 15 min at 37 °C. The plate with the remaining extracellular matrix (ECM) was washed three times with PBS and fixed with 100% methanol for 30 min at −20 °C and finally washed three more times with PBS. ECM was immunostained with monoclonal mouse anti-Fibronectin antibodies conjugated with Alexa Fluor 488 (eBiosciences, Frankfurt am Main, Germany), polyclonal rabbit anti-Collagen Type I antibodies (Merck Millipore, Darmstadt, Germany) and polyclonal rabbit anti-Collagen Type III antibodies (Merck Millipore). See Supplementary Table 3 for more information about antibodies used in this experiment. Samples were analyzed using CX5-based automated quantification of extracellular matrix (Thermo Fisher Scientific; software HCS Navigator Version 6.6.1). Data were analyzed using HCS Studio Cell Analysis Software 6.6.1 (Thermo Fisher Scientific).

### Transmission electron microscopy

For transmission electron microscopy, cells were harvested and washed with 0.1 M phosphate buffer (PB), followed by fixing with 2% glutaraldehyde and 2% paraformaldehyde in 0.1 M PB for 3 min, and post-fixation with 1% osmic acid for 1 h. The samples were then dehydrated in graded ethanol, washed with propylene oxide, and embedded in epoxy resin. Samithin and Ultrathin sections cut in a Reichert ultramicrotome were stained with lead citrate and uranyl acetate and were examined in a HITACHI H-7500 TEM at 100 kV. Images were captured with AMT XR-16 16 mp high-resolution CCD camera system in combination with the AMT Capture Engine, v602.600.51 (Woburn, MA, USA).

### Chromatin immunoprecipitation (ChIP) assays

ChIP assays were performed using the ChIP-IT Express Kit (Active Motif, Carlsbad, CA, USA). Sonicated chromatin extracts were incubated with antibodies against SMAD3 (Cell signaling) or normal rabbit IgG antibodies (Santa Cruz Biotechnology). See Supplementary Table 3 for more information about the antibodies used. Bound DNA sequences were determined by quantitative real-time PCR. Primer sequences are listed in the Supplementary Table 5.

### Evaluation of experimental fibrosis

The extent of fibrosis was analyzed using histological, biochemical, immunohistochemical and mRNA readouts. Histologic readouts included quantification of the dermal thickness as analyzed by measuring the distance between the epidermal-dermal junction and the dermal-subcutaneous fat junction on HE stained sections at eight sites at 100-fold magnification, evaluation of the fibrotic area as percent of total lung area in Sirius Red stained sections; quantification of pulmonary changes using the Ashcroft score and direct visualization of collagen by trichrome staining^83,88,89^. The total collagen content was analyzed biochemically using hydroxyproline assays and by evaluation of *Col1a1* mRNA. In addition, myofibroblasts were identified immunohistochemically as α-smooth muscle actin positive fibroblasts at four different sites in a tissue^90,91^. To quantify the pulmonary fibrosis in animal models, we analyzed histological sections and classified the changes with scores from 0 to 8 according to the Ashcroft scale^92,93^. Images were captured using Nikon Eclipse 80i microscope in combination with NIS-Elements BR version 5.20.01 software (Nikon) or Nano Zoomer S60 slide scanner using NDP.scan 3.3 software for data collection and NDP.view 2 software for analysis (Hamamatsu Photonics, Hamamatsu City, Japan).

### Statistics and reproducibility

The sample size was estimated based on previous experiments. No statistical method was used to predetermine sample size. Experiments and quantifications were not done in a blinded fashion. There were no exclusion criteria for the human and animal experiments. Mice were stratified according to sex and then randomized into the different treatment groups. Cells from human donors were also randomized. Unless otherwise noted, each experiment was repeated at least three times independently. Data shown in column graphs are presented as median ± interquartile range (IQR) as indicated in the figure legends, and individual data points are plotted. Differences between the groups were tested for their statistical significance by Mann–Whitney *U* non-parametric test for non-related samples and by ANOVA one-way with Sidak’s multiple comparisons test or Tukey’s multiple comparison post hoc test for comparisons involving more than two groups. Information about *p*-values and statistical tests used in this article can be found in the figure legends and in the source data file. Statistic was analyzed using GraphPad prism 8 version 8.3.0 (GraphPad Software, San Diego, CA, USA). For SILAC experiments a one-sample *t-*test was performed (Value = 0, S0 = 0.1, side = both) and −log [*p*-value] was calculated using Perseus version 1.6.5^94^.

### Study approval

The human studies were approved by the Ethical committee of the Medical Faculty of the University of Erlangen-Nuremberg. All patients and controls signed a consent form approved by the review board of the institution mentioned above. All human studies were performed in compliance with the relevant ethical regulations of the Medical Faculty of the University of Erlangen-Nuremberg. All animal experiments were approved by the governments of Mittelfranken or Unterfranken, Germany. All animal experiments were performed in compliance with the relevant ethical regulations of the governments of Mittelfranken or Unterfranken, Germany.

### Reporting summary

Further information on research design is available in the Nature Research Reporting Summary linked to this article.

## Supplementary information

Supplemantary information

Reporting Summary

## Data Availability

All data generated or analyzed during this study are included in this article and its supplementary information files. Source data are provided with this article. Uncropped western blot images are provided in the source data file. Additional detailed information is available from the corresponding author on reasonable request. The proteomics data are available via the PRIDE database with the dataset identifier PXD025001. As long as the full PRIDE dataset has not been publicly released, login credentials to the PRIDE reviewer account will be provided upon reasonable request (JN). Source data are provided with this paper.

## Source data

Source data
