# Supplementary material for: TGFβ promotes fibrosis by MYST1-dependent epigenetic regulation of autophagy
Source: Nat Commun. 2021 Jul 20;12:4404. doi: 10.1038/s41467-021-24601-y (PMC8292318; doi:10.1038/s41467-021-24601-y)
Supplement: Supplementary file 1 — Supplemantary information [file 41467_2021_24601_MOESM1_ESM.pdf]

# Supplementary Information:

# TGFβ promotes fibrosis by MYST1-dependent epigenetic regulation of autophagy

**Zehender *et al*.**

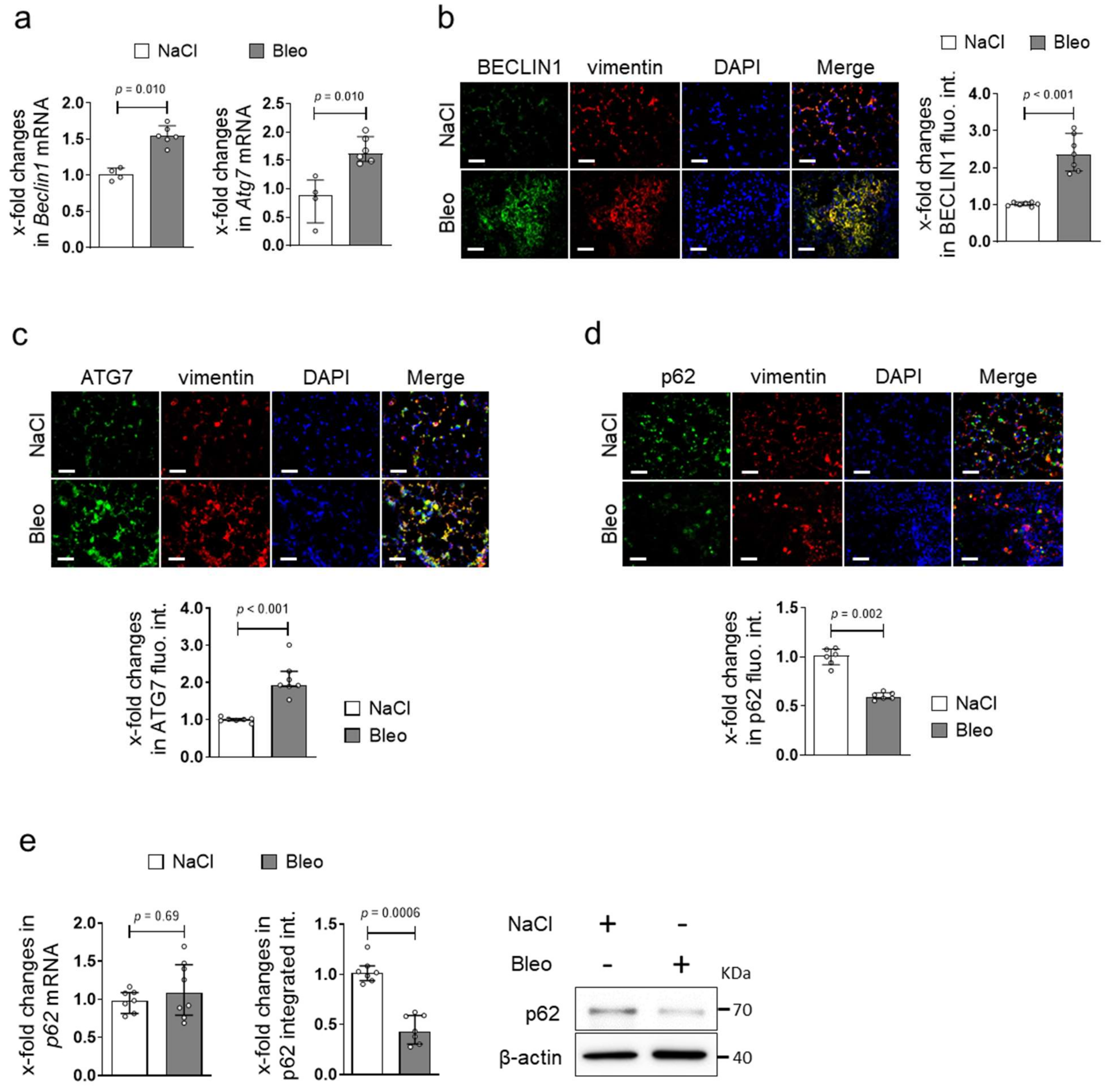


**Supplementary Figure 1: Induction of autophagy related proteins in bleomycin-induced lung and skin fibrosis. A:** mRNA levels of *Beclin1* and *Atg7* (n = 4 biological replicates for control and n = 6 for bleomycin group). **B-D:** Representative immunofluorescence staining of BECLIN1 (**B**), ATG7 (**C**) or p62 (**D**) (all green) in combination with DAPI (blue) and vimentin (red) and respective quantifications of BECLIN1, ATG7 (n = 7 biological replicates per group) and p62 (n= 6 biological replicates per group) expression. Horizontal scale bars, 50 µm. **E:** *p62* mRNA (n = 7 biological replicates for control and n = 8 for bleomycin group) and protein levels (n = 7 biological replicates per bleomycin group) in murine skin. Representative western blot and quantification. All data are presented as median ± IQR. *p*-values were determined by two-sided Mann-Whitney test and are indicated in the figure. See source data for more detailed information. int.: intensity, fluo.: fluorescence., Bleo: bleomycin.

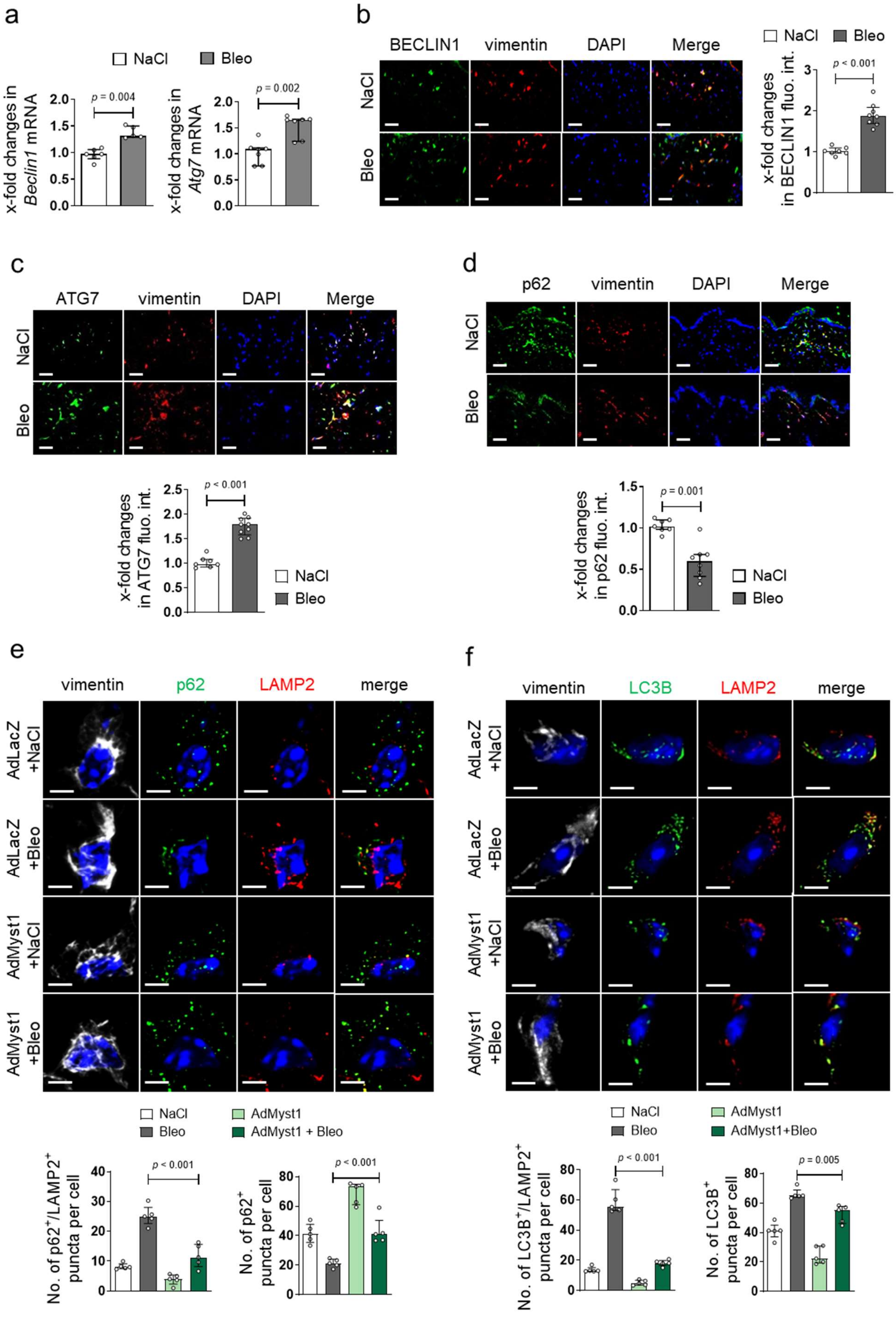
a
NaCl
Bleo
x-fold changes in Beclin1 mRNA
p = 0.004
x-fold changes in Atg7 mRNA
p = 0.002
b
BECLIN1
vimentin
DAPI
Merge
NaCl
Bleo
x-fold changes in BECLIN1 fluo. int.
p < 0.001
c
ATG7
vimentin
DAPI
Merge
x-fold changes in ATG7 fluo. int.
p < 0.001
d
p62
vimentin
DAPI
Merge
x-fold changes in p62 fluo. int.
p = 0.001
e
vimentin
p62
LAMP2
merge
AdLacZ +NaCl
AdLacZ +Bleo
AdMyst1 +NaCl
AdMyst1 +Bleo
NaCl
Bleo
AdMyst1
AdMyst1 + Bleo
No. of p62+/LAMP2+ puncta per cell
p < 0.001
No. of p62+ puncta per cell
p < 0.001
f
vimentin
LC3B
LAMP2
merge
AdMyst1+Bleo
No. of LC3B+/LAMP2+ puncta per cell
p < 0.001
No. of LC3B+ puncta per cell
p = 0.005

**Supplementary Figure 2: Autophagy is activated in bleomycin-induced skin fibrosis. A:** mRNA levels of *Beclin1* (n = 6 biological replicates for control and n = 5 for bleomycin group) and *Atg7* (n = 7 biological replicates per group) **B-D:** Representative immunofluorescence staining of BECLIN1 (**A**; n = 7 biological replicates for control and n = 8 for bleomycin group), ATG7 (**C;** n = 7 samples for control and n = 9 for bleomycin group) or p62 (**D;** n = 7 biological replicates for control and n = 8 for bleomycin group) (all green) in combination with DAPI (blue) and vimentin (red). Horizontal scale bars, 50 µm. **E-F: Autophagic flux in bleomycin-induced skin fibrosis. E:** Co-staining of p62 (green) and LAMP2 (red) in combination with DAPI (blue) and vimentin (gray) (n = 5 biological replicates per group). Representative confocal images and quantifications. **F:** Co-staining of LC3B (green) and LAMP2 (red) in Combination with DAPI (blue) and vimentin (gray) (n = n = 5 biological replicates per group): Representative confocal images and quantifications. Horizontal scale bars, 5 µm. All data are presented as median ± IQR. *p*-values were determined by two-sided Mann-Whitney test (**A-D**) or ANOVA one-way with Tukey's multiple comparison post hoc test (**E-F**) and are indicated in the figure. See source data for more detailed information. Fluo.: fluorescence, Int.: intensity, Bleo: bleomycin, Ad: adenovirus.

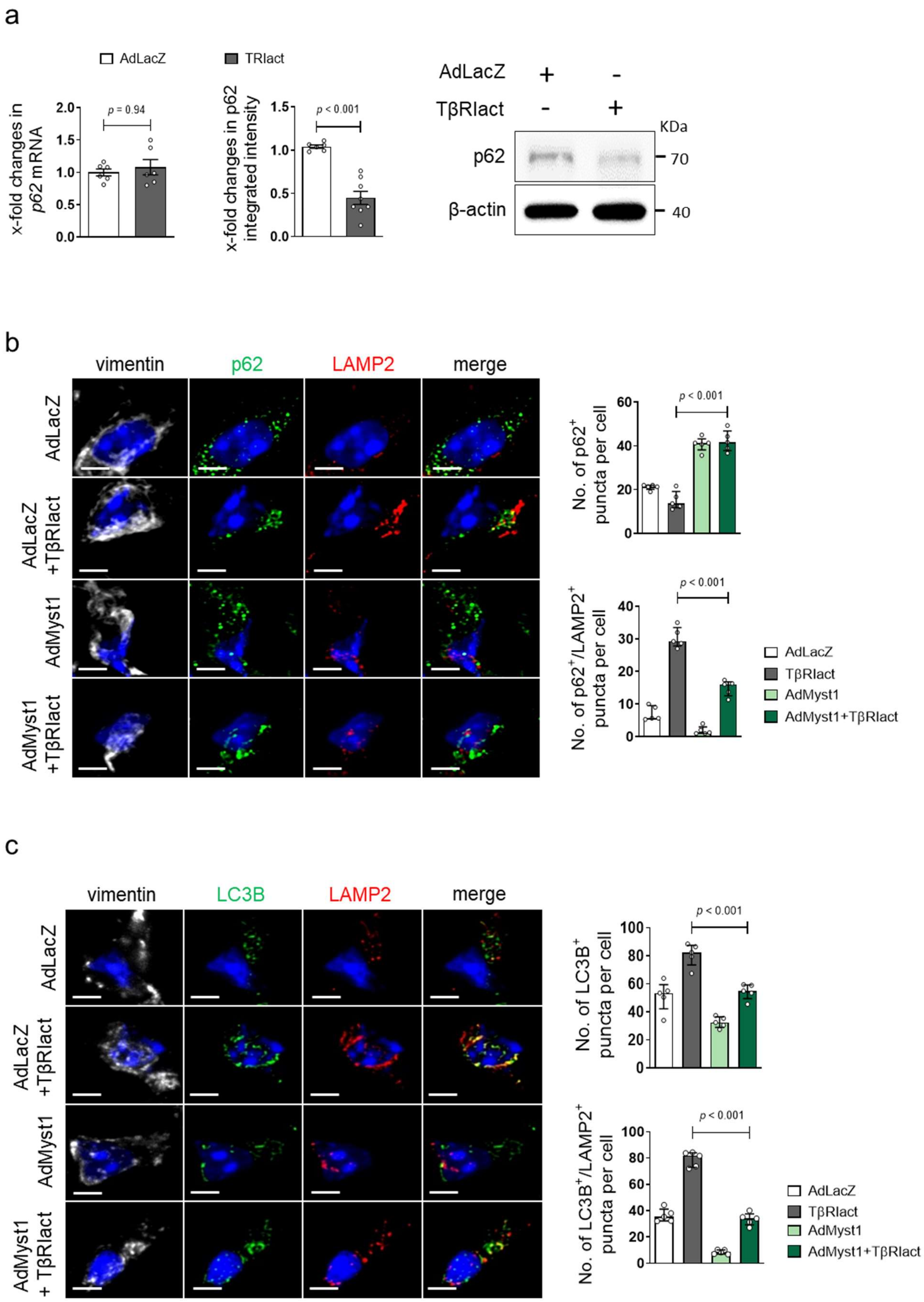
a
AdLacZ
TRIact
x-fold changes in p62 mRNA
p = 0.94
x-fold changes in p62 integrated intensity
p < 0.001
AdLacZ + -
TβRIact - +
KDa
p62
70
β-actin
40
b
vimentin
p62
LAMP2
merge
AdLacZ
AdLacZ +TβRIact
AdMyst1
AdMyst1 +TβRIact
No. of p62+ puncta per cell
p < 0.001
No. of p62+/LAMP2+ puncta per cell
p < 0.001
AdLacZ
TβRIact
AdMyst1
AdMyst1+TβRIact
c
vimentin
LC3B
LAMP2
merge
AdLacZ
AdLacZ +TβRIact
AdMyst1
AdMyst1 + TβRIact
No. of LC3B+ puncta per cell
p < 0.001
No. of LC3B+/LAMP2+ puncta per cell
p < 0.001
AdLacZ
TβRIact
AdMyst1
AdMyst1+TβRIact

**Supplementary Figure 3: Autophagy is activated in TβRIact-induced skin fibrosis. A:** *p62* mRNA (n = 6 biological replicates per group) and protein levels (n = 6 biological replicates for control and n = 8 samples for TβRIact group). Representative western blot and quantification. **B:** Co-staining of p62 (green) and LAMP2 (red) in combination with DAPI (blue) and vimentin (gray). Representative confocal images and quantifications (n = 5 biological replicates per group). **C:** Co-staining of LC3B (green) and LAMP2 (red) in combination with DAPI (blue) and vimentin (gray): Representative confocal images and quantifications (n = 5 biological replicates per group). Horizontal scale bar, 5 µm. All data are presented as median ± IQR. *p*-values were determined by two-sided Mann-Whitney test (**A**) or ANOVA one-way with Tukey's multiple comparison post hoc test (**B-C**) and are indicated in the figures. See source data for more detailed information. Ad: Adenovirus, TβRIact: constitutively active TGFβ receptor type I.

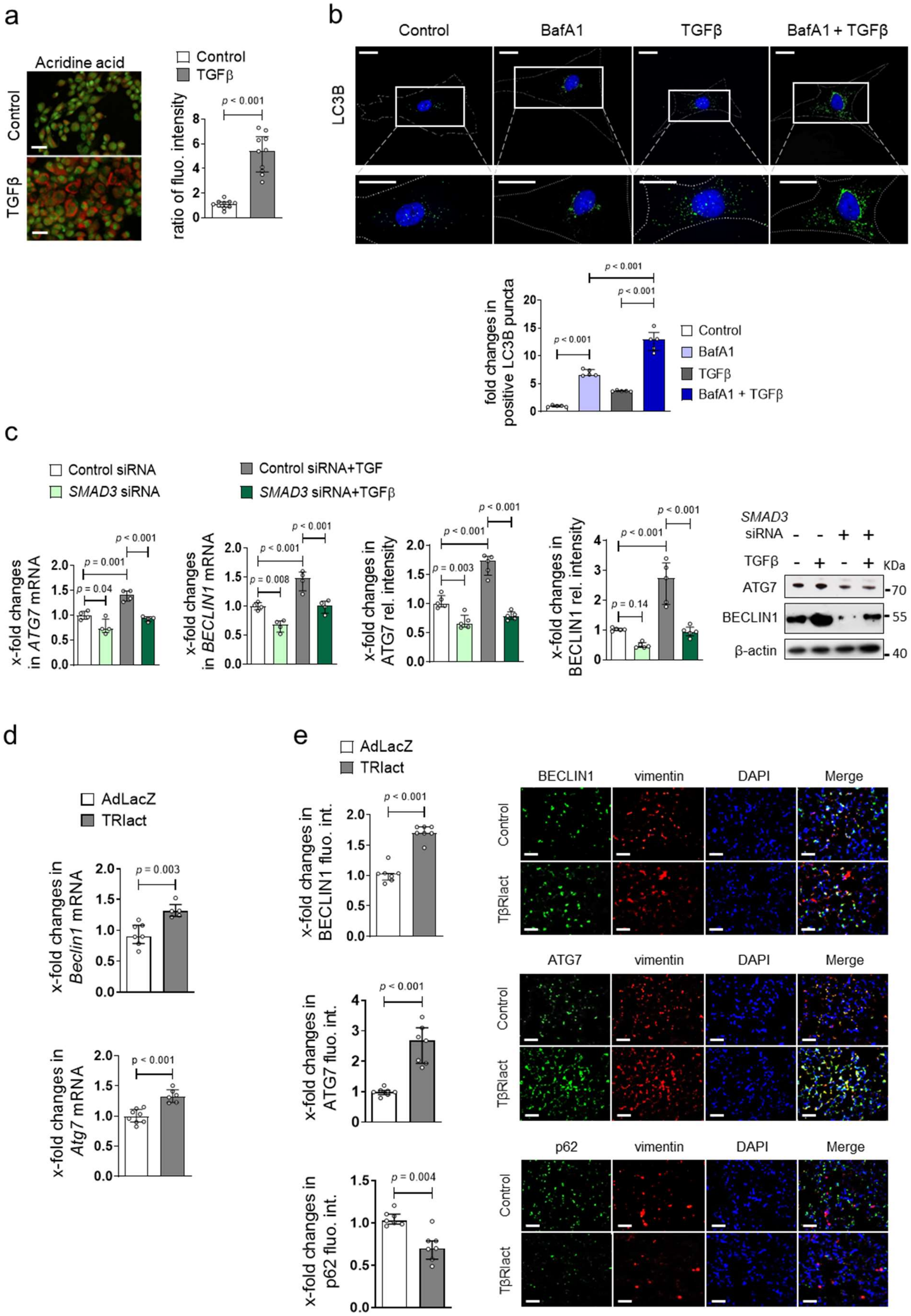

a
Acridine acid
Control
TGFβ
Control
TGFβ
ratio of fluo. intensity
p < 0.001
b
Control
BafA1
TGFβ
BafA1 + TGFβ
LC3B
fold changes in positive LC3B puncta
p < 0.001
Control
BafA1
TGFβ
BafA1 + TGFβ
c
Control siRNA
SMAD3 siRNA
Control siRNA+TGF
SMAD3 siRNA+TGFβ
x-fold changes in ATG7 mRNA
p = 0.04
p = 0.001
p < 0.001
x-fold changes in BECLIN1 mRNA
p = 0.008
x-fold changes in ATG7 rel. intensity
p = 0.003
x-fold changes in BECLIN1 rel. intensity
p = 0.14
SMAD3 siRNA
TGFβ
KDa
ATG7
BECLIN1
β-actin
70
55
40
d
AdLacZ
TRIact
x-fold changes in Beclin1 mRNA
p = 0.003
x-fold changes in Atg7 mRNA
p < 0.001
e
AdLacZ
TRIact
x-fold changes in BECLIN1 fluo. int.
x-fold changes in ATG7 fluo. int.
x-fold changes in p62 fluo. int.
p = 0.004
BECLIN1
vimentin
DAPI
Merge
Control
TβRIact
ATG7
p62

**Supplementary Figure 4: TGFβ / Smad3-dependent regulation of autophagy-regulators in fibroblasts. A:** Representative images and quantification of acridine staining in fibroblasts (n = 10 independent quantifications within 3 biological replicates per group). **B:** Representative images and quantification of LC3B fluorescence staining in fibroblasts treated with TGFβ in the presence or absence of BafA1 (n = 5 biological replicates per group). Horizontal scale bar, 5 µm. **C:** Effects of siRNA-mediated knockdown of SMAD3 on ATG7 and BECLIN1 mRNA (n = 4 biological replicates per group) and protein (n = 5 biological replicates per group) in TGFβ stimulated fibroblasts. **D-E:** Overexpression of TβRI in murine lungs: mRNA levels of *Beclin1* (**D**; n = 7 samples for AdLacZ and n = 5 for TβRIact) and *Atg7* (n = 8 biological replicates for AdLacZ and n = 6 for TβRIact) and representative stainings of BECLIN1, ATG7 and p62 (all green) in combination with DAPI (blue) and vimentin (red) and respective quantifications (**E**; n = 7 biological replicates per group). Horizontal scale bar, 50 µm. All data are presented as median ± IQR (**C-E**). *p*-values were determined by two-sided Mann-Whitney test and are indicated in the figure. See source data for more detailed information. rel.: relative, fluo.: fluorescence, int.: intensity, TβRIact: constitutively active TGFβ receptor type I, BafA1: Bafilomycin A1.

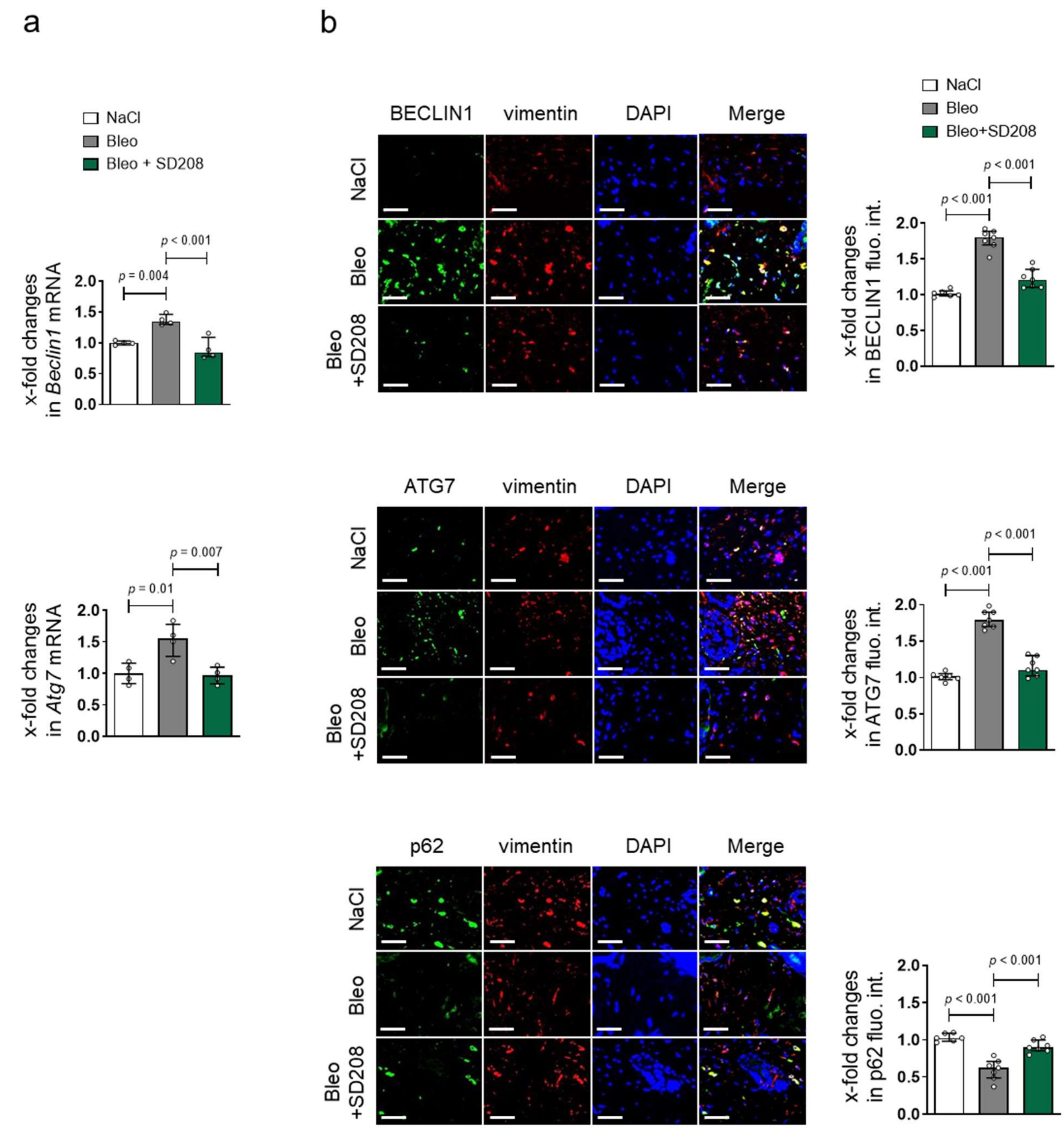


**Supplementary Figure 5: Selective inhibition of TGFβ signaling by SD-208 decreases the expression of autophagy markers in bleomycin-induced fibrosis. A:** mRNA levels of *Beclin1* and *Atg7* (n = 4 biological replicates per group). **B:** Representative immunofluorescence staining of markers of autophagy BECLIN1, ATG7 or p62 (all green; n = 6 biological replicates for control group and n = 7 for other groups) in combination with DAPI (blue) and vimentin (red). Horizontal scale bar, 50 μm. All data are presented as median ± IQR. *p*-values were determined by ANOVA one-way with Tukey's multiple comparison post hoc test and are indicated in the figure. See source data for more detailed information. int.: intensity, fluo.: fluorescence., Bleo: bleomycin.

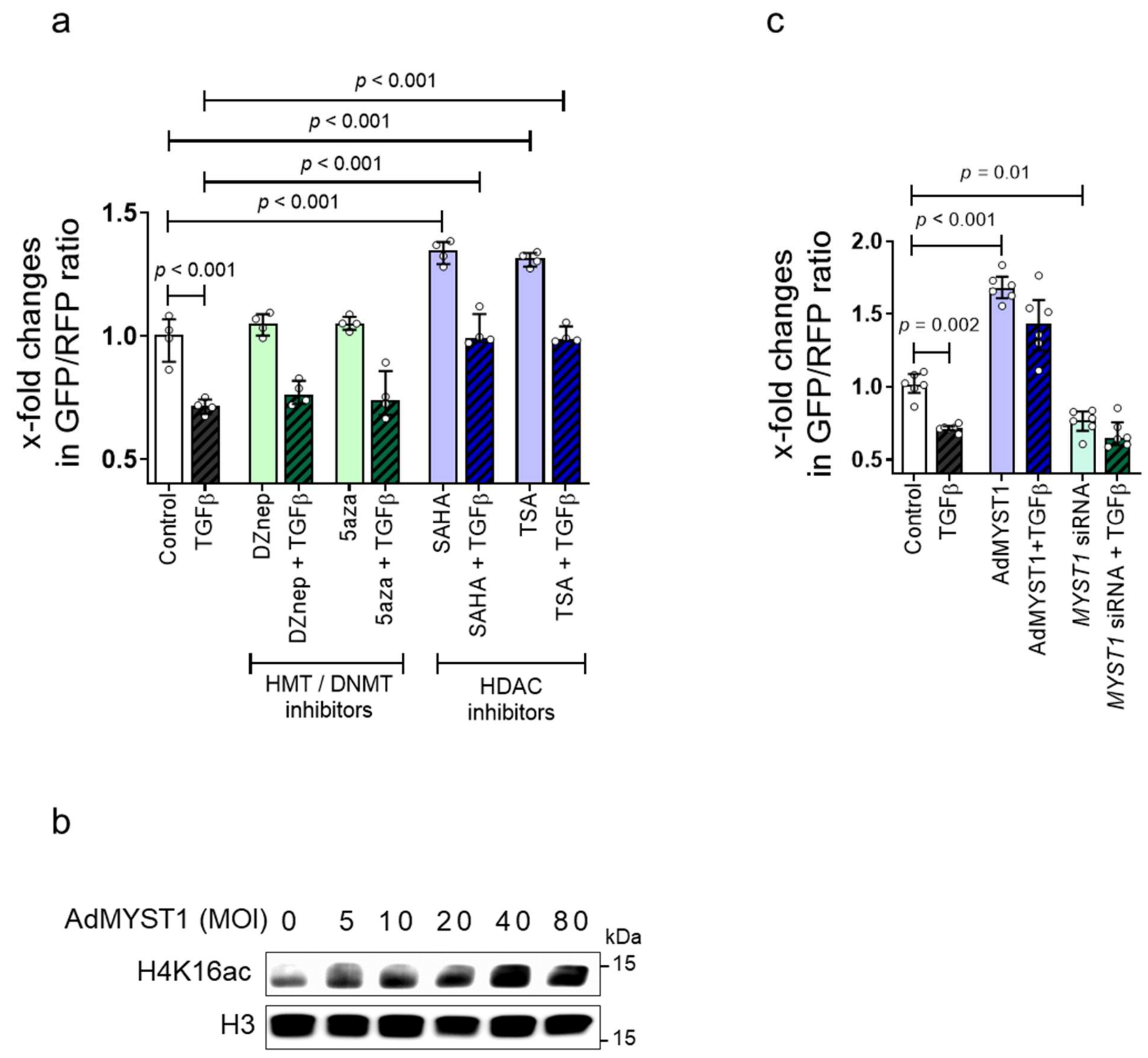


**Supplementary Figure 6: MYST1 acetyltransferase modulates TGFβ-induced autophagy.** EGFP/RFP-LC3 reporter activity, in which the GFP/RFP ratio correlates inversely with autophagy activity: **A:** Inhibition of different epigenetic mechanisms that have been linked to the pathogenesis of fibrosis including H3K27 histone methylation (DZnep), DNA methyltransferases (5aza) and HDACs (TSA and SAHA) in TGFβ-stimulated fibroblasts (n = 4 biological replicates per group). **B:** Histone acetylation levels in cultured fibroblasts upon adenoviral overexpression of MYST1. Representative Western blot images of fibroblasts infected with different concentrations of adenovirus encoding for *MYST1*. **C:** GFP/RFP ratio upon overexpression and knockdown of *MYST1* in fibroblasts (n = 6 biological replicates per group). All Data are presented as median ± IQR. *p*-values were determined by ANOVA one-way with Tukey's multiple comparison post hoc test and are indicated in the figure. See source data for more detailed information. Ad: Adenovirus. DZnep: 3-deazaneplanocin A, 5aza: 5-Aza-2'-deoxycytidine; TSA: Trichostatin A; SAHA: Vorinostat. The samples in panels A and C are part of a large set of experiments and share the same controls. Data are presented separately just for the purpose of this figure.

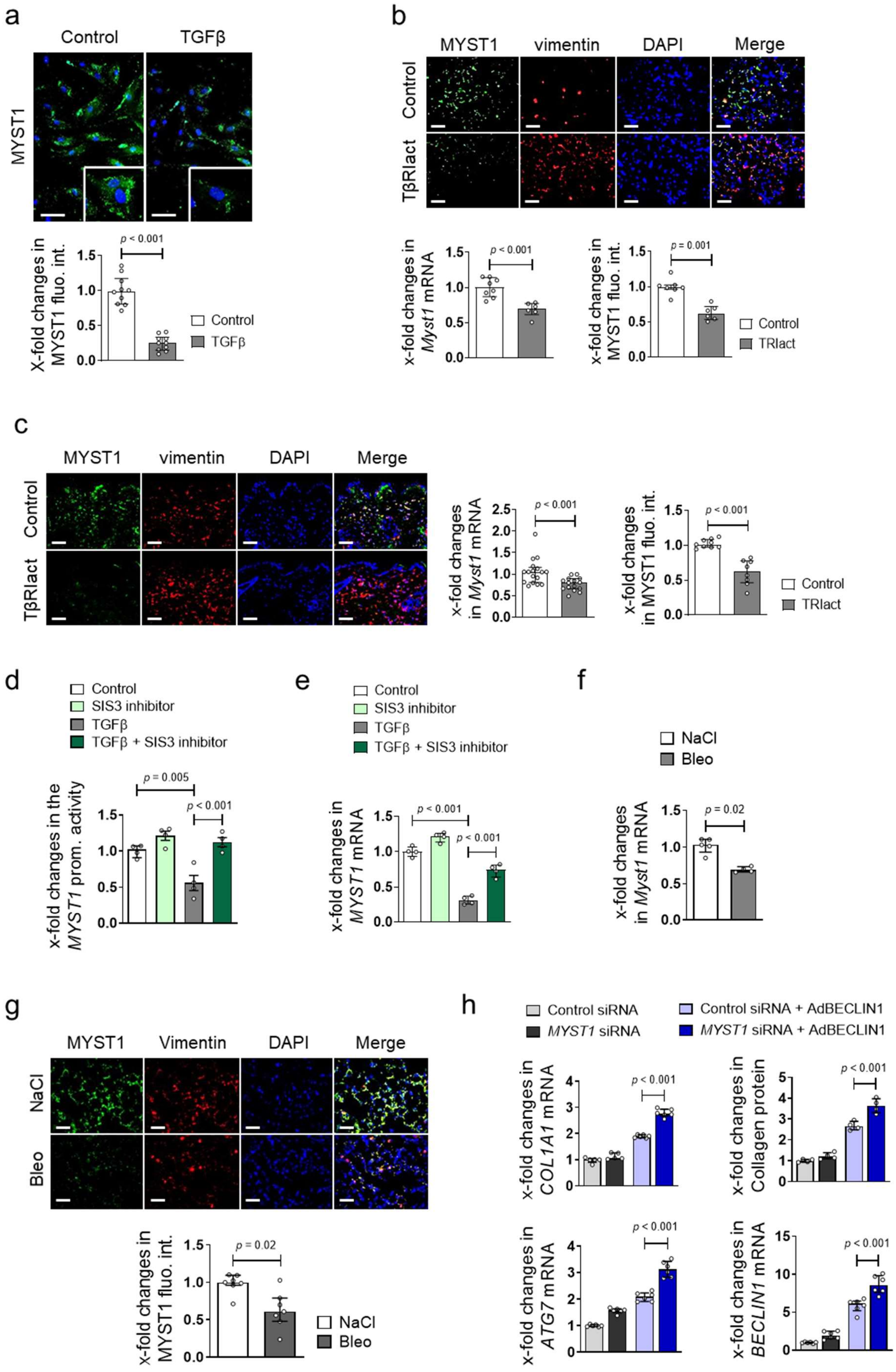
a
Control
TGFβ
MYST1
X-fold changes in MYST1 fluo. int.
p < 0.001
Control
TGFβ
b
MYST1
vimentin
DAPI
Merge
Control
TβRIact
x-fold changes in Myst1 mRNA
p < 0.001
x-fold changes in MYST1 fluo. int.
p = 0.001
Control
TRIact
c
MYST1
vimentin
DAPI
Merge
Control
TβRIact
x-fold changes in Myst1 mRNA
p < 0.001
x-fold changes in MYST1 fluo. int.
p < 0.001
Control
TRIact
d
Control
SIS3 inhibitor
TGFβ
TGFβ + SIS3 inhibitor
x-fold changes in the MYST1 prom. activity
p = 0.005
p < 0.001
e
Control
SIS3 inhibitor
TGFβ
TGFβ + SIS3 inhibitor
x-fold changes in MYST1 mRNA
p < 0.001
p < 0.001
f
NaCl
Bleo
x-fold changes in Myst1 mRNA
p = 0.02
g
MYST1
Vimentin
DAPI
Merge
NaCl
Bleo
x-fold changes in MYST1 fluo. int.
p = 0.02
NaCl
Bleo
h
Control siRNA
MYST1 siRNA
Control siRNA + AdBECLIN1
MYST1 siRNA + AdBECLIN1
x-fold changes in COL1A1 mRNA
p < 0.001
x-fold changes in Collagen protein
p < 0.001
x-fold changes in ATG7 mRNA
p < 0.001
x-fold changes in BECLIN1 mRNA
p < 0.001

**Supplementary Figure 7: TGFβ downregulates MYST1 in a SMAD3-dependent manner. A: Expression of MYST1 in human dermal fibroblasts stimulated with recombinant TGFβ.** Representative immunofluorescence stainings of MYST1 (green) in combination with DAPI (blue) (400-fold magnification) and quantification of fluorescence intensity (n = 10 independent quantifications within 3 biological replicates per group). **B:** Levels of MYST1 upon adenoviral overexpression of TβRIact in murine lungs as analyzed by qRT-PCR (n = 8 biological replicates for control and n = 6 for TβRIact group) and immunofluorescence staining for MYST1 (green), vimentin (red) and DAPI (blue) at 400-fold magnification (n = 7 biological replicates for control and n = 6 for TβRIact group). **C:** Effect of TβRIact overexpression on MYST1 levels: mRNA (n = 17 biological replicates for control and n = 15 for TβRIact group) and immunofluorescence staining (n = 9 biological replicates for control and n = 8 for TβRIact group) for MYST1 in murine skin. **D-E:** Activity of the *MYST1* promoter (**D**) and mRNA (**E**) of *MYST1* in fibroblasts upon treatment with the SMAD3 inhibitor SIS3 (n = 4 biological replicates per group). **F-G: Bleomycin-induced pulmonary fibrosis:** mRNA levels of *Myst1* (**F**; n = 5 biological replicates for control and n = 4 for bleomycin group) and representative Immunofluorescence staining of MYST1 (green) in combination with DAPI (blue) and vimentin (red) at 400 fold magnification in murine lungs (**G**; n = 7 biological replicates per group). **H:** Knockdown of *MYST1* and overexpression of BECLIN1 in fibroblasts. mRNA levels of *COL1A1* (n = 6 biological replicates for groups overexpressing BECLIN1 and n = 5 for other groups) , *ATG7*, *BECLIN1* (n = 6 biological replicates per group) and secreted collagen protein (n = 4 biological replicates per group). All data are presented as median ± IQR. *p*-values were determined by two-sided Mann-Whitney test (**A**, **B**, **C, F, G**) or by ANOVA one-way with Tukey's multiple comparison post hoc test and are indicated in the figure (**D**, **E**, **H**). See source data for more detailed information. Horizontal scale bars, 50 µm. fluo.: fluorescence, int.: intensity, rel.: relative, prom.: promoter.

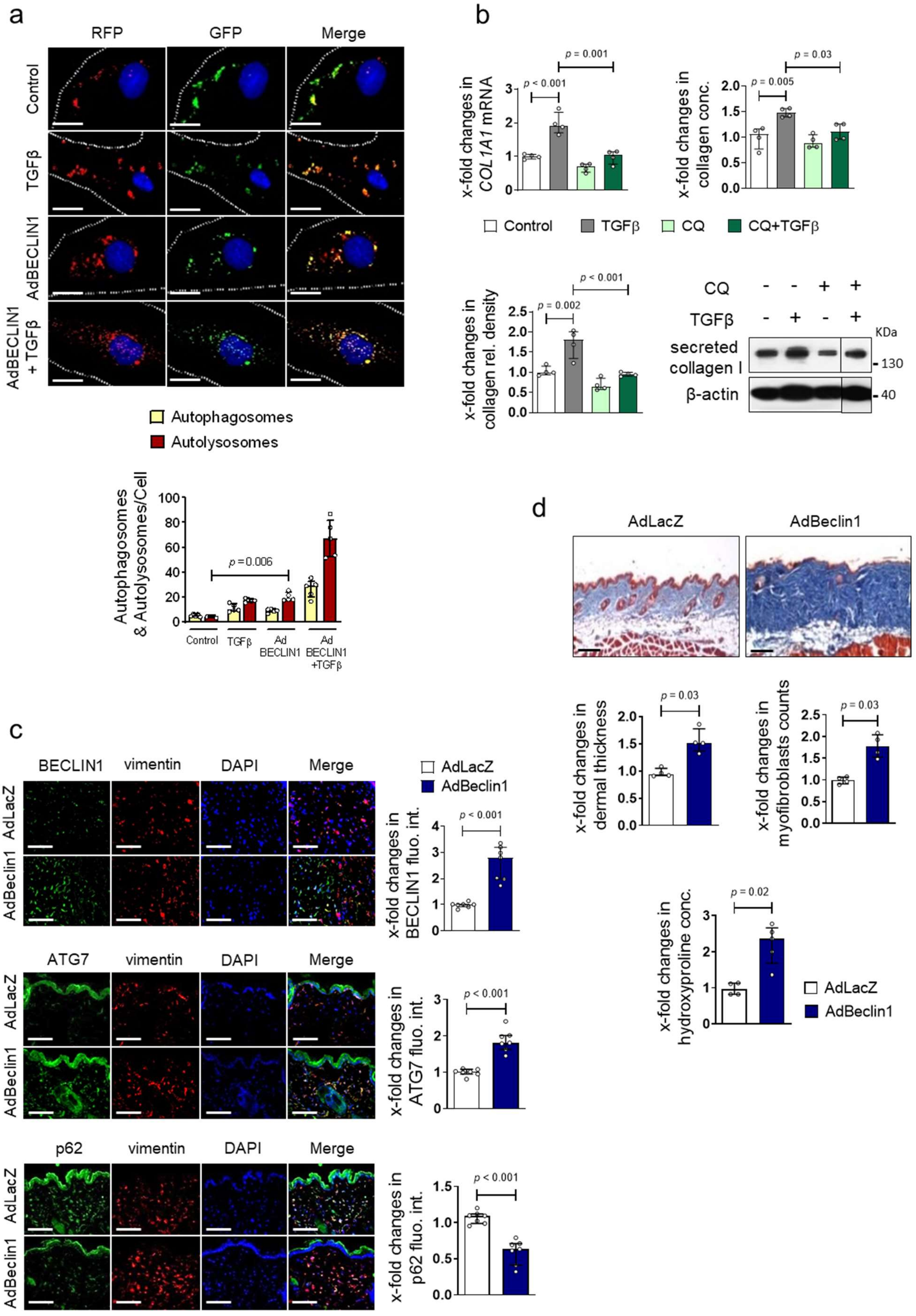
a
RFP
GFP
Merge
Control
TGFβ
AdBECLIN1
AdBECLIN1 + TGFβ
Autophagosomes
Autolysosomes
Autophagosomes & Autolysosomes/Cell
p = 0.006
Control
TGFβ
Ad BECLIN1
Ad BECLIN1 +TGFβ
b
x-fold changes in COL1A1 mRNA
p < 0.001
p = 0.001
x-fold changes in collagen conc.
p = 0.005
p = 0.03
Control
TGFβ
CQ
CQ+TGFβ
x-fold changes in collagen rel. density
p = 0.002
p < 0.001
CQ
TGFβ
KDa
secreted collagen I
β-actin
130
40
c
BECLIN1
vimentin
DAPI
Merge
AdLacZ
AdBeclin1
x-fold changes in BECLIN1 fluo. int.
p < 0.001
ATG7
x-fold changes in ATG7 fluo. int.
p62
x-fold changes in p62 fluo. int.
d
AdLacZ
AdBeclin1
x-fold changes in dermal thickness
p = 0.03
x-fold changes in myofibroblasts counts
x-fold changes in hydroxyproline conc.
p = 0.02

**Supplementary Figure 8: Modulation of autophagy by overexpression of BECLIN1 or by the autophagy inhibitor chloroquine regulates fibroblast activation and tissue fibrosis. A: Effects of TGFβ and overexpression of BECLIN1 on EGFP/RFP-LC3 reporter activity**; representative images and quantification (n = 5 biological replicates per group). Horizontal scale bars, 5 µm. **B:** Regulation of fibroblast activation by chloroquine (CQ): mRNA levels of *COL1A1*, levels of total collagen and levels of secreted type I collagen (n = 4 biological replicates per group) as analyzed by qRT-PCR, hydroxyproline assay and Western blot, respectively. Horizontal scale bar, 5 µm. **C-D: Adenoviral overexpression of BECLIN1 in murine skin. C:** Representative immunofluorescence staining of markers of autophagy BECLIN1, ATG7 or p62 (all green) in combination with DAPI (blue) and vimentin (red) at 400-fold magnification (n = 7 biological replicates per group). **D:** Representative trichrome stainings, dermal thickening, myofibroblast counts (n = 4 biological replicates per group) and hydroxyproline content (n = 4 biological replicates for control and n = 5 for Adbeclin1 group). Horizontal scale bars, 100 µm. Western blot samples in panel **B** were run on the same gel. Images were cropped at the lines only for the purpose of this figure. All data are presented as median ± IQR. *p*-values were determined by two-sided Mann-Whitney test (**C, D**) or by ANOVA one-way with Tukey's multiple comparison post hoc test (**A**, **B**) and are indicated in the figure. See source data for more detailed information. conc.: concentration, rel.: relative, fluo.: fluorescence, int.: intensity, CQ: chloroquine. The results in panel A are part of a large set of experiments and share the controls with figure 4c. Data are presented separately just for the purpose of this figure.

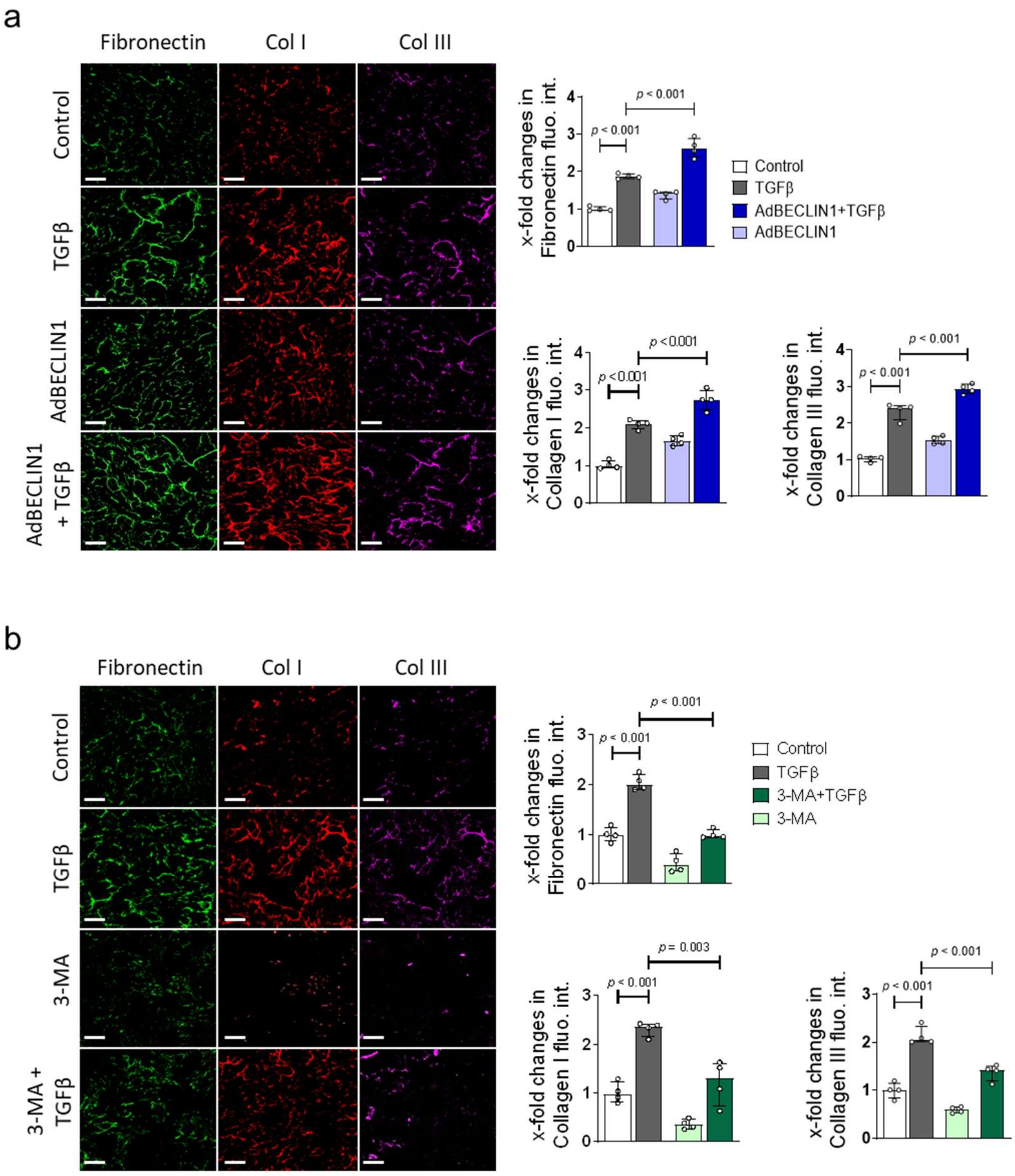


**Supplementary Figure 9: Autophagy-dependent regulation of extracellular matrix deposition. A:** Extracellular matrix fluorescence staining of human fibroblasts overexpressing BECLIN. Representative immunofluorescence staining of fibronectin (green), collagen I (red) and collagen III (magenta; n = 4 biological replicates per group) and respective quantifications. **B**: Extracellular matrix staining of human fibroblats treated with the autophagy inhibitor 3-methyladenine (3-MA). Representative images and quantifications of deposited fibronectin (green), collagen I (red) and collagen III (magenta; n = 4 biological replicates per group). Horizontal scale bars, 50 µm. All data are presented as median ± IQR. *p*-values were determined by ANOVA one-way with Tukey's multiple comparison post hoc test (**A**, **B**) and are indicated in the figure. See source data for more detailed information. Ad: Adenovirus, Fluo.: fluorescence, Int.: Intensity, Col I: collagen I, Col III: collagen III.

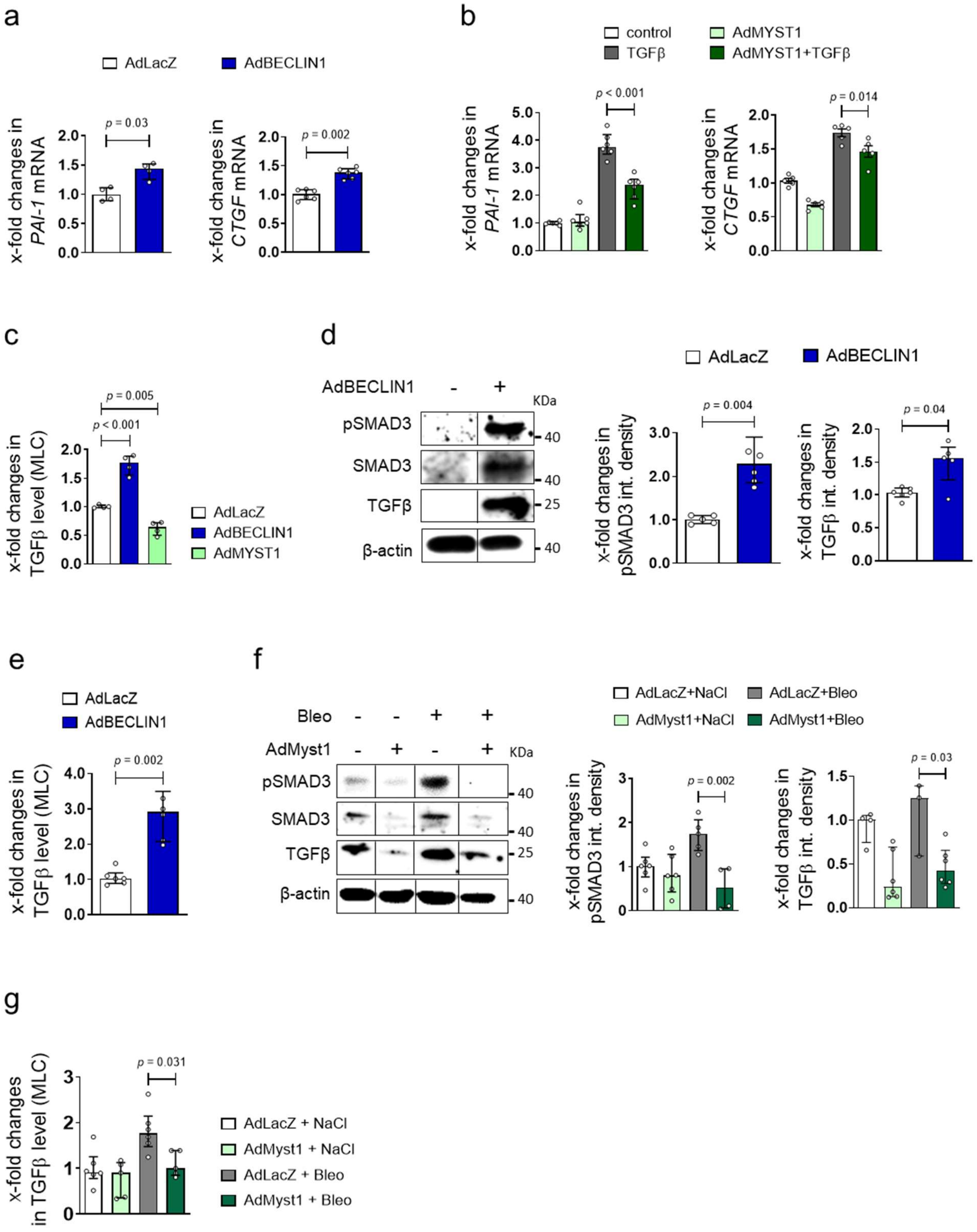

a
AdLacZ
AdBECLIN1
x-fold changes in PAI-1 mRNA
p = 0.03
x-fold changes in CTGF mRNA
p = 0.002
b
control
TGFβ
AdMYST1
AdMYST1+TGFβ
x-fold changes in PAI-1 mRNA
p < 0.001
x-fold changes in CTGF mRNA
p = 0.014
c
x-fold changes in TGFβ level (MLC)
p = 0.005
p < 0.001
AdLacZ
AdBECLIN1
AdMYST1
d
AdBECLIN1
pSMAD3
SMAD3
TGFβ
β-actin
KDa
x-fold changes in pSMAD3 int. density
p = 0.004
x-fold changes in TGFβ int. density
p = 0.04
e
AdLacZ
AdBECLIN1
x-fold changes in TGFβ level (MLC)
p = 0.002
f
Bleo
AdMyst1
KDa
pSMAD3
SMAD3
TGFβ
β-actin
AdLacZ+NaCl
AdLacZ+Bleo
AdMyst1+NaCl
AdMyst1+Bleo
x-fold changes in pSMAD3 int. density
p = 0.002
x-fold changes in TGFβ int. density
p = 0.03
g
x-fold changes in TGFβ level (MLC)
p = 0.031
AdLacZ + NaCl
AdMyst1 + NaCl
AdLacZ + Bleo
AdMyst1 + Bleo

**Supplementary Figure 10: Autophagy and MYST1 regulate TGFβ / SMAD signaling. A-C Human dermal fibroblasts:** Changes in the mRNA levels of the prototypical TGFβ / SMAD target genes *PAI-1* (n = 4 biological replicates per group) and *CTGF* (n = 6 biological replicates per group) induced by overexpression of BECLIN1 in cultured human dermal fibroblasts. **B:** Effects of MYST1 overexpression on *PAI-1* (n = 6 biological replicates) and *CTGF*, (n = 5 biological replicates per group) mRNA levels in fibroblasts stimulated with TGFβ. **C:** Changes in the levels of active TGFβ released from human dermal fibroblasts upon overexpression of BECLIN1 or MYST1, respectively, as measured by mink lung cell assays (n = 4 biological replicates per group). **D-G: Murine skin:** Levels of pSMAD3 (n = 5 biological replicates for control and n = 6 for AdBECLIN1 group), of total TGFβ (**D**; n = 6 biological replicates for control and n = 5 for AdBECLIN1 group) and of active TGFβ in murine skin (**E**; n = 6 biological replicates per group) upon overexpression of BECLIN1. Effects of the overexpresssion of MYST1 on bleomycin-induced accumulation of pSMAD3 (n = 5 biological replicates for bleomycin, n = 4 for AdMyst1+Bleo group and n = 6 for other groups) of total TGFβ (**F;** n = 4 biological replicates for control, n = 3 for bleomycin group and n = 6 for other groups), and of active TGFβ (**G**) in mice challenged with bleomycin (n = 5 biological replicates for groups overexpressing MYST1 and n = 6 for other groups). Western blot samples in panel **D** and **F** were run on the same gel. Images were cropped at the lines only for the purpose of this figure. All data are presented as median ± IQR . *p*-values were determined two-sided Mann-Whitney test (**A, D, E**) or by ANOVA one-way with Tukey's multiple comparison post hoc test (**B, C, F, G**) and are indicated in the figure. See source data for more detailed information. Ad: Adenovirus, rel.: relative, Bleo: bleomycin, Int.: Intensity.

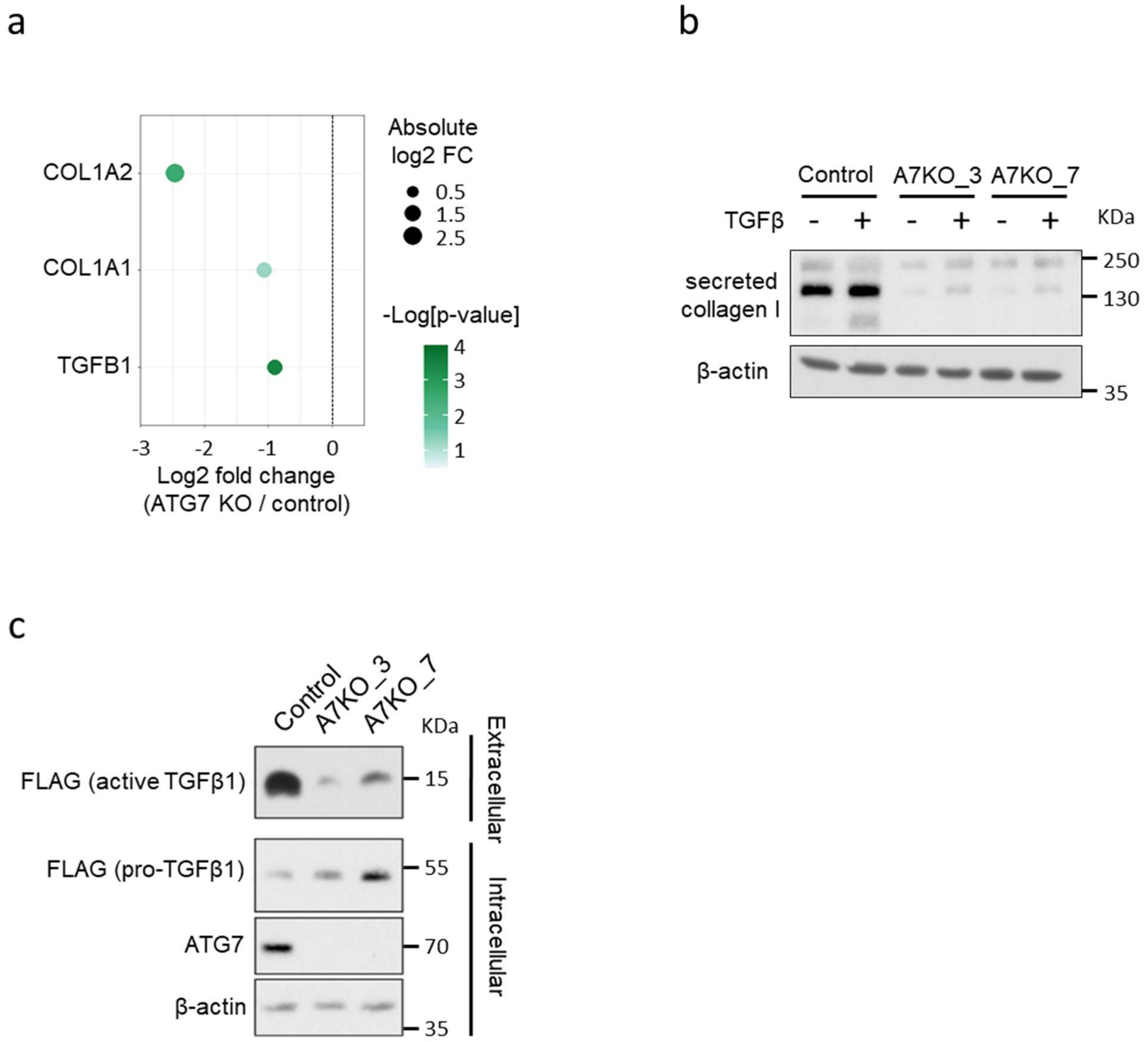


**Supplementary Figure 11: *ATG7* stable knockout in human WI26/SV-40 fibroblasts ameliorates collagen release. A**: Analysis of extracellular levels of COL1A1, COL1A2, and TGFβ1 using SILAC-based quantitative proteomics. COL1A2: -log [*p*-value] = 2,453; COL1A1: -log [*p*-value] = 1,233; TGFβ1: -log [*p*-value] = 3,458 (n = 4 biological replicates). **B**: Representative Western blot images of secreted collagen type I in *ATG7* knockout Wi-26 cell lines (n = 2 biological replicates). **C**: Representative Western blot images of intra- and extracellular TGFβ levels in *ATG7* knockout fibroblasts transfected with FLAG-(5A) TGFβ1 construct lines (n = 2 biological replicates). The SILAC data are presented as log2 fold changes (log2 FC) in *ATG7* knockout compared to control cells. The -log [*p*-values] were determined by one-sample t-test (Value = 0, S0 = 0.1, side = both). Control: Cells transfected with empty CRISPR/Cas9 vector, A7KO_3 and A7KO_7: *ATG7* knockout clones 3 and 7, FC: fold change. See source data for more detailed information.

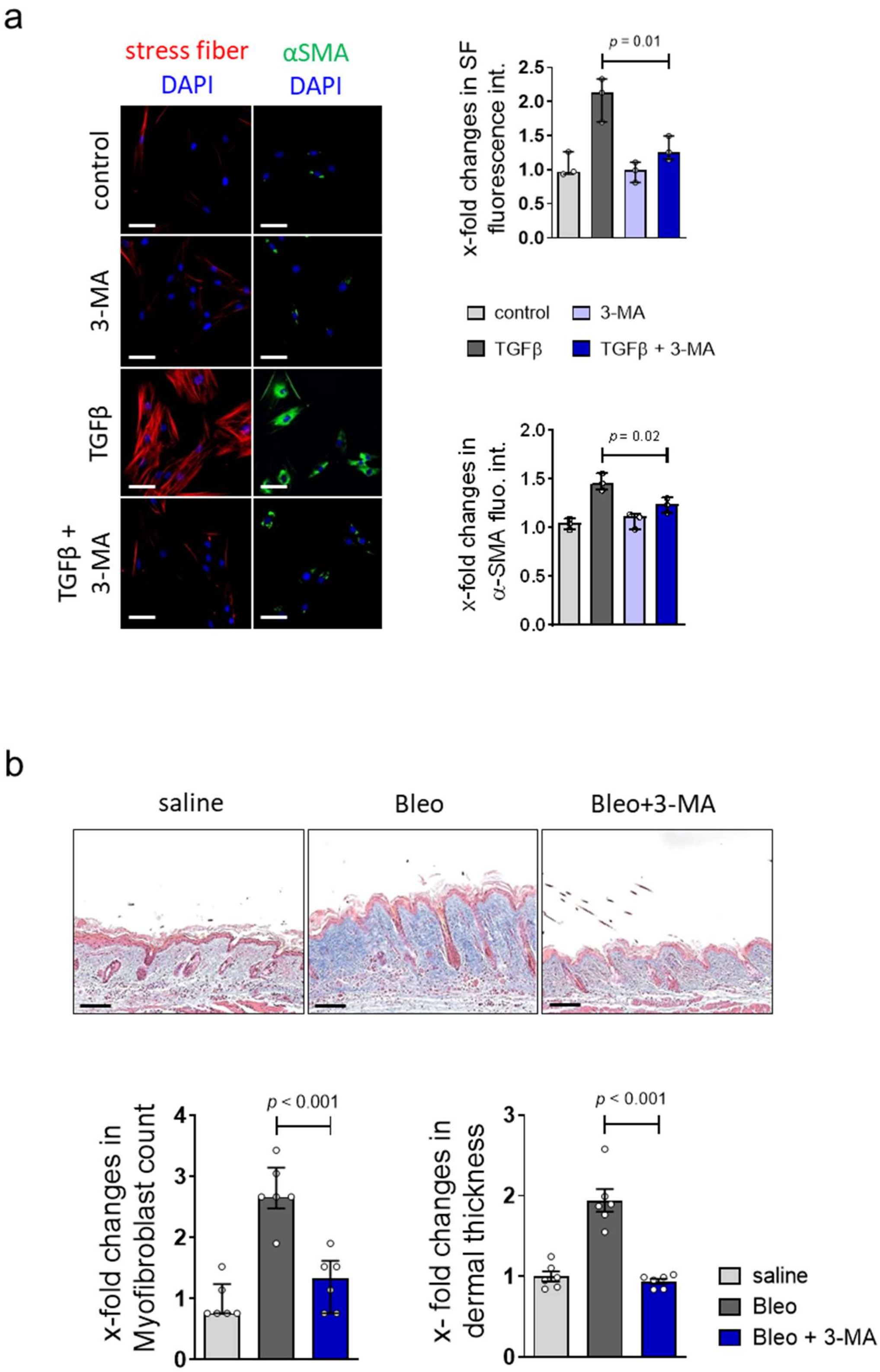
a
stress fiber
DAPI
αSMA
DAPI
control
3-MA
TGFβ
TGFβ + 3-MA
x-fold changes in SF fluorescence int.
p = 0.01
2.5
2.0
1.5
1.0
0.5
0.0
control
3-MA
TGFβ
TGFβ + 3-MA
x-fold changes in α-SMA fluo. int.
p = 0.02
2.0
1.5
1.0
0.5
0.0
b
saline
Bleo
Bleo+3-MA
x-fold changes in Myofibroblast count
p < 0.001
4
3
2
1
0
x- fold changes in dermal thickness
p < 0.001
3
2
1
0
saline
Bleo
Bleo + 3-MA

**Supplementary Figure 12: Autophagy inhibition by 3-MA reduces myofibroblast differentiation and ameliorates bleomycin-induced skin fibrosis. A: Cultured human fibroblasts.** Fibroblasts treated with with 3-methyladenine (3-MA) in the presence or absence of TGFβ. Representative images and quantifications of stress fibers (red) and αSMA (green; n = 3 biological replicates per group) costained with DAPI (blue). Horizontal scale bars, 50 µm. **B: Bleomycin-induced skin fibrosis**. Representative trichrome stainings, myofibroblast counts and dermal thickening (n = 6 biological replicates per group). Horizontal scale bars, 100 µm. All data are presented as median ± IQR. *p*-values were determined by ANOVA one-way with Tukey's multiple comparison post hoc test and are indicated in the figure. See source data for more detailed information. Bleo: bleomycin, int.: intensity, SF: stress fibers.

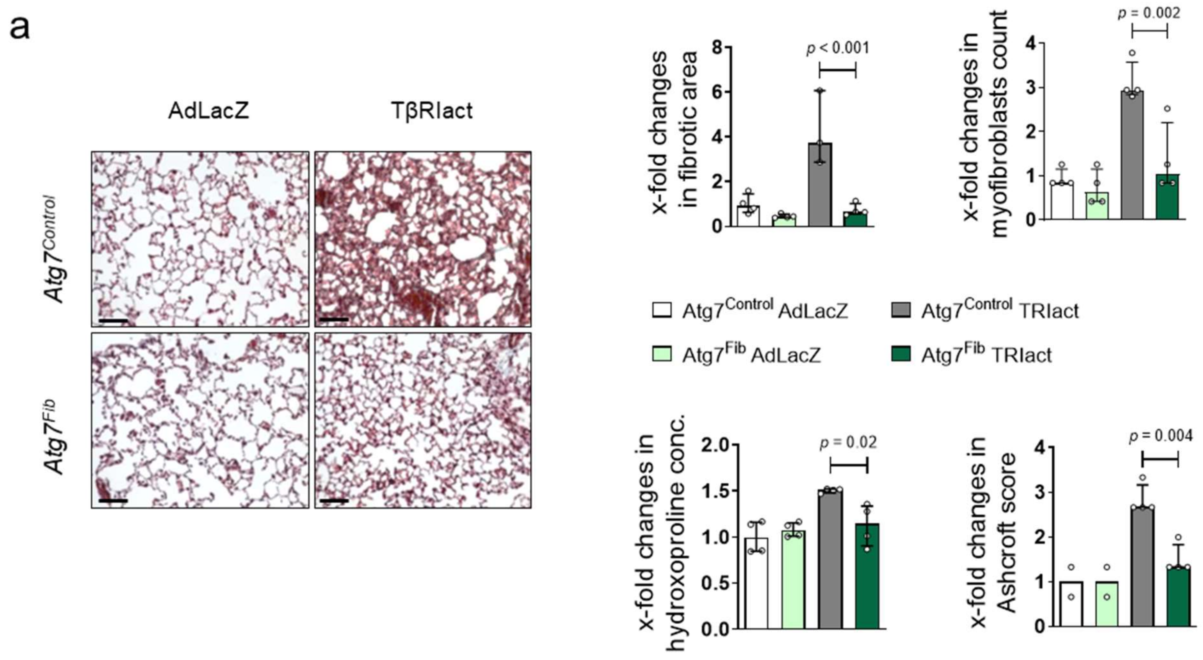


**Supplementary Figure 13: Fibroblast-specific knockout of ATG7 ameliorates TβRIact-induced pulmonary fibrosis.** Representative trichrome stainings shown, fibrotic area (n = 3 biological replicates for Atg7$^{Control}$ TβRIact, n = 4 for the other groups), myofibroblast counts (n= 4 biological replicates per group), hydroxyproline content (n = 4 biological replicates per group) and Ashcroft scores (n = 2 biological replicates for Atg7$^{Control}$ AdLacZ and Atg7$^{Fib}$ AdLacZ and n = 4 for other groups). Horizontal scale bar, 100 µm. All data are presented as median ± IQR. *p*-values were determined by ANOVA one-way with Tukey's multiple comparison post hoc test and are indicated in the figure. See source data for more detailed information. Ad: Adenovirus, TβRIact: constitutively active TGFβ receptor type I, conc.: concentration. *Atg7*$^{control}$: littermate control mice *Atg7*$^{fib}$: fibroblast-specific *Atg7* knockout mice.

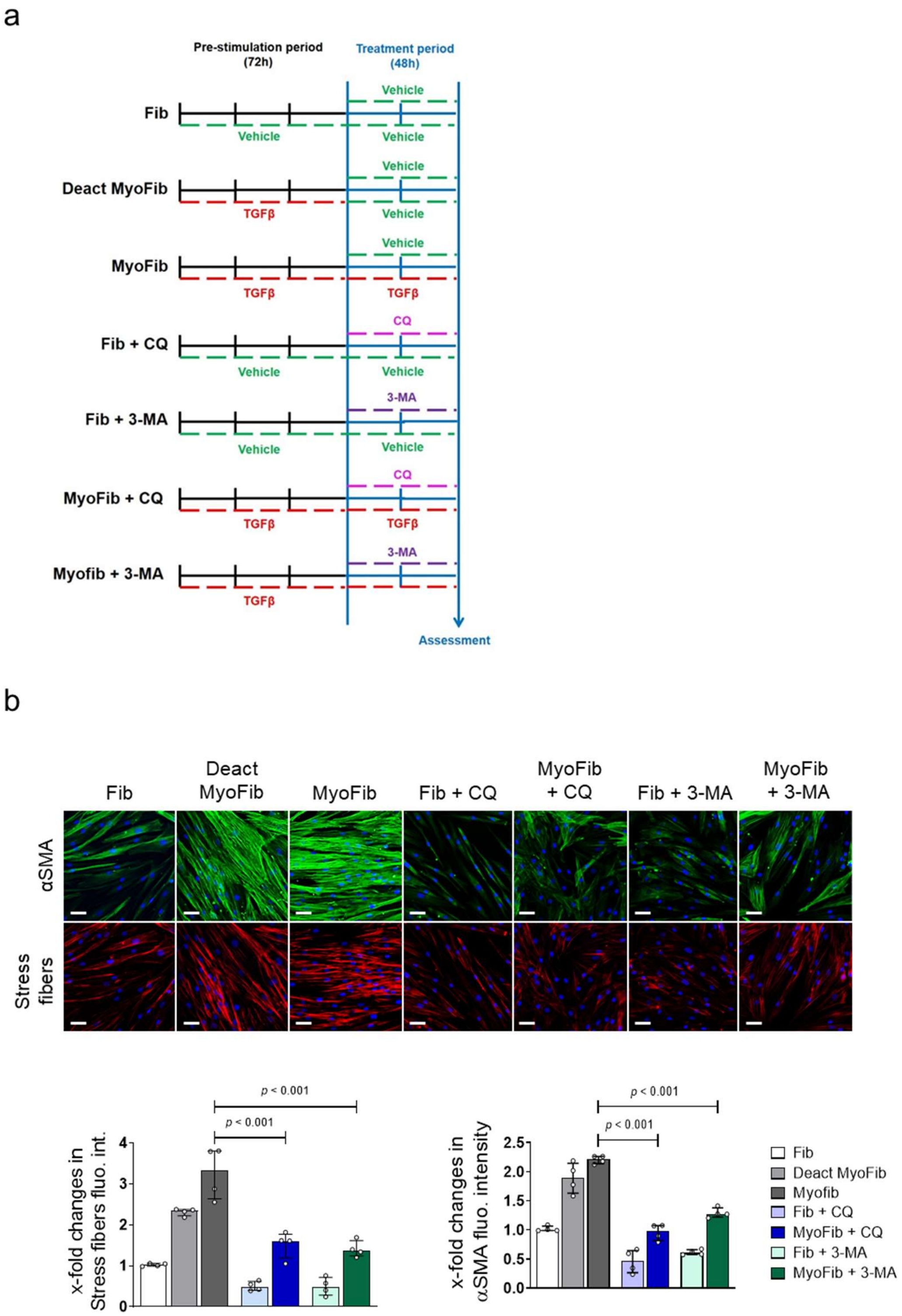


**Supplementary Figure 14: Inhibition of autophagy can induce re-differentiation of myofibroblasts into fibroblasts. A:** Schematic illustration of the experimental procedure. **B:** Representative images and quantification of αSMA (green) and stress fibers (red; n = 4 biological replicates) costained with DAPI (blue). Horizontal scale bars, 30 µm. All data are presented as median ± IQR. *p*-values were determined by ANOVA one-way with Tukey's multiple comparison post hoc test and are indicated in the figure. See source data for more detailed information. Fluo.: fluorescence, Fib: fibroblast, MyoFib: myofibroblast, deact MyoFib: deactivated myofibroblast, CQ: chloroquine.

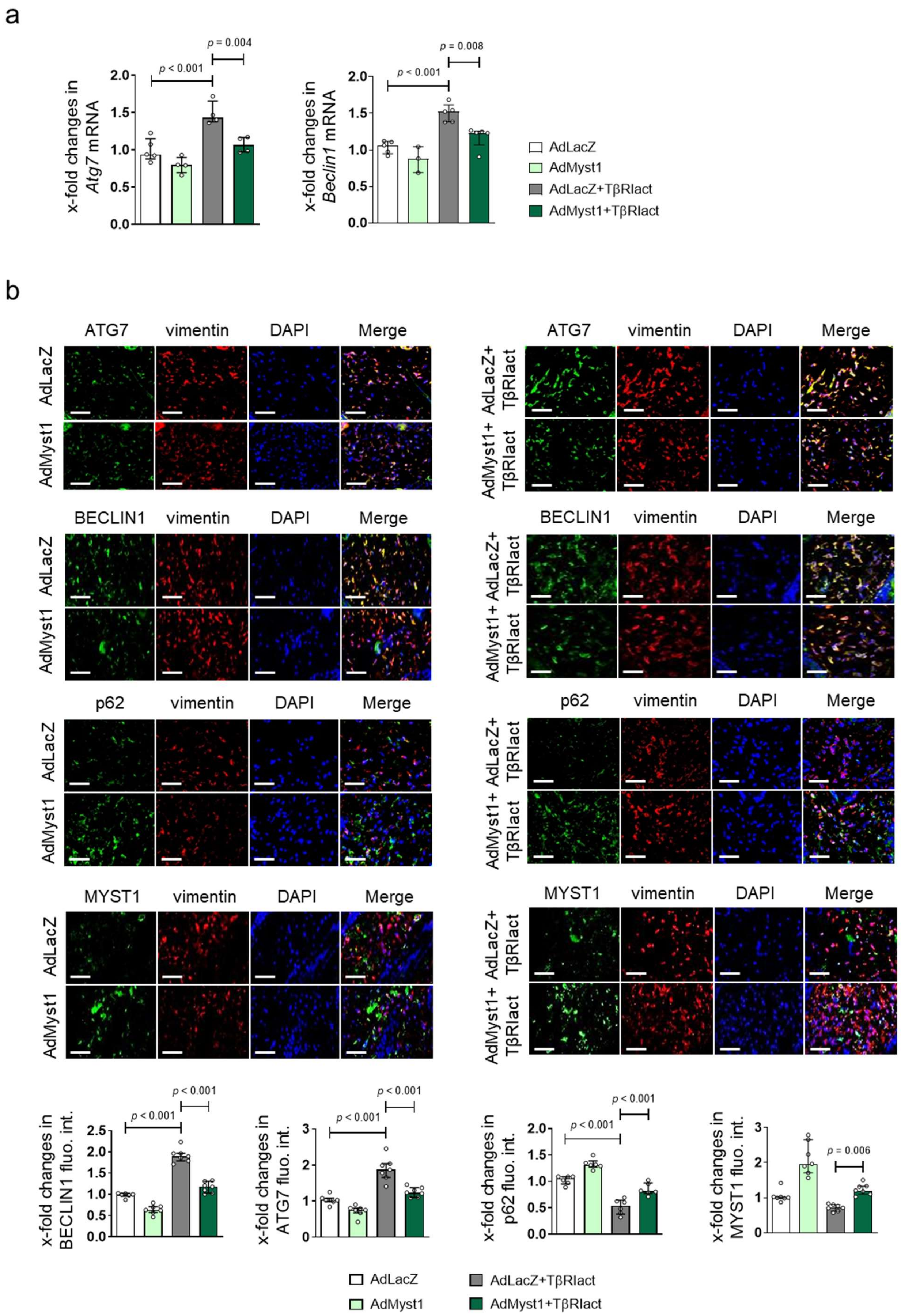
a
x-fold changes in Atg7 mRNA
x-fold changes in Beclin1 mRNA
p < 0.001
p = 0.004
p = 0.008
AdLacZ
AdMyst1
AdLacZ+TβRIact
AdMyst1+TβRIact
b
ATG7
BECLIN1
p62
MYST1
vimentin
DAPI
Merge
AdLacZ
AdMyst1
AdLacZ+ TβRIact
AdMyst1+ TβRIact
x-fold changes in BECLIN1 fluo. int.
x-fold changes in ATG7 fluo. int.
x-fold changes in p62 fluo. int.
x-fold changes in MYST1 fluo. int.
p = 0.006

**Supplementary Figure 15: Overexpression of MYST1 prevents the activation of autophagy upon overexpression of TβRIact in murine skin. A:** mRNA levels of *Atg7* (n = 5 biological replicates for AdLacZ group and n = 4 for other groups) and *Beclin1* (n = 3 biological replicates for AdMyst1 group and n = 5 for other groups). **B:** Representative immunofluorescence staining for ATG7 (n = 7 biological replicates), BECLIN1 (n = 6 biological replicates for control and n = 7 for other groups), p62 (n = 6 biological replicates per group) and MYST1 (all green; n = 7 biological replicates per group) in combination with DAPI (blue) and vimentin (red) and respective quantification. Horizontal scale bar, 50 µm. All data are presented as median ± IQR. *p*-values were determined by ANOVA one-way with Tukey's multiple comparison post hoc test and are indicated in the figure. See source data for more detailed information. Ad: adenovirus, TβRIact: constitutively active TGFβ receptor type I, rel.: relative, fluo.: fluorescence, int.: intensity.

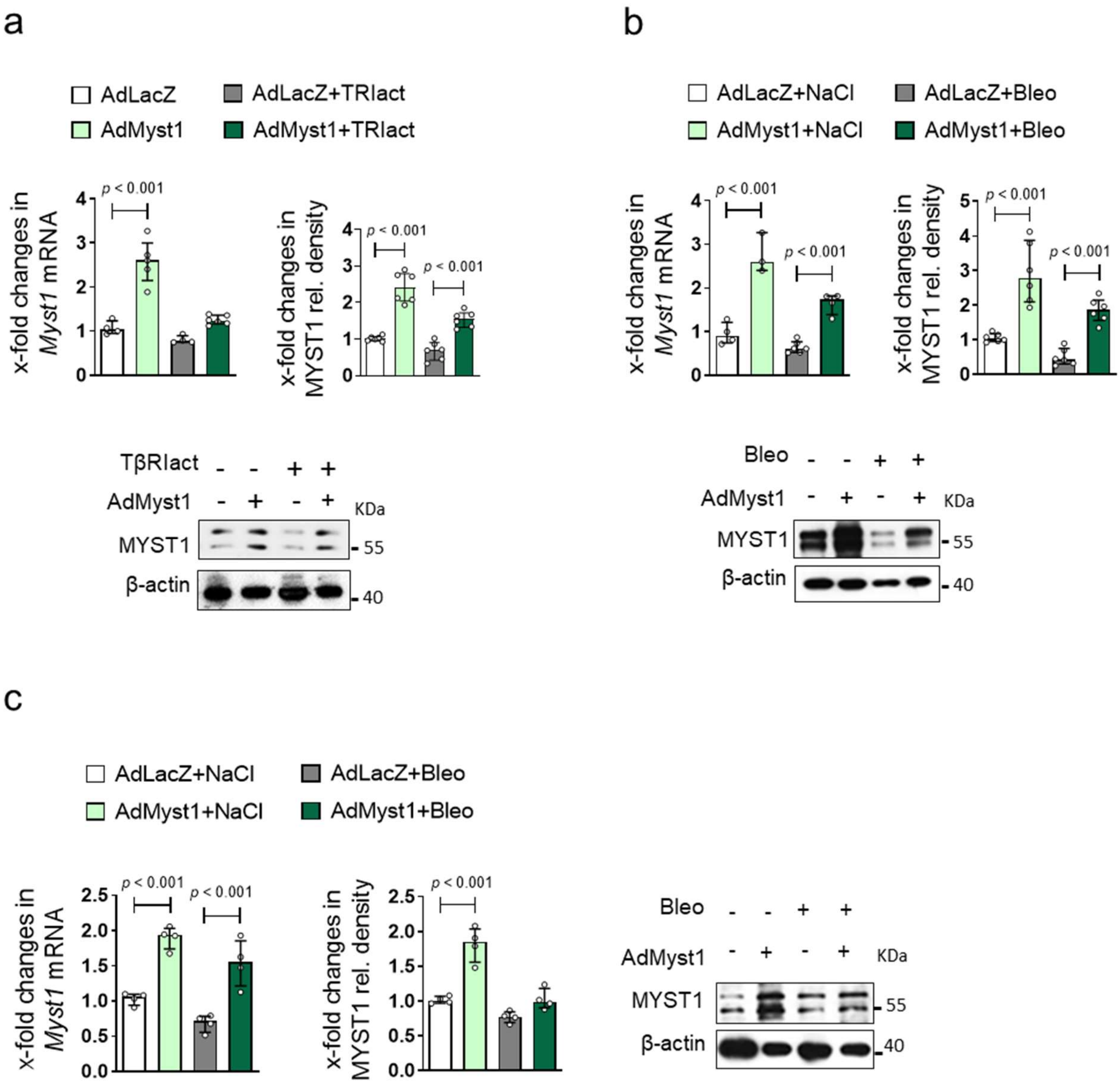


**Supplementary Figure 16: Adenoviral overexpression of MYST1 in murine models of fibrosis. A: TβRIact-induced skin fibrosis**: mRNA (n = 5 biological replicates for AdMyst1 group, n = 6 for TβRIact group overexpressing MYST1 and n = 4 for other groups) and protein levels of MYST1 (n = 6 biological replicates). **B: Bleomycin-induced pulmonary fibrosis**: mRNA (n = 3 biological replicates for AdMyst1 group, n = 6 for bleomycin group and n = 4 for other groups) and protein levels of MYST1 (n = 6 biological replicates). **C: Bleomycin-induced dermal fibrosis**: mRNA and protein levels of MYST1 (n = 4 biological replicates). All data are presented as median ± IQR. *p*-values were determined by ANOVA one-way with Tukey's multiple comparison post hoc test and are indicated in the figure. See source data for more detailed information. Ad: adenovirus, TβRIact: constitutively active TGFβ receptor type I, rel.: relative, Bleo: bleomycin.

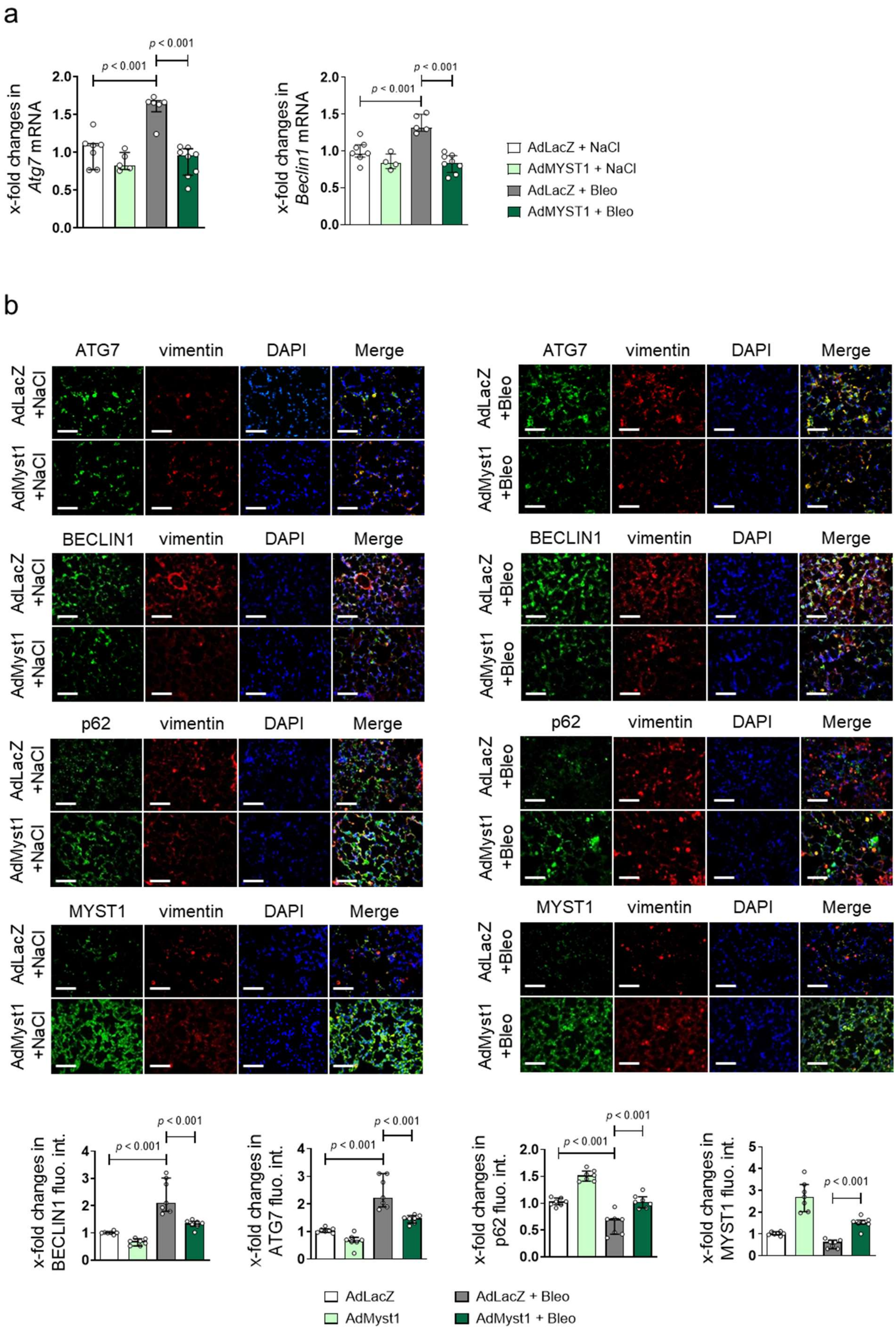
a
p < 0.001
p < 0.001
x-fold changes in Atg7 mRNA
2.0
1.5
1.0
0.5
0.0
p < 0.001
p < 0.001
x-fold changes in Beclin1 mRNA
2.0
1.5
1.0
0.5
0.0
AdLacZ + NaCl
AdMYST1 + NaCl
AdLacZ + Bleo
AdMYST1 + Bleo
b
ATG7
vimentin
DAPI
Merge
AdLacZ +NaCl
AdMyst1 +NaCl
AdLacZ +Bleo
AdMyst1 +Bleo
BECLIN1
p62
MYST1
x-fold changes in BECLIN1 fluo. int.
x-fold changes in ATG7 fluo. int.
x-fold changes in p62 fluo. int.
x-fold changes in MYST1 fluo. int.
p < 0.001
AdLacZ
AdMyst1
AdLacZ + Bleo
AdMyst1 + Bleo

**Supplementary Figure 17: Overexpression of MYST1 prevents the activation of autophagy in bleomycin-induced pulmonary fibrosis. A:** mRNA levels of *Atg7* (n = 7 biological replicates for control, n = 5 for AdMyst1, n = 6 for bleomycin and n = 8 for AdMyst1 + bleomycin group) and *Beclin1* (n = 7 biological replicates for control, n = 4 for AdMyst1 group, n = 5 for bleomycin group and n = 8 for AdMyst1 + bleomycin group). **B:** Representative immunofluorescence staining for ATG7, BECLIN1, p62 and MYST1 (all green) in combination with DAPI (blue) and vimentin (red) and respective quantification (n = 7 biological replicates per group for all readouts). Horizontal scale bar, 50 µm. All data are presented as median ± IQR. *p*-values were determined by ANOVA one-way with Tukey's multiple comparison post hoc test and are indicated in the figure. See source data for more detailed information. Ad: adenovirus, Bleo: bleomycin, rel.: relative, fluo.: fluorescence, int.: intensity.

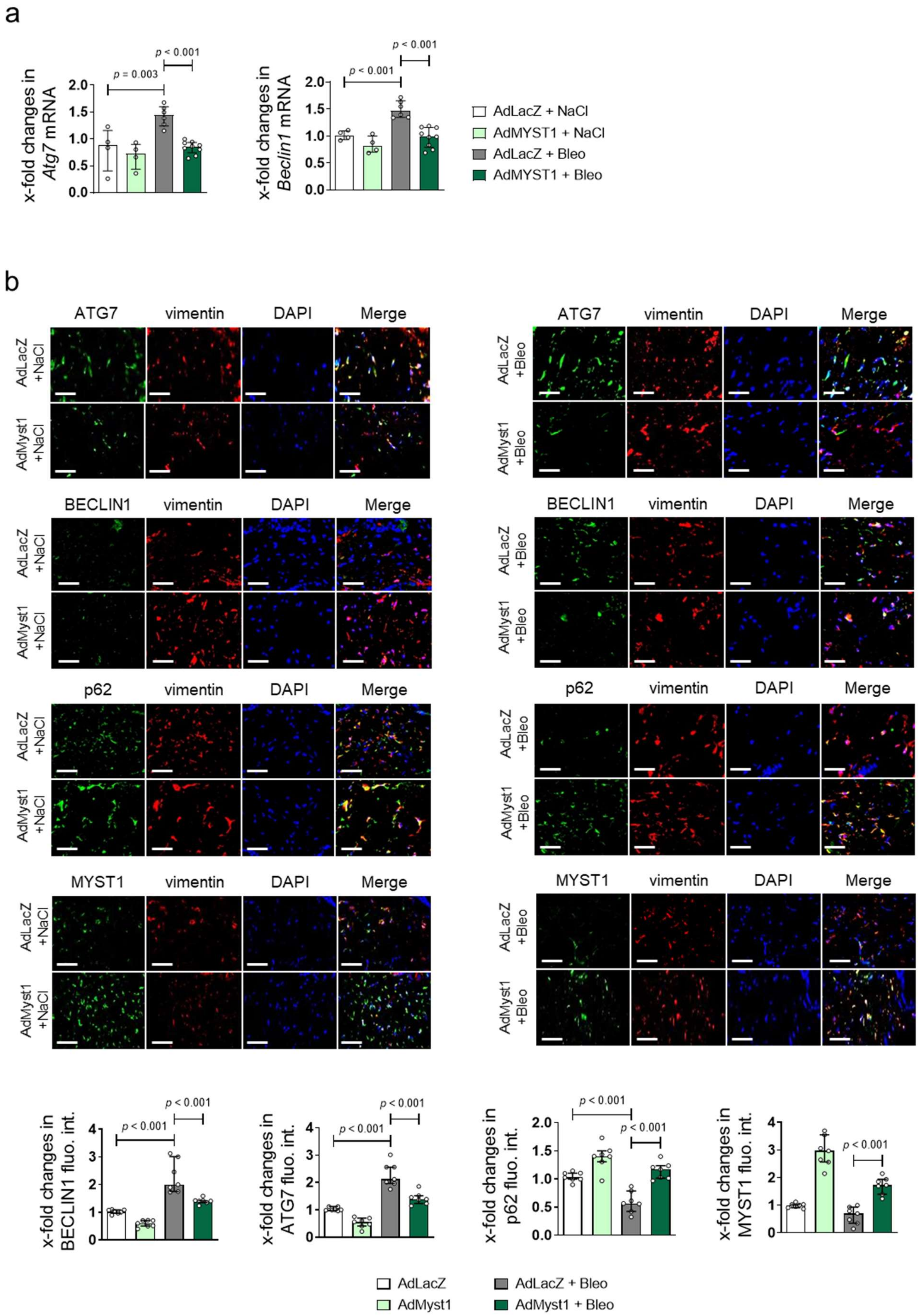

a
x-fold changes in Atg7 mRNA
2.0
1.5
1.0
0.5
0.0
p = 0.003
p < 0.001
x-fold changes in Beclin1 mRNA
p < 0.001
p < 0.001
AdLacZ + NaCl
AdMYST1 + NaCl
AdLacZ + Bleo
AdMYST1 + Bleo
b
ATG7
vimentin
DAPI
Merge
AdLacZ +NaCl
AdMyst1 +NaCl
AdLacZ +Bleo
AdMyst1 +Bleo
BECLIN1
p62
MYST1
x-fold changes in BECLIN1 fluo. int.
x-fold changes in ATG7 fluo. int.
x-fold changes in p62 fluo. int.
x-fold changes in MYST1 fluo. int.
p < 0.001
AdLacZ
AdMyst1
AdLacZ + Bleo
AdMyst1 + Bleo

**Supplementary Figure 18: Overexpression of MYST1 prevents the activation of autophagy in bleomycin-induced dermal fibrosis. A:** mRNA levels of *Atg7* and *Beclin1* (n = 4 biological replicates for NaCl treated groups, n = 6 for bleomycin and n = 9 for AdMyst1 + bleomycin). **B:** Representative immunofluorescence staining for ATG7, BECLIN1, and p62 (all green) in combination with DAPI (blue) and vimentin (red) and respective quantification (n = 7 biological replicates per group for all readouts). Horizontal scale bar, 50 µm. All data are presented as median ± IQR. *p*-values were determined by ANOVA one-way with Tukey's multiple comparison post hoc test and are indicated in the figure. See source data for more detailed information. Ad: adenovirus, Bleo: bleomycin, rel.: relative, fluo.: fluorescence, int.: intensity.

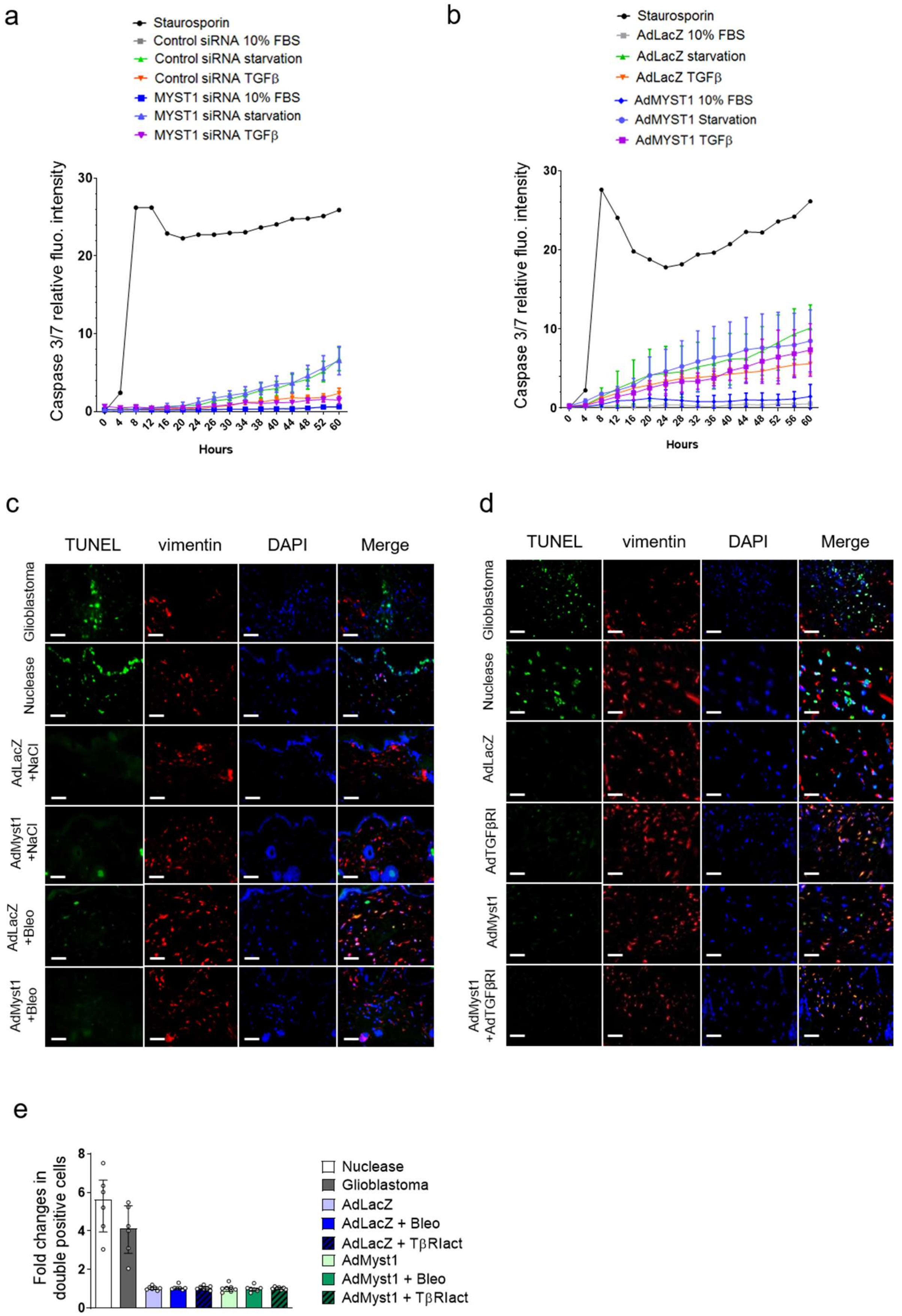
a
Staurosporin
Control siRNA 10% FBS
Control siRNA starvation
Control siRNA TGFβ
MYST1 siRNA 10% FBS
MYST1 siRNA starvation
MYST1 siRNA TGFβ
Caspase 3/7 relative fluo. intensity
Hours
b
Staurosporin
AdLacZ 10% FBS
AdLacZ starvation
AdLacZ TGFβ
AdMYST1 10% FBS
AdMYST1 Starvation
AdMYST1 TGFβ
Caspase 3/7 relative fluo. intensity
Hours
c
TUNEL
vimentin
DAPI
Merge
Glioblastoma
Nuclease
AdLacZ +NaCl
AdMyst1 +NaCl
AdLacZ +Bleo
AdMyst1 +Bleo
d
TUNEL
vimentin
DAPI
Merge
Glioblastoma
Nuclease
AdLacZ
AdTGFβRI
AdMyst1
AdMyst1 +AdTGFβRI
e
Fold changes in double positive cells
Nuclease
Glioblastoma
AdLacZ
AdLacZ + Bleo
AdLacZ + TβRIact
AdMyst1
AdMyst1 + Bleo
AdMyst1 + TβRIact

**Supplementary Figure 19: Modulation of MYST1 expression does not induce apoptosis in fibroblasts. A-B:** Quantification of the activity of Caspases 3 and 7 in fibroblasts with siRNA-mediated knockdown (**A**) or overexpression of MYST1 (**B;** n = 2 biological replicates for staurosporin group and n = 4 for other groups, for all readouts) **C-E:** TUNEL staining of skin sections from mice with or without overexpression of MYST1. **C:** Representative images of sections of bleomycin-induced skin fibrosis costained with vimentin and DAPI. **D:** Representative images of sections of TβRIact-induced skin fibrosis costained with vimentin and DAPI. Horizontal scale bars, 50 µm. **E:** Quantification of TUNEL-positive apoptotic fibroblast (n = 6 biological replicates for nuclease and glioblastoma group and n = 7 for other groups). Data are presented as mean or median ± IQR. See source data for more detailed information. Ad: adenovirus, Bleo: bleomycin, TβRIact: constitutively active TGFβ receptor type I, fluo: fluorescence.

**Supplementary Table 1:** List of primers used for the mutagenesis of SBEs in MYST1 promoter. Fwd: forward primer.

| **Primers** | **Sequence reaction 5'-3'** |
|---|---|
| SBE1 fwd | GGATCACGAGGTCAGAAGATCGGCGTTATTCTGGCTAACACGATGAAACTC |
| SBE2 fwd | CCCGGCCTTTTTATTTTTTTGAGATGGAACGCGGCTCTTGGTCCCCAGGC |
| SBE3 fwd | CGGTGTTAGCCAGGATGACGCCGATCTCCGCCCGCCTC |

**Supplementary Table 2:** Primers for qRT-PCR.

| **Human primers** | **Forward reaction 5'-3'** | **Reverse reaction 5'-3'** |
|---|---|---|
| *ATG7* | ATTGCTGCATCAAGAAACCC | GATGGAGAGCTCCTCAGCA |
| *BECLIN1* | TGGTAGTTCTGGAGGCCT | AGGCACTGTGGCCTCGGG |
| *β-ACTIN* | AGAAAATCTGGCACCACACC | TAGCACAGCCTGGATAGCAA |
| *COL1A1* | ACGAAGACATCCCACCAATC | ATGGTACCTGAGGCCGTTC |
| *MYST1* | ACCTCAAAAGTGCCCAGTATAAGA | GACGGAGTCCACTGTGATGG |
| *CTGF* | AACTCACACAACAACTCTTCCCCGC | GAGTCGCACTGGCTGTCTCCTCT |
| *PAI-1* | TCATTGCTGCCCCTTATGA | GTTGGTGAGGGCAGAGAGAG |
| *P62* | TGCCCAGACTACGACTTGTG | AGTGTCCGTGTTTCACCTTCC |
| **Mouse primers** | **Forward reaction 5'-3'** | **Reverse reaction 5'-3'** |
| *Atg7* | TGCCTATGATGATCTGTGTC | CACCAACTGTTATCTTTGTCC |

| | | |
|---|---|---|
| *Beclin1* | GGCCAATAAGATGGGTCTGA | GCTGCACACAGTCCAGAAAA |
| *β-actin* | TCTTTGATGTCACGCACGAT | TACAGCTTCACCACCACA |
| *Col1a1* | GAAGCACGTCTGGTTTGGA | ACTCGAACGGGAATCCATC |
| *Myst1* | CGGCCGGATAGCACCTG | TTCACTCGAGACTGGATCACTTC |
| *P62* | GCTGCCCTATACCCACATCT | CGCCTTCATCCGAGAAAC |

**Supplementary Table 3:** List of antibodies and dyes used in this study**.** WB: Western blot, IF: Immunofluorescence staining, ECM: Extracellular Matrix staining, ChIP: Chromatin Immunoprecipitation, Cat.: Catalog number. N/A: not applicable.

| **Reagent type/ Aplication** | **Species** | **Designation** | **Source** (Catalog number, clone name, lot number) | **Dilution / Application** |
|---|---|---|---|---|
| Primary antibody | monoclonal mouse | αSMA (Alpha-smooth muscle) | Sigma-Aldrich / Cat. A5228, clone 1A4 or A2547 / clone 1A4 | both 1:1000 IF |
| Primary antibody | monoclonal mouse | β-actin | Sigma-Aldrich / Cat. A5441 / clone AC-15 | 1:10000 WB |
| Primary antibody | polyclonal rabbit | ATG7 | Abcam / Cat. ab133528 / clone EPR6251 | 1:1000 WB / 1:200 IF |
| Primary antibody | polyclonal rabbit | ATG7 | AnaSpec / Cat. AS-54230 | 1:1000 WB / 1:200 IF |
| Primary antibody | polyclonal rabbit | BECLIN1 | Abcam / Cat. ab62557 | 1:1000 WB / 1:200 IF |
| Primary antibody | polyclonal rabbit | Collagen type I (Col I) | Abcam / ab138492 / clone EPR7785 | 1:1000 WB |
| Primary antibody | polyclonal goat | Collagen type I (Col I) | Southern Biotech / Cat. 1310-01 | 1:2000 WB |
| Primary antibody | polyclonal rabbit | Collagen type I (Col I) | Merck Millipore / Cat. #AB745 | 1:100 ECM |
| Primary antibody | polyclonal rabbit | Collagen type III (Col III) | Merck Millipore / Cat. #AB747 | 1:100 ECM |

| | | | | |
|---|---|---|---|---|
| Primary antibody | monoclonal mouse | anti- Fibronectin antibody conjugated with Alexa Fluor 488 | eBiosciences / Cat. #53-9869-82 / clone FN-3 | 1:400 ECM |
| Primary antibody | polyclonal rabbit | FLAG tag | Proteintech / Cat. 80010-1-RR / clone 4K14 | 1:5000 WB |
| Primary antibody | polyclonal goat | GFP | Abcam / Cat. ab6673 | 1:200 IF |
| Primary antibody | polyclonal rabbit | Histone H3 | Cell Signaling / Cat. #9715 | 1:1000 WB |
| Primary antibody | polyclonal rabbit | H4K16ac (acetyl-Histone H4 (Lys16)) | Merck Millipore / Cat. #07-329/ Lot # 2506422 | 1:1000 WB |
| Primary antibody | monoclonal rat | LAMP2 | Abcam /Cat. ab13524 / clone GL2A7 | 1:200 IF |
| Primary antibody | polyclonal goat | LAP (pro-domain of TGFβ1) | R&D systems / Cat. AF-246-NA | 1:2000 WB |
| Primary antibody | polyclonal rabbit | LC3B / MAP1LC3B | Novus biologicals / Cat. NB 100-2220 | 1:1000 WB / 1:200 IF |
| Primary antibody | monoclonal mouse | MYST1 / MOF | Genetex / Cat. GTX83065 / clone 8C4C4 | 1: 1000 WB / 1:200 IF |
| Primary antibody | monoclonal mouse | MYST1 / MOF | Santa cruz biotechnology / Cat. Sc-271691 | 1: 1000 WB / 1:200 IF |
| Primary antibody | monoclonal rabbit | prolyl-4-hydroxylase-β (P4Hβ) | Acris antibodies / TA308403 | 1:200 IF |
| Primary antibody | monoclonal rabbit | pSMAD3 (SMAD3 phospho S423 + S425) | Abcam/ Cat. ab52903 / clone EP823Y | 1:1000 WB |
| Primary antibody | monoclonal rabbit | RFP | Abcam / Cat. ab62341 | 1:200 IF |
| Primary antibody | polyclonal rabbit | SMAD3 | Cell signaling / Cat. #9523S / clone C67H9 | 1:1000 WB / 1:50 ChIP |
| Primary antibody | monoclonal mouse | SQSTM1/p62 | Abcam / Cat. ab56416 | 1:2000 WB / 1:200 IF |

| | | | | |
|---|---|---|---|---|
| Primary antibody | polyclonal rabbit | TGFβ | Cell signaling / Cat. #3711S | 1:1000 WB |
| Primary antibody | monoclonal rabbit | Vimentin | Abcam / Cat. ab92547 / clone EPR3776 | 1: 200 IF |
| IgG control | Rabbit | Normal rabbit IgG | Santa Cruz Biotechnology / sc-2027 | IF and ChIP |
| IgG control | Mouse | Normal mouse IgG | Santa cruz biotechnology / sc-2025 | IF |
| Secondary antibody | Polyclonal Goat Anti-Mouse | HRP-conjugated | Dako / Cat. P044701-2 | 1:5000 WB / 1:500 IHC |
| Secondary antibody | Polyclonal Goat Anti-Rabbit | HRP-conjugated | Dako / Cat. P044801-2 | 1:5000 WB |
| Secondary antibody | Polyclonal Rabbit Anti-Goat | HRP-conjugated | Dako / Cat. P044901-2 | 1:5000 WB |
| Secondary antibody | Goat anti-Rabbit IgG | Alexa fluor 488 | Invitrogen / Cat. A-11008 | 1:200 IF / 1:250 ECM |
| Secondary antibody | Goat anti-Mouse IgG | Alexa fluor 488 | Invitrogen / Cat. A-11001 | 1:200 IF |
| Secondary antibody | Goat anti-Rat IgG | Alexa fluor 488 | Invitrogen / Cat. A-11006 | 1:200 IF |
| Secondary antibody | Donkey anti-Goat | Alexa fluor 488 | Invitrogen / Cat. AA32814 | 1:200 IF |
| Secondary antibody | Goat anti-Mouse IgG | Alexa fluor 555 | Invitrogen / Cat. A-21422 | 1:200 IF |
| Secondary antibody | Goat anti-Rabbit IgG | Alexa fluor 555 | Invitrogen / Cat. A-21428 | 1:200 IF |
| Secondary antibody | Goat anti-Rat IgG | Alexa fluor 555 | Invitrogen / Cat. A-21434 | 1:200 IF |
| Secondary antibody | Goat Anti-Rabbit | Alexa fluor 647 | Invitrogen / Cat. #A21244 | 1:200 IF / 1:250 ECM |
| Dye | N/A | DAPI | Santa Cruz Biotechnology / CAS 28718-90-3 | 1.5 µg / ml IF |

| | | | | |
|---|---|---|---|---|
| Dye | N/A | rhodamine-conjugated phalloidin | Sigma-Aldrich / Cat. 50556 | 1:250 IF |
| Dye | N/A | rhodamine-conjugated phalloidin | Thermo Fisher Scientific / Cat. R415 | 1:250 IF |

**Supplementary Table 4:** Settings and parameters used for the mass spectrometry analysis.

## HPLC gradients

| Experiment(s) | Gradient length | Duration (min) | Solvent B (80 % acetonitrile, 0.1% formic acid) |
|---|---|---|---|
| **Secretome** | 150 min | 5 | 5 to 8% |
| | | 119 | 8 to 29% |
| | | 5 | 29 to 68% |
| | | 5 | 68 to 95% |
| | | 6 | 95% |

## MS settings

| | MS1 | | | | | |
|---|---|---|---|---|---|---|
| **Experiment(s)** | **Resolution** | **AGC Target** | **Max IT** | **Scan range** | | |
| **all** | 70000 | 3,00E+06 | 20 ms | 300 - 1750 m/z | | |
| | **MS2** | | | | | |
| **Experiment(s)** | **Resolution** | **AGC target** | **Max IT** | **Isolation window** | **TopN** | **NCE** |
| **Secretome and Surfactome** | 17500 | 5,00E+05 | 60 ms | 2.1 m/z | 10 | 25 |

**Supplementary Table 5:** Primers for ChIP.

| **Primers** | **Forward reaction 5'-3'** | **Reverse reaction 5'-3'** |
|---|---|---|
| *MYST1*promoter_SBE 1 | ACACCATTCTCCTGCCTCAG | GGTGGATCACGAGGTCAGA |
| *MYST1*promoter_SBE 2 | TCCCAAAGTGCTGGGATTAC | GGCAGGAGAATCGCTTGA |
| *MYST1*promoter_SBE 3 | CTCCCGAGTAGCTGGGACTA | GTGGCTCACGCCTGTAATCT |
