# Supplementary material for: TGFβ promotes fibrosis by MYST1-dependent epigenetic regulation of autophagy
Source: Nat Commun. 2021 Jul 20;12:4404. doi: 10.1038/s41467-021-24601-y (PMC8292318; doi:10.1038/s41467-021-24601-y)
Supplement: Supplementary file 2 — Reporting Summary [file 41467_2021_24601_MOESM2_ESM.pdf]

nature research

Corresponding author(s): Jörg H. W. Distler

Last updated by author(s): Mar 28, 2021

Reporting Summary

Nature Research wishes to improve the reproducibility of the work that we publish. This form provides structure for consistency and transparency in reporting. For further information on Nature Research policies, see our Editorial Policies and the Editorial Policy Checklist.

Statistics

For all statistical analyses, confirm that the following items are present in the figure legend, table legend, main text, or Methods section.

n/a	Confirmed	
☐	☒	The exact sample size (*n*) for each experimental group/condition, given as a discrete number and unit of measurement
☐	☒	A statement on whether measurements were taken from distinct samples or whether the same sample was measured repeatedly
☐	☒	The statistical test(s) used AND whether they are one- or two-sided<br>*Only common tests should be described solely by name; describe more complex techniques in the Methods section.*
☒	☐	A description of all covariates tested
☐	☒	A description of any assumptions or corrections, such as tests of normality and adjustment for multiple comparisons
☐	☒	A full description of the statistical parameters including central tendency (e.g. means) or other basic estimates (e.g. regression coefficient) AND variation (e.g. standard deviation) or associated estimates of uncertainty (e.g. confidence intervals)
☒	☐	For null hypothesis testing, the test statistic (e.g. *F*, *t*, *r*) with confidence intervals, effect sizes, degrees of freedom and *P* value noted<br>*Give P values as exact values whenever suitable.*
☒	☐	For Bayesian analysis, information on the choice of priors and Markov chain Monte Carlo settings
☒	☐	For hierarchical and complex designs, identification of the appropriate level for tests and full reporting of outcomes
☒	☐	Estimates of effect sizes (e.g. Cohen's *d*, Pearson's *r*), indicating how they were calculated

Our web collection on statistics for biologists contains articles on many of the points above.

Software and code

Policy information about availability of computer code

Data collection	StepOne software version2.3; NIS-Elements BR version 5.20.01; Leica Application Suite X version 4.12.0.86; NDP.scan 3.3; HCS Navigator Version 6.6.1; AMT Capture Engine, v602.600.51
Data analysis	Prism 8 GraphPad software Version 8.3.0; Image Lab 6.0.0; ImageJ 1.41; G*Power software 3.1; HCS Studio Cell Analysis Software 6.6.1; NDP.view2; MaxQuant software v1.5.3.8; MicroWin v4.0 Sp9; DeconvolutionLab2; Incucyte® S3 Software V2018A; Perseus version 1.6.5

For manuscripts utilizing custom algorithms or software that are central to the research but not yet described in published literature, software must be made available to editors and reviewers. We strongly encourage code deposition in a community repository (e.g. GitHub). See the Nature Research guidelines for submitting code & software for further information.

Data

Policy information about availability of data

All manuscripts must include a data availability statement. This statement should provide the following information, where applicable:

- Accession codes, unique identifiers, or web links for publicly available datasets
- A list of figures that have associated raw data
- A description of any restrictions on data availability

All data generated or analyzed during this study are included in this article and its supplementary information files. Source data are provided with this article. Uncropped western blot images are provided in the source data file. Additional detailed information is available from the corresponding author on reasonable request. The proteomics data (Supplementary figure S11 a) are available in PRIDE database with the dataset identifier PXD025001. As long as the PRIDE dataset has not been publicly released, login credentials to the PRIDE reviewer account will be provided on reasonable request.

Field-specific reporting

Please select the one below that is the best fit for your research. If you are not sure, read the appropriate sections before making your selection.

☒ Life sciences ☐ Behavioural & social sciences ☐ Ecological, evolutionary & environmental sciences

For a reference copy of the document with all sections, see nature.com/documents/nr-reporting-summary-flat.pdf

Life sciences study design

All studies must disclose on these points even when the disclosure is negative.

Sample size	The sample size was determined from preliminary experiments. No statistical method was used to predetermine sample size.
Data exclusions	No data were excluded from the analysis.
Replication	Experimental findings were reliably reproduced in at least three independent experiments.
Randomization	Mice were stratified according to sex and then randomized into the different groups. Cells and tissues from human donors were also randomized.
Blinding	Experiments and quantifications were not done in a blinded fashion.

Reporting for specific materials, systems and methods

We require information from authors about some types of materials, experimental systems and methods used in many studies. Here, indicate whether each material, system or method listed is relevant to your study. If you are not sure if a list item applies to your research, read the appropriate section before selecting a response.

Materials & experimental systems

n/a	Involved in the study	
☐	☒	Antibodies
☐	☒	Eukaryotic cell lines
☒	☐	Palaeontology and archaeology
☐	☒	Animals and other organisms
☐	☒	Human research participants
☒	☐	Clinical data
☒	☐	Dual use research of concern

Methods

n/a	Involved in the study	
☒	☐	ChIP-seq
☒	☐	Flow cytometry
☒	☐	MRI-based neuroimaging

Antibodies

Antibodies used

Primary antibodies for immunohistochemistry (IHC) and immunofluorescence (IF): α-Smooth muscle (Sigma-Aldrich, A5228, clone 1A4 or A2547, clone 1A4), ATG7 (Abcam, ab133528, clone EPR6251), ATG7 (AnaSpec, AS-54230), Beclin1 (Abcam, ab62557), SQSTM1/p62 (Abcam, ab56416), prolyl-4-hydroxylase-β (P4Hβ - Acris, TA308403), Vimentin (Abcam, ab92547, clone EPR3776), MYST1 (GeneTex, GTX83065, clone8C4C4 or Santa Cruz, sc-81163), LAMP2 (Abcam, ab13524, clone GL2A7), LC3B (Novus Biologicals, NB100-2220), GFP (Abcam, ab6673) and RFP (Abcam, ab62341).
Primary antibodies used for Extracellular matrix staining (ECM): Fibronectin antibody conjugated with Alexa Fluor 488 (eBiosciences, #53-9869-82, clone FN-3), Collagen type I (Col I) (Merck Millipore, #AB745), Collagen type III (Col III) (Merck Millipore, #AB747).

For IgG controls, Normal Rabbit IgG (Santa Cruz, sc-2027) and Normal Mouse IgG (Santa Cruz, sc-2025) were used.

For IHC secondary antibody, HRP-conjugated polyclonal goat anti-mouse (Dako, P044701-02) was used.

Secundary antibodies used for IF or ECM: AlexaFluor-conjugated 488 goat anti rabbit IgG (Invitrogen, A-11008), AlexaFluor-conjugated 488 goat anti mouse IgG (Invitrogen, A-11001), AlexaFluor-conjugated 488 goat anti rat IgG (Invitrogen, A-11006), AlexaFluor-conjugated 488 Donkey anti goat IgG (Invitrogen, AA32814), AlexaFluor-conjugated 555 goat anti mouse IgG (Invitrogen, A-21422), AlexaFluor-conjugated 555 goat anti rabbit IgG (Invitrogen, A-21428), AlexaFluor-conjugated 555 goat anti rat IgG (Invitrogen, A-21434), AlexaFluor-conjugated 647 goat anti rabbit IgG (Invitrogen, A-21244).

Primary antibodies used for western blot (WB): ATG7 (Abcam, ab133528, clone EPR6251), ATG7 (AnaSpec, AS-54230), Beclin1 (Abcam, ab62557), Collagen type I (Abcam ab138492 clone EPR7785), Collagen type I (Southern Biotech, #1310-01), FLAG Tag (Proteintech, #80010-1-PR, clone 4K14), Histone H3 (Cell Signaling, #9715), acetyl-Histone H4 (Lys16) (Merck Millipore, #07-329, Lot #2506422), LAP (R&D Systems, AF-246-NA), LC3B/MAP1LC3B (Novus Biologicals, NB 100-2220), MYST1 (GeneTex, GTX83065, clone8C4C4 or Santa Cruz, sc-81163), SQSTM1/p62 (Abcam, ab56416), pSMAD3/ SMAD3 phospho S423 + S425 (Abcam, ab52903,

clone EP823Y), SMAD3 (Cell signaling, #9523S, clone C67H9), TGFβ (Cell signaling, #3711S), β-actin (Sigma-Aldrich, A5441, clone AC-15).

Antibodies used for Chromatin Immunoprecipitation (ChIP): SMAD3 (Cell signaling, #9523S, clone C67H9) and Normal Rabbit IgG (Santa Cruz, sc-2027) as control.

HRP-conjugated secondary antibodies used for WB: polyclonal goat anti-mouse (Dako, P044701-02), polyclonal goat anti-rabbit (Dako, P044801-2) and polyclonal rabbit anti-goat (Dako, P044901-2) were used.

Validation

- α-Smooth muscle (Sigma-Aldrich, A5228 clone 1A4): The antibody (also known as anti-α-Sm-1) is specific for the single isoform of α-smooth muscle actin. Immunogen: N-terminal synthetic decapeptide of α-smooth muscle actin. Applications: immunocytochemistry (ICC), immunohistochemistry (IHC), indirect ELISA, indirect immunofluorescence, microarray, western blot (WB). Species reactivity: human, frog, sheep, chicken, goat, bovine, rat, guinea pig, mouse, canine, rabbit, snake. Molecular weight: antigen ~42 kDa. Biological source: mouse (monoclonal antibody).
- α-Smooth muscle (Sigma-Aldrich, A2547 clone 1A4): Specific for the single isoform of α-smooth muscle actin. Immunogen: N-terminal synthetic decapeptide of α-smooth muscle actin. Applications: immunohistochemistry, indirect immunofluorescence, western blot. Species reactivity: human, frog, sheep, chicken, goat, bovine, rat, guinea pig, mouse, canine, rabbit, snake. Molecular weight: antigen ~42 kDa. Biological source: mouse (monoclonal antibody).
- β-actin (Sigma-Aldrich, A5441, clone AC-15): Immunogen: slightly modified β-cytoplasmic actin N-terminal peptide, Ac-Asp-Asp-Asp-Ile-Ala-Ala-Leu-Val-Ile-Asp-Asn-Gly-Ser-Gly-Lys, conjugated to KLH. Applications: immunohistochemistry, indirect ELISA, indirect immunofluorescence: 1:1000-1:2000, western blot: 1:5000-1:10000. Species reactivity: pig, Hirudo medicinalis, bovine, rat, canine, feline, human, rabbit, carp, mouse, guinea pig, chicken, sheep. Should not react with Dictyostelium discoideum. Molecular weight: antigen ~42 kDa. Biological source: mouse (monoclonal antibody).
- ATG7 (Abcam, ab133528, clone EPR6251): Description: Rabbit monoclonal to ATG7. Immunogen: Synthetic peptide within Human ATG7 aa 1-100. The exact sequence is proprietary. Host species: Rabbit. Tested applications: WB (dilution: 1:10000 – 1:50000. Predicted molecular weight: 77 kDa. Use 5% non-fat dry milk + TBST for blocking), ICC/IF (dilution: 1:100 – 1:500). Species reactivity: Mouse, Rat, Human.
-ATG7 (AnaSpec, AS-54230): Rabbit polyclonal antibody. Species Reactivity: human and mouse, while others are not tested. Molecular weight of approximately 77 kDa on western blot. Application: ELISA for immunizing peptide: 1: 5:000-20:000 Western blot: 0.5-2.0 μg/ml, Immunocytochemistry: 5-10 μg/ml.
-BECLIN1 (Abcam, ab62557) Description: Rabbit polyclonal to Beclin 1. Host species: Rabbit: Tested applications: IHC-P, WB (Use a concentration of 0.5 - 2 μg/ml. Detects a band of approximately 52 kDa (predicted molecular weight: 52 kDa), ICC/IF (1:100). Species reactivity: Mouse, Rat, Human. Immunogen: Synthetic peptide corresponding to Human Beclin 1 (N terminal). ab62557 was raised against a 17 amino acid synthetic peptide from near the amino terminus of human Beclin-1. The immunogen is located within amino acids 40 - 90 of Beclin-1. Database: Uniprot - Q14457.
- Collagen type I (Abcam, ab138492, clone EPR7785): Description: Rabbit monoclonal to Collagen I. Host species: Rabbit. Tested applications: WB (dilution 1:1000 – 1:10000. Predicted molecular weight: 139 kDa), IHC-P (dilution 1:1500). Species reactivity: Human. Predicted to work with Cow. Immunogen: Synthetic peptide within Human Collagen I aa 1200-1300. The exact sequence is proprietary. Database: Uniprot - P02452. (Peptide available as ab198239).
-Collagen type I (Southern Biotech, 1310-01): Specificity Reacts with conformational determinants on type I collagen. Source: Pooled antisera from goats hyperimmunized with type I collagen. Cross Adsorption Collagen types II, III, IV, V, and VI. Applications: ELISA Immunohistochemistry-Paraffin Sections, Immunohistochemistry-Frozen Sections, Immunocytochemistry, Electron Microscopy, Flow Cytometry, Western Blot, Immunoprecipitation.
- Collagen type I (Merck Millipore, AB745): Application: ELISA, Immunohistochemistry, Western Blotting (1:200), ELISA on human collagen type I: 1:4,000. Indirect microimmunofluorescent visualization of collagen type I on cryostat sections of human tissue or cultured cells: 1:10-1:40. Immunogen: Human placental collagen type I. Host: Rabbit. Specificity: Recognizes Human collagen type I. Some cross reactivity with other proteins. Species Reactivity: Human. Antibody Type: Polyclonal Antibody. Entrez Gene Number: NP_000079. UniProt Number: P02452.
- Collagen type III (Merck Millipore, AB747): Application: ELISA and Immunofluorescence. Applications Not Recommended: Western Blotting and Immunohistochemistry (Paraffin). Immunogen: Human placental collagen type III. Host: Rabbit. Specificity: The antibody reacts with native and heat denatured (non-reduced) human collagen type III. Cross reactions with other types of collagen under native conditions do occur. There is 10% cross reactivity with human collagen type I, 2% cross reactivity with human collagen type II, and 4% cross reactivity with human collagen type IV and V. There is no cross reactivity with other human plasma proteins under native ELISA conditions. Reactivity under denatured conditions has not been examined. Cross Reactivity Percent: Human collagen type I 10%, Human collagen type II 2%, Human collagen type III 100%, Human collagen type IV and V 4% . Species Reactivity: Human. Antibody Type: Polyclonal Antibody. Entrez Gene Number: NM_000090.3. UniProt Number: P02461.
- Fibronectin conjugated with Alexa Fluor 488: Species Reactivity: Human. Published Species Human, Mouse. Host: Isotype Mouse / IgG1. Class: Monoclonal. Applications Immunocytochemistry (ICC), Immunofluorescence (IF) and Immunohistochemistry (IHC). The FN-3 antibody recognizes a determinant on human cellular but not plasma fibronectin; this recognition is not lost upon trypsin treatment. Furthermore, FN-3 antibody has been shown to cross react to bovine fibronectin.
- FLAG Tag (Proteintech, 80010-1-PR, clone 4K14): Source: Rabbit. Isotype: IgG. Immunogen Catalog Number: AG2329. Applications Tested Applications: IF, IP, WB, ELISA. Recommended Dilutions: WB 1:1000-1:3000, IF 1:200-1:1000. Species Specificity: Recombinant, Cited Species: human. Background Information DYKDDDDK Tag (Equivalent To FLAG Antibody From Sigma) with the following sequence DYKDDDDK, is a hydrophilic tag for recombinant protein technology. DYKDDDDK Tag Antibody is generated against 1xDYKDDDDK tag (DYKDDDDK) and can recognize protein containing one or more DDDDK tags, independently on N-terminal, C-terminal or internal regions of the target protein.
- GFP (Abcam, Ab6673): Description: Goat polyclonal to GFP. Host species: Goat. Specificity: Anti-GFP assayed by ELISA for direct binding of antigen recognizes wild type, recombinant and enhanced forms of GFP. Tested applications: WB, IP, ELISA, ICC/IF, IHC-P, IHC-FrFl, IHC-Fr. Species reactivity: Reacts with: Species independent. Immunogen: Fusion protein corresponding to Aequorea victoria GFP aa 1-246. Database: Uniprot - P42212. This anti-GFP antibody cross reacts with eGFP.
- Histone H3 (Cell signaling, 9715): Histone H3 Antibody detects endogenous levels of total histone H3 protein. This antibody does not cross-react with other histones. Application: Western Blotting 1:1000. Species Reactivity: Human, Mouse, Rat, Monkey, Zebrafish, Bovine, Pig. Species predicted to react based on 100% sequence homology: D. melanogaster. Database Links: UniProt ID: P68431,

Entrez-Gene Id: 8350. Source: Rabbit (Polyclonal).
- Acetyl-Histone H4 (Lys16) (Merck Millipore, 07-329, Lot. 2506422): Applications: Anti-acetyl-Histone H4 (Lys16) Antibody is a rabbit polyclonal antibody for detection of acetyl-Histone H4 (Lys16) also known as H4K16Ac, Histone H4 (acetyl K16 & has been published and validated in ChIP, WB, Mplex, PIA, DB, ChIP-seq. Application Notes: Chromatin Immunoprecipitation Analysis: 1 ug from a representative lot immunoprecipitated Acetyl-Histone H4 (Lys16) in HeLa chromatin. Immunocytochemistry Analysis: A 1:100 dilution from a representative lot detected Acetyl-Histone H4 (Lys16) in HeLa, A431, HUVEC, and NIH/3T3 cell lines. Dot Blot Analysis: A 1:1,000. Immunogen: KLH-conjugated linear peptide corresponding to 10 amino acids from the N-terminal region of human Histone H4 acetylated on Lysine 16. Epitope: N-terminus (Lys 16). Species Reactivity: Human, Mouse, Rat. Species Reactivity Note: Broad species cross-reactivity is expected. Entrez Gene Number: NM_175054. UniProt Number: P62805. Molecular Weight: 10 kDa.
- LAMP2 (Abcam, ab13524, clone GL2A7): Description: Rat monoclonal to LAMP2. Tested applications: WB (1:500), IF, IHC. Species reactivity. Reacts with: Mouse, Human. Immunogen: Tissue, cells or virus corresponding to Mouse LAMP2. Purified preparation of mouse liver lysosomal membranes.
- LAP (TGF-beta 1) (R&D systems, AF-246-NA): Long name: Latency-associated Peptide. Species Reactivity: Human. Specificity: Detects human LAP TGF‑ beta 1 in direct ELISAs and Western blots. Source: Polyclonal Goat IgG. Immunogen: S. frugiperda insect ovarian cell line Sf 21-derived and Chinese hamster ovary cell line CHO-derived recombinant human LAP TGF‑ beta 1 Leu30-Ser390. Accession # P01137. Applications: Western Blot (2 µg/mL), Simple Western (20 µg/mL), Immunocytochemistry (5-15 µg/mL), Immersion fixed human peripheral blood mononuclear cells, NeutralizationMeasured by its ability to neutralize LAP TGF‑ beta 1 inhibition of TGF‑ beta 1 growth inhibition in the HT‑2 mouse T cell line. Tsang, M. et al. (1995) Cytokine 7:389. The Neutralization Dose (ND50) is typically 0.4-2 µg/mL in the presence of 500 ng/mL Recombinant Human LAP TGF‑ beta 1 and 1 ng/mL TGF‑ beta 1.
- LC3B/MAP1LC3B (Novus Biologicals, NB 100-2220): Polyclonal LC3B Antibody was made to a synthetic peptide made to an N-terminal portion of the human LC3B protein sequence (between residues 1-100). [UniProt# Q9GZQ8]. Localization: Type I form of LC3B is cytoplasmic, whereas the type II form of LC3B binds to the autophagic membranes. Clonality: Polyclonal. Host: Rabbit. Applications/Dilutions: Western Blot 0.5 - 2.0 ug/mL, Simple Western 1:50, ELISA, Flow Cytometry, Immunoblotting, Immunocytochemistry/Immunofluorescence 1:200, Immunohistochemistry 1:200 - 1:400, Immunohistochemistry-Frozen, Immunohistochemistry-Paraffin 1:200 - 1:400, Immunoprecipitation 20 ug/500 ug of protein, Proximity Ligation Assay, Chromatin Immunoprecipitation (ChIP). Knockdown Validated. Theoretical Molecular Weight: 14.688 kDa. Disclaimer note: The observed molecular weight of the protein may vary from the listed predicted molecular weight due to post translational modifications, post translation cleavages, relative charges, and other experimental factors. Reactivity: Mouse, Primate, Canine, Avian ,Hamster, Rabbit, Human, Monkey, Guinea pig, Rat, Bacteria, Chicken, Golden syrian hamster, Zebrafish. Immunogen displays the following percentage of sequence identity for non-tested species: Xenopus 84%. Invertebrate reactivity reported in scientific literature (PMID: 26716072).
- MYST1 / MOF (Genetex, GTX83065, clone 8C4C4): Mouse Monoclonal antibody. Application and suggested dilutions: WB (1:500 – 1:2000), ICC/IF (1:200 – 1:1000) IHC-P 1/200 - 1/1000 ELISA 1/10000 Not tested in other applications. Reactivity: Human. Calculated Molecular weight 52 kDa. Immunogen: Purified recombinant fragment of human MYST1 expressed in E. Coli.
- MYST1 / MOF (Santa cruz, Sc-271691): MOF Antibody (G-12) is a mouse monoclonal IgG1 κ. Specific for an epitope mapping between amino acids 39-73 near the N-terminus of MOF of human origin recommended for detection of MOF of mouse, rat and human origin by WB, IP, IF and ELISA; also reactive with additional species, including and equine, canine, bovine and porcine.
- prolyl-4-hydroxylase-β (Acris, TA308403): Applications and recommended dilutions: ICC/IF (1:100-1:1000); IHC (1:100-1:1000); WB (1:500-1:10000). Reactivity: Human, Mouse (Predicted: Chimpanzee). Host: Rabbit Polyclonal. Immunogen: Recombinant fragment corresponding to a region within amino acids 23 and 227 of PDI (Uniprot ID#P07237). Predicted Protein Size: 57 kDa. Note: Seq homology of immunogen across species: Chimpanzee (100%).
- pSMAD3 (Abcam, ab52903, clone EP823Y): Rabbit monoclonal [EP823Y] to Smad3 (phospho S423 + S425). Specificity: ab52903 detects Smad3 phosphorylated on Serine 423 and Serine 425. This Smad3 antibody may also detect Smad1, Smad2 and Smad5 phosphorylated at the equivalent sites.Tested applications: WB (1:2000. Predicted molecular weight: 48 kDa), ICC/IF (1:100 – 1:250), IHC-P (1:100 – 1:250), Dot blot (1:1000). Unsuitable for: Flow Cyt or IP. Species reactivity - Reacts with: Mouse, Human. Predicted to work with: Drosophila melanogaster. Immunogen: corresponding to Human Smad3.
- RFP (Abcam, ab62341): Rabbit polyclonal to RFP. Host species: Rabbit. Specificity: ab62341 recognizes RFP and has been shown to react with tdTomato. Tested applications: WB (Use a concentration of 0.5 - 4 µg/ml. Predicted molecular weight: 27.6 kDa). Species reactivity: Recombinant fragment. Immunogen: Recombinant full length protein corresponding to RFP.
- SMAD3 (Cell signaling, 9523S, clone C67H9): Specificity / Sensitivity: Smad3 (C67H9) Rabbit mAb detects endogenous levels of total Smad3 protein. No cross reactivity was detected with other family members. Species Reactivity: Human, Mouse, Rat, Monkey. Species predicted to react based on 100% sequence homology: Xenopus, Zebrafish, Bovine. For optimal ChIP and ChIP-seq results, use 10 µl of antibody and 10 µg of chromatin (approximately 4 x 106 cells) per IP. This antibody has been validated using SimpleChIP® Enzymatic Chromatin IP Kits. Applications and dilutions: Western Blotting (1:1000), Immunoprecipitation (1:100), Immunofluorescence (Immunocytochemistry - 1:100), Flow Cytometry (1:100). Chromatin IP (1:50), Chromatin IP-seq (1:50). Molecular weight: 52 kDa.
- SQSTM1 / p62 (Abcam, ab56416): Mouse monoclonal. Host species: Mouse. Tested applications : IHC-P, WB, ICC/IF, Flow Cyt. Species reactivity: Human. Immunogen: Recombinant full length protein corresponding to Human SQSTM1/ p62 aa 1-440. Database: Uniprot - Q13501. Knockout validated. Predicted band size: 47 kDa. Observed band size: 64 kDa
- TGFβ (Cell signaling, 3711S): Application and dilution: Western Blotting (1:1000). Specificity / Sensitivity: TGF-beta Antibody detects recombinant TGF-β1, TGF-β2, and TGF-β3. The antibody also detects endogenous levels of the TGF-β1 precursor proteins. Species Reactivity: Human, Mouse, Rat.
- Vimentin (Abcam, ab92547, clone EPR3776): Rabbit monoclonal [EPR3776] to Vimentin - Cytoskeleton Marker. Suitable for: ICC/IF (1:250 – 1:1000), WB (1:1000 – 1:5000. Predicted molecular weight: 54 kDa), Flow Cyt (1:50 – 1:500), IHC-P (1:200 – 1:500). Knockout validated. Reacts with: Mouse, Rat, Human, African green monkey.

Eukaryotic cell lines

Policy information about cell lines

Cell line source(s)

- Mink lung cell (MLC) was provided by Dr. Daniel Rifkin, Department of Cell Biology and Kaplan Cancer Center, New York University Medical Center, New York, USA. This cell line was generated by his group and published in Abe et al., 1993.
- NIH3T3 was obtained from DSMZ-German Collection of Microorganisms and Cell Cultures.

	- Wi-26 human lung fibroblasts: Wi-26 with stable knockout of ATG7, clones three and seven, were generated and provided by PD. Dr. Markus Plomann and Dr. Beate Eckes, Center for Biochemistry and Department of Dermatology, University of Cologne, Cologne, Germany.<br>- Atg7 knockout murine fibroblasts: Murine fibroblasts were isolated from skin biopsies of mice expressing two conditional alleles of Atg7 (Atg7fl/fl). Mice were kindly provided by Dr. Masaaki Komatsu, Tokyo Metropolitan Institute of Medical Science, Tokyo, Japan. The Atg7 knockout mice was generated by Komatsu´s group and published in Komatsu et al, 2005.
Authentication	None of the cell lines used were authenticated.
Mycoplasma contamination	All cell lines were tested negative for mycoplasma contamination by PCR assays.
Commonly misidentified lines<br>(See ICLAC register)	No commonly misidentified cell lines were used.

Animals and other organisms

Policy information about studies involving animals; ARRIVE guidelines recommended for reporting animal research

Laboratory animals	Male and female C57BL/6NRj (Age 6 to 8 weeks old), Atg7fl/fl X Col1a2CreER (6 to 8 weeks old), Atg7fl/fl X Col6Cre mice (6 to 8 weeks old) were used in this study. Housing conditions: Temperature: 20 - 24°C; Humidity: 45 - 65%, 12/12 hours light/dark cycle.
Wild animals	This study did not involve wild animals.
Field-collected samples	This study did not involve field-collected samples.
Ethics oversight	All animal experiments were approved by the governments of Mittelfranken or Unterfranken, Germany. All animal experiments were performed in compliance with the relevant ethical regulations of the governments of Mittelfranken or Unterfranken, Germany.

Note that full information on the approval of the study protocol must also be provided in the manuscript.

Human research participants

Policy information about studies involving human research participants

Population characteristics	Skin biopsies of a total of 23 systemic sclerosis (SSc) patients and 12 age- and sex-matched healthy volunteers. All patients fulfilled the 2013 American College of Rheumatology (ACR)/ European League Against Rheumatism (EULAR) criteria for SSc.
Recruitment	Human samples were obtained from research volunteers of the University Hospital Erlangen. Written informed consents were obtained from all subjects. There was no self-selection bias involved.
Ethics oversight	The human studies were approved by the Ethical committee of the Medical Faculty of the University of Erlangen-Nuremberg.

Note that full information on the approval of the study protocol must also be provided in the manuscript.
